# Supplementary material for: Determinants of the intensity of uremic pruritus in patients receiving maintenance hemodialysis: A cross-sectional study
Source: PLoS One. 2021 Jan 20;16(1):e0245370. doi: 10.1371/journal.pone.0245370 (PMC7817000; doi:10.1371/journal.pone.0245370)
Supplement: S1 Table — (DOCX) [file pone.0245370.s001.docx]

**S1 Table. Single-factor logistic-regression analysis of degree of skin itching in patients with uremic pruritus.**

| **Skin itching^a^** | **Variable** | **B** | **SE** | **Forest** | **df** | ***P*** | **OR** | **95% CI** |
| --- | --- | --- | --- | --- | --- | --- | --- | --- |
| Mild | hs-CRP* | 0.651 | 0.252 | 6.655 | 1 | 0.010 | 1.917 | 1.169–3.144 |
|  | PTH | 0.001 | 0.001 | 2.760 | 1 | 0.097 | 1.001 | 1.000–1.002 |
|  | Hb | 0.014 | 0.018 | 0.608 | 1 | 0.435 | 1.014 | 0.979–1.049 |
| Moderate | hs-CRP* | 0.992 | 0.229 | 18.703 | 1 | <0.001 | 2.696 | 1.720–4.226 |
|  | PTH | 0.001 | 0.001 | 2.771 | 1 | 0.096 | 1.001 | 1.000–1.002 |
|  | Hb* | 0.035 | 0.016 | 4.957 | 1 | 0.026 | 1.036 | 1.004–1.068 |
| Severe | hs-CRP* | 1.934 | 0.401 | 23.286 | 1 | <0.001 | 6.915 | 3.153–15.167 |
|  | PTH* | 0.001 | 0.001 | 4.626 | 1 | 0.031 | 1.001 | 1.000–1.003 |
|  | Hb* | 0.036 | 0.026 | 1.886 | 1 | 0.170 | 1.036 | 0.985–1.091 |

Notes: Using ^a^no itching, ^b^urban residence as a reference. SE, standard error; df, degrees of freedom; OR, odds ratio; CI, confidence interval; hs-CRP, serum hypersensitive C-reactive protein; PTH, serum parathyroid hormone; Hb, hemoglobin. **P* < 0.05.
