# Supplementary material for: Determinants of the intensity of uremic pruritus in patients receiving maintenance hemodialysis: A cross-sectional study
Source: PLoS One. 2021 Jan 20;16(1):e0245370. doi: 10.1371/journal.pone.0245370 (PMC7817000; doi:10.1371/journal.pone.0245370)
Supplement: S2 Table — (DOCX) [file pone.0245370.s002.docx]

**S2 Table. Multiple logistic-regression analysis of factors influencing skin itching in patients with uremic pruritus.**

| **Skin itching**^a^ | **Variable** | **B** | **SE** | **Forest** | **df** | ***P*** | **OR** | **95% CI** |
| --- | --- | --- | --- | --- | --- | --- | --- | --- |
| Mild | hs-CRP* | 0.554 | 0.252 | 4.808 | 1 | 0.028 | 1.740 | 1.061–2.854 |
|  | PTH | 0.001 | 0.001 | 2.480 | 1 | 0.115 | 1.001 | 1.000–1.002 |
|  | Hb | 0.010 | 0.019 | 0.286 | 1 | 0.593 | 1.010 | 0.973–1.049 |
|  | Rural resident^b^ | −0.744 | 0.507 | 2.156 | 1 | 0.142 | 0.475 | 0.176–1.283 |
| Moderate | hs-CRP* | 1.043 | 0.248 | 17.631 | 1 | <0.001 | 2.838 | 1.744–4.618 |
|  | PTH | 0.001 | 0.001 | 0.907 | 1 | 0.341 | 1.001 | 0.999–1.002 |
|  | Hb | 0.020 | 0.016 | 1.491 | 1 | 0.222 | 1.020 | 0.988–1.054 |
|  | Rural resident^b*^ | 1.353 | 0.642 | 4.440 | 1 | 0.035 | 3.869 | 1.099–13.622 |
| Severe | hs-CRP* | 2.245 | 0.499 | 20.203 | 1 | <0.001 | 9.440 | 3.547–25.124 |
|  | PTH | 0.001 | 0.001 | 1.143 | 1 | 0.285 | 1.001 | 0.999–1.003 |
|  | Hb | -0.009 | 0.036 | 0.064 | 1 | 0.800 | 0.991 | 0.924–1.063 |
|  | Rural resident^b^ | 2.672 | 1.450 | 3.396 | 1 | 0.065 | 14.468 | 0.844–248.035 |

Notes: Using ^a^no itching, ^b^urban residence as a reference. SE, standard error; df, degrees of freedom; OR, odds ratio; CI, confidence interval; hs-CRP, serum hypersensitive C-reactive protein; PTH, serum parathyroid hormone; Hb, hemoglobin; P, phosphorus. **P* < 0.05.
