# Supplementary material for: Determinants of the intensity of uremic pruritus in patients receiving maintenance hemodialysis: A cross-sectional study
Source: PLoS One. 2021 Jan 20;16(1):e0245370. doi: 10.1371/journal.pone.0245370 (PMC7817000; doi:10.1371/journal.pone.0245370)
Supplement: Attachment — Submitted filename: PONE-D-20-18098_with minor observations.pdf [file pone.0245370.s003.pdf]

PLOS ONE

Determinants of the intensity of uremic pruritus in patients receiving maintenance hemodialysis: a corss-sectional study

--Manuscript Draft--

Manuscript Number:	PONE-D-20-18098
Article Type:	Research Article
Full Title:	Determinants of the intensity of uremic pruritus in patients receiving maintenance hemodialysis: a corss-sectional study
Short Title:	Determinants of the intensity of uremic pruritus in Hemodialysis patients
Corresponding Author:	Jian-Hui Zhao, M.M.<br>Xinching Country People's Hospital<br>CHINA
Keywords:	Hypersensitive C-reactive protein; Uremic pruritus; The intensity of skin itching; Maintenance hemodialysis
Abstract:	Background<br><br>Uremic pruritus (UP) is a common and frustrating symptom in patients receiving hemodialysis (HD). This study aimed to investigate the determinants of the intensity of UP in patients receiving maintenance HD.<br><br>Methods<br><br>A cross-sectional study on uremic pruritus in male and female patients receiving HD was conducted in September 2019.This study included 148 eligible patients who received HD at the Blood Purification Center of Xinchang County People's Hospital, Zhejiang Province, China from March 2019 to June 2019. We collected general data such as age, sex, body mass index (BMI), place of residence, educational level, and duration of HD; as well as clinical biochemical indicators, including serum calcium (Ca), serum phosphorus (P), serum albumin (ALB), hemoglobin (Hb), serum parathyroid hormone (PTH), and pre-dialysis serum creatinine (sCr). We also assayed the inflammatory cytokine serum C-reactive protein (CRP). The Five-Dimensional Itching Scale (5DIS) was used to evaluate the degree of skin itching (none, mild, moderate, or severe). We used multiple logistic regression to analyze influencing factors on the degree of skin itching in patients with UP.<br><br>Results<br><br>(1) Of the 148 patients, 60 had uremic pruritus (incidence rate, 40.54%). These included 22 cases of mild skin itching (14.86%), 30 of moderate skin itching (20.27%), and 8 of severe skin itching (5.41%). (2) Compared with uremia patients without skin pruritus, patients with UP had higher levels of PTH, Hb, and serum hypersensitive C-reactive protein (hs-CRP). (3) The composition ratio showed significant differences between urban and rural patients with different degrees of skin itching (P = 0.017); moreover, PTH and hs-CRP levels differed to a statistically significant degree (P = 0.015 and < 0.001). (4) Multiple logistic-regression analysis showed that as hs-CRP level increased, the patient's risks of mild skin itching (odds ratio [OR] = 1.720; 95% confidence interval [CI], 1.044–2.835; P = 0.033), moderate skin itching (OR = 2.884; 95% CI, 1.764–4.716; P < 0.001), and severe skin itching (OR = 8.929; 95% CI, 3.340–23.865; P < 0.001) all increased as well. Compared with urban residents, rural residents have a higher risk of moderate itching (OR = 3.942; 95% CI, 1.105–14.069; P=0.035).<br><br>Conclusion<br><br>Levels of hs-CRP were associated with degree of skin itching in patients with UP. Higher hs-CRP levels were closely related to severe skin itching.
Order of Authors:	Jian-Hui Zhao, M.M.

Powered by Editorial Manager® and ProduXion Manager® from Aries Systems Corporation

	Qiu-Shuang Zhu
	Yi-Wen Li
	Li-Li Wang
**Additional Information:**	
**Question**	**Response**
**Financial Disclosure**<br><br>Enter a financial disclosure statement that describes the sources of funding for the work included in this submission. Review the submission guidelines for detailed requirements. View published research articles from *PLOS ONE* for specific examples.<br><br>This statement is required for submission and **will appear in the published article** if the submission is accepted. Please make sure it is accurate.<br><br>**Unfunded studies**<br>Enter: *The author(s) received no specific funding for this work.*<br><br>**Funded studies**<br>Enter a statement with the following details:<br>• Initials of the authors who received each award<br>• Grant numbers awarded to each author<br>• The full name of each funder<br>• URL of each funder website<br>• Did the sponsors or funders play any role in the study design, data collection and analysis, decision to publish, or preparation of the manuscript?<br>• **NO** - Include this sentence at the end of your statement: *The funders had no role in study design, data collection and analysis, decision to publish, or preparation of the manuscript.*<br>• **YES** - Specify the role(s) played.<br><br>* typeset	The authors received no specific funding for this work.
**Competing Interests**<br><br>Use the instructions below to enter a competing interest statement for this submission. On behalf of all authors, disclose any competing interests that could be perceived to bias this	The authors have declared that no competing interests exist.

work—acknowledging all financial support and any other relevant financial or non-financial competing interests.<br><br>This statement **will appear in the published article** if the submission is accepted. Please make sure it is accurate. View published research articles from *PLOS ONE* for specific examples.<br><br>**NO authors have competing interests**<br><br>Enter: *The authors have declared that no competing interests exist.*<br><br>**Authors with competing interests**<br><br>Enter competing interest details beginning with this statement:<br><br>*I have read the journal's policy and the authors of this manuscript have the following competing interests: [insert competing interests here]*<br><br>* typeset	
**Ethics Statement**<br><br>Enter an ethics statement for this submission. This statement is required if the study involved:<br><br>• Human participants<br>• Human specimens or tissue<br>• Vertebrate animals or cephalopods<br>• Vertebrate embryos or tissues<br>• Field research<br><br>Write "N/A" if the submission does not require an ethics statement.<br><br>General guidance is provided below. Consult the submission guidelines for detailed instructions. **Make sure that all information entered here is included in the Methods section of the manuscript.**	name of institutional committee: XinChang County People's Hospital Ethics Committee.<br>approval number: XCXRMYY2018-001.<br>Written consent.

Powered by Editorial Manager® and ProduXion Manager® from Aries Systems Corporation

**Format for specific study types**<br><br>**Human Subject Research (involving human participants and/or tissue)**<br>• Give the name of the institutional review board or ethics committee that approved the study<br>• Include the approval number and/or a statement indicating approval of this research<br>• Indicate the form of consent obtained (written/oral) or the reason that consent was not obtained (e.g. the data were analyzed anonymously)<br><br>**Animal Research (involving vertebrate animals, embryos or tissues)**<br>• Provide the name of the Institutional Animal Care and Use Committee (IACUC) or other relevant ethics board that reviewed the study protocol, and indicate whether they approved this research or granted a formal waiver of ethical approval<br>• Include an approval number if one was obtained<br>• If the study involved *non-human primates*, add *additional details* about animal welfare and steps taken to ameliorate suffering<br>• If anesthesia, euthanasia, or any kind of animal sacrifice is part of the study, include briefly which substances and/or methods were applied<br><br>**Field Research**<br><br>Include the following details if this study involves the collection of plant, animal, or other materials from a natural setting:<br>• Field permit number<br>• Name of the institution or relevant body that granted permission	
**Data Availability**<br><br>Authors are required to make all data underlying the findings described fully available, without restriction, and from the time of publication. PLOS allows rare exceptions to address legal and ethical concerns. See the PLOS Data Policy and FAQ for detailed information.	Yes - all data are fully available without restriction

A Data Availability Statement describing where the data can be found is required at submission. Your answers to this question constitute the Data Availability Statement and **will be published in the article**, if accepted.<br><br>**Important:** Stating 'data available on request from the author' is not sufficient. If your data are only available upon request, select 'No' for the first question and explain your exceptional situation in the text box.<br><br>Do the authors confirm that all data underlying the findings described in their manuscript are fully available without restriction?	
**Describe where the data may be found in full sentences. If you are copying our sample text, replace any instances of XXX with the appropriate details.**<br><br>• If the data are **held or will be held in a public repository**, include URLs, accession numbers or DOIs. If this information will only be available after acceptance, indicate this by ticking the box below. For example: *All XXX files are available from the XXX database (accession number(s) XXX, XXX.*).<br>• If the data are all contained **within the manuscript and/or Supporting Information files**, enter the following: *All relevant data are within the manuscript and its Supporting Information files.*<br>• If neither of these applies but you are able to provide **details of access elsewhere**, with or without limitations, please do so. For example:<br><br>*Data cannot be shared publicly because of [XXX]. Data are available from the XXX Institutional Data Access / Ethics Committee (contact via XXX) for researchers who meet the criteria for access to confidential data.*<br><br>*The data underlying the results presented in the study are available from (include the name of the third party*	All relevant data are within the manuscript and its Supporting Information files.

and contact information or URL).<br>• This text is appropriate if the data are owned by a third party and authors do not have permission to share the data.<br><br>* typeset	
Additional data availability information:	Tick here if the URLs/accession numbers/DOIs will be available only after acceptance of the manuscript for publication so that we can ensure their inclusion before publication.

Powered by Editorial Manager® and ProduXion Manager® from Aries Systems Corporation

Manuscript Click here to access/download;Manuscript;manuscript.docx

Determinants of the intensity of uremic pruritus in patients receiving maintenance hemodialysis: A cross-sectional study

Jian-Hui Zhao[1,2*], Qiu-Shuang Zhu [1,2], Yi-Wen Li[3], Li-Li Wang[2]

1Department of Nephrology, Xinchang County People's Hospital, Zhejiang Province, Xinchang 312500, 2 Blood Purification Center, Xinchang County People's Hospital, Zhejiang Province, Xinchang 312500, 3 Department of Nephrology, Zhejiang People's Hospital, Hangzhou, Zhejiang Province, 310000

* lyzhaojh@126.com

Abstract

Background: Uremic pruritus (UP) is a common and frustrating symptom in patients receiving hemodialysis (HD). This study aimed to investigate the determinants of the intensity of UP in patients receiving maintenance HD.

Methods: A cross-sectional study on uremic pruritus in male and female patients receiving HD was conducted in September 2019.This study included 148 eligible patients who received HD at the Blood Purification Center of Xinchang County People's Hospital, Zhejiang Province, China from March 2019 to June 2019. We collected general data such as age, sex, body mass index (BMI), place of residence, educational level, and duration of HD; as well as clinical biochemical indicators, including serum calcium (Ca), serum phosphorus (P), serum albumin (ALB), hemoglobin (Hb), serum parathyroid hormone (PTH), and pre-dialysis serum creatinine (sCr). We also assayed the inflammatory cytokine serum C-reactive protein (CRP). The Five-Dimensional Itching Scale (5DIS) was used to evaluate the degree of skin itching (none, mild, moderate, or severe). We used multiple logistic regression to analyze influencing factors on the degree of skin itching in patients with UP.

Results: (1) Of the 148 patients, 60 had uremic pruritus (incidence rate, 40.54%). These included 22 cases of mild skin itching (14.86%), 30 of moderate skin itching (20.27%), and 8 of severe skin itching (5.41%). (2) Compared with uremia patients without skin pruritus, patients with UP had higher levels of PTH, Hb, and serum hypersensitive C-reactive protein (hs-CRP). (3) The composition ratio showed significant differences between urban and rural patients with different degrees of skin itching (P = 0.017); moreover, PTH and hs-CRP levels differed to a statistically significant degree (P = 0.015 and < 0.001). (4) Multiple logistic-regression analysis showed that as hs-CRP level increased, the patient's risks of mild skin itching (odds ratio [OR] = 1.720; 95% confidence interval [CI], 1.044–2.835; P = 0.033), moderate skin itching (OR = 2.884; 95% CI, 1.764–4.716; P < 0.001), and severe skin itching (OR = 8.929; 95% CI, 3.340–23.865; P < 0.001) all increased as well. Compared with urban residents, rural residents have a higher risk of moderate itching (OR = 3.942; 95% CI, 1.105–14.069; P=0.035).

Conclusion: Levels of hs-CRP were associated with degree of skin itching in patients with UP. Higher hs-CRP levels were closely related to severe skin itching.

Introduction

Itchy skin is a common and distressing symptom in patients with chronic kidney disease. Uremic pruritus mainly manifests as skin itching of varying degrees, either systemically or locally, of which the back, extremities, chest, and head are common. Itching appears as paroxysmal episodes of varying duration. Those with milder symptoms can have intermittent attacks, lasting several minutes each time, while those with more severe symptoms last longer, and the symptoms are usually most obvious at night[1]. Epidemiological data indicated that approximately 40% of patients with end-stage renal disease (ESRD) experience moderate to

severe itching[2]. The Dialysis Outcomes Practice Patterns Study (DOPPS), conducted from 2012 to 2015, showed that 26–48% of patients had at least moderate skin itching, whereas 13–26% of patients had severe or extreme itching[3]. The higher the degree of skin itching, the higher the mortality rate[4]. Several causes of or factors contributing to uremic pruritus (UP) have been proposed, including increasing systemic inflammation; abnormal levels of serum parathyroid hormone (PTH), serum calcium (Ca), and serum phosphorus (P); opioid receptor imbalance; and neuropathological processes[2]. However, the factors affecting degree of skin itching in patients with uremia are still unclear. Compared with peritoneal-dialysis patients, hemodialysis (HD) patients tend to have itchier skin[5]. At present, only a few studies have explored the factors affecting skin itching intensity in these patients. Intensity of skin itching has a significant negative correlation with expression of κ opioid receptors in patients with HD[6]. In addition, serum hypersensitiv[illegible]C[illegible]eactive protein (hs-CRP) levels, male sex, HD duration, insulin resistance, insufficient dialysis adequacy, and hyperphosphatemia are positively correlated with UP intensity. Hepatitis C virus infection is also associated with serious UP[7]. Increasing the blood flow of the HD machine can reduce the intensity and frequency of skin itching[8]. However, the above preliminary research used visual-analogue scoring (VAS) to evaluate the degree of skin itching and investigated the phenomenon across only a few parameters. To produce more objective and comprehensive results, we used a five-dimensional itching scale[9] to evaluate skin itching across multiple dimensions in uremic patients undergoing maintenance HD. We included general patient data such as age, sex, body mass index (BMI), place of residence, educational level, and duration of HD; as well as clinical indicators such as Ca, P, serum albumin (ALB), hemoglobin (Hb), PTH, serum creatinine (sCr) before dialysis, and hs-CRP to analyze the determinants of itching in these patients. The aims of this study are to:

1.explore the prevalence of UP in maintenance HD patients and the demographic and clinical characteristics of pruritus patients.

2.explore determinants of higher pruritus intensity based on participants' clinical and biochemical parameters and their pruritus assessments by 5DIS at baseline.

Materials and methods

participants and data collection

This is a cross-sectional study on UP in patients receiving HD. we compiled 160 cases of maintenance HD patients who were treated from March 2019 to June 2019 according to sampling survey, 3 of whom did not respond. We excluded one case of cirrhosis, one of dialysis allergy, one of drug rash, three of nodule itch, and three of hearing impairment that rendered the patient unable to communicate; ultimately, we included a total of 148 subjects, and each of them signed an informed consent form (Fig 1). Inclusion criteria were as follows: (1) ≥1 month of HD in dialysis units and (2) age 20–90 years. Exclusion criteria were as follows: (1) primary skin diseases (e.g., eczema, psoriasis, neurodermatitis, allergic dermatitis, and drug rash), (2) use of right-cyclic saccharin, analgesics, antibiotics, or lipid-lowering drugs, (3) peripheral neuropathy, thyroid disease, leukemia, lymphoma, bile siltation, liver lesions, or pregnancy, (4) patients with hearing/communication impairments who could not complete the study, and (5) patients with infections, malignant tumors, cardiovascular disease, or tissue damage. Collection of data was by the authors or research personnel and all personnel received training and guidance from the first author. Patient data included sex, age, BMI, place of residence (rural or urban), educational level (illiterate or semi-literate, primary, junior high school, high school, or university or above), and duration of maintenance HD (months), which were obtained by the investigator

interviewing the patients. We assayed biochemical indicators such as Ca, P, PTH, Hb, ALB, sCr, and hs-CR[illegible]l of the laboratory tests were performed by the hospital's central laboratory, and auto-analyzers were used to determine biochemical data. The clinical biochemical indicators are based on the data within 3 months from the survey day. If there is no data within 3 months, the patient is required to re-test. If the patient is unwilling to cooperate with the test, it is a missing value. BMI (kg/m^2) was calculated using the following formula: weight (kg) / height (m)^2. Lean patients were defined as those with BMI ≦18.4 kg/m^2, normal patients with BMI 18.5–23.9 kg/m^2, heavy patients with BMI 24.0–27.9 kg/m^2, and obese patients with BMI ≧8.0 kg/m^2. According to World Health Organization (WHO) standards, elderly patients were defined as those age ≧65, and non-elderly patients as those age <65.

Fig 1. Patient screening and grouping.

Determining UP

The diagnostic criteria for uremic pruritus adopted international[illegible]e as follows: (1) uremia patients exclude skin pruritus caused by other diseases; (2) itching occurs at least 3 days within 2 weeks, and itches several times a day, each time itching It lasts a few minutes and affects the patient's life; (3) Itching in a specific pattern lasts for more than 6 months[10]. We used the Five-Dimensional Itching Scale (5DIS) to assess itching based on the five dimensions of degree, duration, direction, disability, and distribution. Total score ranged from 5 (no itching) to 25 (most severe itching). Patients with scores <5 points were defined as having no itching; those with scores 6–25 points were considered itch patients. Score of 6–10 points indicated mild itching, 11–20 points indicated moderate itching, and 21–25 points indicated severe Itching. Diagnosis of uremia pruritus meets the following conditions: 1. The total score of pruritus according to 5DIS is between 6-25 points. 2. Meet the international diagnostic criteria for uremic pruritus (2) and (3). 3. Meet the exclusion criteria. ultimately, we included a total of 60 patients with UP and 88 patients without UP.

Study size

Epidemiological data indicated that approximately 40% of patients with end-stage renal disease (ESRD) experience UP[2]. The overall prevalence of uremia and pruritus is 0.4, the tolerance is set to 0.1, taking a=0.05, the total number of hemodialysis in our blood purification center is 180 people. According to PASS 15 Power Analysis and Sample Size Software (NCSS, LLC. Kaysville, Utah, USA, ncss.com/software/pass), the calculated sample size is 121 people, assuming that the dropout rate is 20%, and the total sample size is 152 people.

Ethics

This study was approved by the Ethics Committee of Xinchang County People's Hospital, Zhejiang Province, China (Approval No.XCXRMYY2018-001).

Statistical analysis

We performed statistical processing using line data from SPSS software version 24.0 (IBM Corp., Armonk, New York, US). Quantitative data with a normal distribution are expressed as x±[illegible]tergroup comparisons were made using One-way analysis of variance or t test; quantitative data with a non-normal distribution are expressed as medians and interquartile ranges, intergroup comparisons of continuous variables were made using a non-parametric test. intergroup comparison of classification variables was based on the rank-sum or chi-square test. A multiple logistic regression model analyzed the factors associated with degree of skin itching in patients with uremia. All variables were diagnosed by collinearity; after elimination of the collinearity problem, variables of $P \leqq 0.10$ in single-factor

regression analysis and variables with statistical differences between patients with different degrees of skin itching were included in the regression model. Finally, we included five factors including Hb, P, PTH, hs-CRP, place of residence in the multiple logistic-regression model. $P < 0.05$ was considered to be statistically significant. In this study, both the variables PTH and hs-CRP have 2 missing values, a total of 4 cases had missing values. Although the analysis of missing values indicates that the variables PTH and hs-CRP do not rule out MCAR (missing completely at random), this study still uses the EM (expectation maximization) algorithm to fill in cautiously.

Results

Baseline characteristics of included patients

We recruited 160hemodialysis patients, 3 of those did not respond, excluded 9 people, and finally included 148 patients in the study. 89 males (60.1%) and 60 females (39.9%). Median duration of HD was 42 months. There were 60 cases of UP (prevalence rate, 40.54%), including 22 mild cases (14.86%), 30 moderate cases (20.27%), and 8 severe cases (5.41%), As shown in Table 1.

Table 1. Demographic and clinical characteristics of patients enrolled at baseline.

Variable	Results
Sex	
Male	89 (60.1%)
Female	59 (39.9%)
Age, years	
Non-elderly (<65 years)	99 (66.9%)
Elderly (≥65 years)	49 (33.1%)
Educational level	
Illiterate or semi-literate	23 (15.5%)
Primary school	36 (24.3%)
Junior high school	54 (36.3%)
High school	19 (12.8%)
University and above	16 (10.8%)
Place of residence	
Rural	100 (67.6%)
Urban	48 (32.4%)
BMI, kg/m²	
≦18.4	25 (16.9%)
18.5–23.9	99 (66.9%)
24–27.9	17 (11.5%)
≧28	7 (4.7%)
Ca, mmol/l	2.23 (2.10–2.34)
P, mmol/l	1.74 (1.49–2.22)

PTH, pg/mL	299.50 (151.50–581.50)
Hb, g/L	102.00 (95.25–110.00)
ALB, g/L	38.25 (35.20–40.00)
sCR, µmol/L	840.00 (666.00–910.00)
hs-CRP, mg/L	1.11 (0.60–2.00)
Duration of HD, months	42.00 (18.50–77.25)
Degree of skin itching	60 (40.5%)
Mild	22 (14.86%)
Moderate	30 (20.27%)
Severe	8 (5.41%)

Notes: BMI, body mass index; Ca, serum calcium; P, serum phosphorus; PTH, serum parathyroid hormone; Hb, hemoglobin; ALB, serum albumin; sCr, serum creatinine; hs-CRP, serum hypersensitive C-reactive protein.

Comparison of patients with and without skin pruritus

Compared with uremia patients without skin pruritus, patients with UP had higher levels of PTH, Hb, and hs-CRP. No statistically significant differences were found in demographic characteristics such as sex, age, educational level, BMI, or residence; in levels of Ca, P, ALB, or sCr; or in HD duration (Table 2).

Table 2. Demographic and clinical characteristics of patients with and without uremic pruritus.

Variable	Pruritic group (n = 60)	Non-pruritic group (n = 88)	*P*
Sex			0.318
Male	39 (65%)	50 (56.8%)	
Female	21 (35%)	38 (43.2%)	
Age, years			0.083
<65 years	45 (75%)	54 (61.4%)	
≥65 years	15 (15%)	34 (38.6%)	
Educational level			0.623
Illiterate or semi-literate	7 (11.7%)	16 (18.2%)	
Primary school	13 (21.7%)	23 (26.2%)	
Junior high school	28 (46.7%)	26 (29.5%)	
High school	8 (13.3%)	11 (12.5%)	
University and above	4 (6.7%)	12 (13.6%)	
Place of residence			0.602
Rural	42 (70%)	58 (65.9%)	
Urban	18 (30%)	30 (34.1%)	
BMI, kg/m^2			0.051
≦18.4	13 (21.7%)	12 (13.6%)	
18.5–23.9	41 (68.3%)	58 (65.9%)	
24–27.9	5 (8.3%)	12 (13.6%)	
≥28	1 (1.7%)	6 (6.8%)	
Ca, mmol/l	2.24 (2.13–2.35)	2.19 (2.09–2.34)	0.211
P, mmol/l	1.85 (1.51–2.32)	1.72 (1.45–2.14)	0.117
PTH, pg/ml	368.00 (220.50–746.50)	220.50 (111.50–502.50)	0.001*
Hb, g/L	104.00 (98.50–111.50)	100.00 (91.00–109.00)	0.025*
ALB, g/L	38.10 (35.15–40.00)	38.45 (35.80–40.05)	0.972
sCr, μmol/L	853.00 (686.00–950.00)	824.00 (634.50–899.50)	0.420
hs-CRP, mg/L	2.00 (1.05–3.06)	0.90 (0.50–1.50)	<0.001*
Duration of HD, months	45.50 (18.00–76.00)	42.00 (20.50–77.50)	0.936

Notes: The above numerical variables are all non-normal distribution data, articulated by median (range). Intergroup comparisons of variables were made using the Mann–Whitney *U* test.

Categorical variables are expressed as counts (percentages). Unordered categorical variables were tested by chi-square; ordered categorical variables such as BMI and educational level were tested by Kruskal–Wallis *H*. BMI, body mass index; Ca, serum calcium; P, serum phosphorus; PTH, serum parathyroid hormone; Hb, hemoglobin; ALB, serum albumin; sCr, serum creatinine; hs-CRP, serum hypersensitive C-reactive protein.*$P < 0.05$.

Comparison of patients with different degrees of skin itching

The composition ratio showed a difference between rural and urban patients with varying degrees of skin itching ($P = 0.017$). Differences in PTH and hs-CRP levels of patients with varying degrees of skin itching were statistically significant ($P = 0.015$ and <0.001, respectively), As shown in Table 3. Further comparison between the two groups found that compared with mild skin itching, the proportion of rural residents with moderate skin itching was significantly higher than that of urban residents ($P = 0.004$). Levels of hs-CRP in patients with moderate and severe skin itching were statistically significantly higher than in patients without itching (all $P < 0.001$); the level of hs-CRP in patients with severe itching was higher than in those with mild skin itching, to a statistically significant degree ($P = 0.007$). There was no statistically significant difference in PTH levels in patients with severe itching (all $P > 0.05$), As shown in S1 Fig.

Table 3. Baseline characteristics of patients with varying degrees of skin itching.

Variable	No itching (n = 88)	Mild itching (n = 22)	Moderate itching (n = 30)	Severe itching (n = 8)	*P*
Sex					
Male	50 (56.8%)	16 (72.7%)	19 (63.3)	4 (50%)	0.495
Female	38 (43.2%)	6 (27.3%)	11 (36.7)	4 (50%)	
Age, years					
<65	54 (61. 4%)	15 (68.2%)	24 (80.0%)	6 (75.0%)	0.262
≥65	34 (38.6%)	7 (31.8%)	6 (20.0%)	2 (25.0%)	
Educational level					
Illiterate/semi-literate	16 (18.2%)	2 (9.1%)	4 (13.3%)	1 (12.5%)	0.478
Primary school	23 (26.1)	3 (13.6%)	8 (26.7%)	2 (25%)	
Junior high school	26 (29. 5%)	11 (50.0%)	12 (40.0%)	5 (62.5%)	
High school	11 (12.5%)	2 (9.1%)	6 (20.0%)	0 (0.0%)	

University and above	12 (13.6%)	4 (18.2%)	0 (0.0%)	0 (0.0%)	
Place of residence					
Rural	58 (65.9%)	10 (45.5%)	25 (83.3%)	7 (87.5%)	0.017*
Urban	30 (34.1%)	12 (54.5%)	5 (16.7%)	1 (12.5%)	
BMI, kg/m2)					
≦18.4	12 (13.6%)	4 (18.2%)	6 (20.0%)	3 (37.5%)	0.226
18.5–23.9	58 (65.9%)	16 (72.8%)	21 (70.0%)	4 (50.0%)	
24–27.9	12 (13.6%)	1 (4.5%)	3 (10.0%)	1 (12.5%)	
≧28	6 (6.9%)	1 (4.5%)	0 (0.0%)	0 (0.0%)	
Ca, mmol/L	2.19 (2.09–2.34)	2.25 (2.16–2.31)	2.24 (2.13–2.45)	2.17 (2.08–2.28)	0.485
P, mmol/L	1.72 (1.45–2.14)	1.89 (1.56, 2.47)	1.89 (1.63, 2.24)	1.49 (1.33–2.18)	0.227
PTH, pg/mL	220.50 (111.50–502.50)	369.50 (268.00–715.00)	360.00 (209.00–777.15)	497.50 (249.00–774.50)	0.015*
Hb, g/L	100.00 (91.00–109.00)	103.50 (99.00–109.00)	105.00 (99.00–116.00)	107.00 (94.50–118.50)	0.138
ALB, g/L	38.45 (35.80–40.05)	38.05 (36.80–40.80)	38.25 (34.90–39.60)	36.60 (31.85–39.65)	0.472
sCr, µmol/L	824.00 (634.50–899.50)	853.00 (723.00–973.00)	850.50 (723.00–953.00)	788.50 (458.50–900.00)	0.565
hs-CRP, mg/L	0.90 (0.50–1.50)	1.08 (0.50–2.50)	2.15 (1.20–2.90)	3.93 (3.50–5.05)	<0.001*
HD duration, months	42.00 (20.50–77.50)	38.50 (18.00–83.00)	37.00 (15.00–67.00)	67.00 (59.00–88. 50)	0.119

Notes: P, Hb, ALB, Ca, PTH, sCr, hs-CRP, and HD duration are all non-normal data, articulated by median (range); the rest are conveyed by n (%). Kruskal–Wallis probability between the four groups, with subsequent multiple Mann–Whitney *U* test if significant. Categorical variables are expressed as counts (percentages), unordered categorical variables were tested by chi-square, and ordered categorical variables were tested by Kruskal–Wallis *H*. BMI, body mass index; Ca, serum calcium; P, serum phosphorus; PTH, serum parathyroid hormone; Hb, hemoglobin; ALB, serum albumin; sCr, serum creatinine; hs-CRP, serum hypersensitive C-reactive protein; HD, hemodialysis.*$P < 0.05$.

Degree of skin itching in patients with uremic pruritus as analyzed by single-factor logistic-regression model

Mild itching (OR = 1.936; *P* = 0.009), moderate itching (OR = 2.760, P < 0.001), and severe itching (OR = 7.086; *P* < 0.001) were all related to hs-CRP level. Higher hs-CRP level was closely related to severe itching. PTH was weakly associated with severe itching (OR = 1.001, *P* = 0.033). Hb was slightly associated with moderate itching (OR = 1.036, *P* = 0.026). There was no evidence that P and Place of residence was associated with severity of skin itching, as shown in (Fig 2, S1 Table).

Fig 2. Single-factor logistic-regression analysis of degree of skin itching in patients with uremic pruritus. the error bars represent the lower and upper limits of the 95% confidence interval. the symbol is a circle for mild itching, a triangle for moderate itching, and a square for severe itching. The dashed line in the figure indicates that the odds ratio is equal to 1.

Degree of skin itching in patients with uremic pruritus as analyzed by multivariate logistic regression

We included five variables (hs-CRP, Hb, PTH, P, place of residence) in multiple logistic-regression analysis, using no itching as a reference. The results showed that risk of skin itching increased with level of hs-CRP (mild skin itching, OR = 1.720; 95% CI, 1.044–2.835; *P* = 0.033; moderate skin itching, OR = 2.884; 95% CI, 1.764–4.716; *P* < 0.001; severe skin itching, OR = 8.929; 95% CI, 3.340–23.865; *P* < 0.001) . Compared with urban residents, rural residents have a higher risk of moderate itching (OR = 3.942; 95% CI, 1.105–14.069; *P*=0.035). as shown in (Fig 3, S2 Table).

Fig 3. Multiple logistic-regression analysis of factors influencing skin itching in patients with uremic pruritus. PTH, Hb, hs-CRP were used as covariates, and Age and place of residence were independent variables for regression analysis. the error bars represent the lower and upper limits of the 95% confidence interval. the symbol is a circle for mild itching, a triangle for moderate itching, and a square for severe itching. The dashed line in the figure indicates that the odds ratio is equal to 1.

Discussion

In this study, we found that among 148 eligible patients receiving maintenance HD, 60 had UP (prevalence rate, 40.54%); they included 22 mild cases of skin itching (14.86%), 30 moderate cases (20.27%), and 8 severe cases (5.41%). The proportion of rural residents with severe skin itching was significantly higher than that of urban residents, and there were statistically significant differences in levels of PTH and hs-CRP in patients with different degrees of skin itching. Single-factor logistic-regression analysis discovered a link between hs-CRP levels and all three degrees of itching. Multi-factor logistic regression analysis also indicated that patients with higher hs-CRP levels had higher risks of mild, moderate, and severe skin itching. Interestingly, the results of the multivariate analysis also showed that rural residents have a higher risk of moderate skin itching than urban residents. Therefore, hs-CRP levels were associated with the intensity of skin itching in patients receiving maintenance HD, and higher hs-CRP levels were associated with severe itching.

This study showed that median hs-CRP level gradually increased along with degree of skin itching in patients with uremic pruritus (no itching, 0.90; mild skin itching, 1.08; moderate skin itching, 2.15; severe skin itching, 3.93). Furthermore, hs-CRP levels in patients with moderate and severe skin itching were significantly higher than in those without itching, while hs-CRP levels in patients with severe skin itching were higher than in patients with mild skin itching. These findings suggested that hs-CRP levels might be associated with severity of skin itching. Jiang et al.[11] compared the clinical effects of high-throughput hemodialysis (HTH) and hemodialysis filtration (HDF) treat patients with end-stage nephropathy and explored possible potential mechanisms. Their results showed that levels

of C-reactive protein (CRP) and leukocyte interleukin-6 (IL-6) in patients receiving HTH significantly improved, compared with those in patients who received HDF. The study also found that HTH had an exact therapeutic effect in UP that was related to inhibition of microinflammation in dialysis patients. Mahmudpour's team[12] indicated that the average reduction in VAS scores in the montelukast group was greater than that in the placebo group, and average levels of hs-CRP in this group showed a downward trend; however, average hs-CRP levels in the placebo group gradually increased, showing that the effect of montelukast treatment in UP might be related to a reduction in an inflammatory response. A factor analysis also shows a close correlation between UP and levels of the inflammatory factors CRP and leukocyte IL-6[13]. Based on this study and previous studies, inflammatory responses appear to play an important role in UP.

We analyzed the factors affecting degree of skin itching in UP patients and then corrected for patients' levels of Hb and PTH, place of residence, P. Multi-factor logistic-regression analysis found that hs-CRP levels were associated with severity of skin itching and were risk factors. Compared with patients without itching, the OR value for mild skin itching was 1.720, that for moderate skin itching was 2.884, and that for severe skin itching was 8.929, indicating that degree of skin itching increased along with hs-CRP levels. A previous multivariable logistic-regression analysis confirmed that older age and higher CRP levels are associated with severe itching[3], but in our study, neither single-factor nor multi-factor logistic-regression analysis showed any differences in skin itching between elderly and non-elderly patients. In our study, the OR of patients with severe skin itching was significantly higher than that of patients without itching, suggesting that higher hs-CRP levels were closely associated with severe skin itching, which was consistent with the results of an earlier study. Pakfetrat et al.[14] assessed the effect of sertraline on UP: patients were randomly divided into two groups, the trial group receiving sertraline and the control group taking a placebo, and their severity of itching was measured by two scoring systems (visual-simulation score and DUO scoring system[15]). The researchers found that differences in skin itching intensity between the two scoring systems and between the two groups were directly related to CRP level, and they believed that sertraline reduced skin itching, perhaps due to its effect of reducing the number of inflammatory cytokines. Tseng et al.[16]studying a possible association between vegetarians and UP, found that levels of inflammatory factors such as leukocyte IL-2 and hs-CRP, visual-simulation score (Analog Score and VAS), and itching score were also lower in vegetarian patients than in non-vegetarian ones; this demonstrated that a vegetarian diet might be associated with amelioration of uremic-pruritus severity in hemodialysis patients.

In our study, the composition ratio showed a significant difference between rural and urban patients with different degrees of skin itching. Compared with patients without itching, the proportion of rural patients with moderate and severe skin itching was relatively high, further pairwise comparisons show that compared with mild itching, there is a statistically significant difference in the composition ratio of rural and urban residents in patients with moderate itching. Although the single factor analysis did not indicate that the place of residence was related to the intensity of the patient's skin itching, the multi-factor analysis demonstrated that rural residents had a higher risk of moderate skin itching than urban residents. The possible reason is that on the one hand, compared with urban residents, rural residents have lower living habits and lower health awareness; on the other hand, with the development of urbanization, rural areasthe air pollution in Xinchang's local rural areas is gradually increasing and the urban environment is gradually improving. UP in patients receiving maintenance HD is associated with air pollutants such as NO_2 and CO[17], and a cross-sectional study

suggested that UP in patients receiving maintenance HD might be associated with the number of days in which environmental particulate matter ≤2.5 μ in diameter ($PM_{2.5}$) exceeds standard levels [18]. The above mentioned two studies suggest that itching of the skin might be associated with the patient's living environment. In this study, the ratio of rural residents to urban residents in patients with severe itching was higher than that in patients with moderate itching, but the results of multivariate analysis did not show that rural residents had a higher risk of severe itching than urban residents. The possible reason lies in the patients with severe itching in this study were less relevant, only 8 cases, and 30 patients with moderate itching. therefore, further studies are needed to expand the sample and detect air pollutant levels in urban and rural areas.

PTH levels differed between patients with varying degrees of skin itching. Median PTH level in patients with severe skin itching was significantly higher than that in patients without itching, but there was no statistically significant difference between PTH levels across groups, and single-factor regression analysis suggested a weak association between higher PTH levels and severe skin itching. However, after we corrected for hs-CRP and Hb levels, P, and residence, that association disappeared. After parathyroid excision, PTH level and serum phosphate concentration are significantly reduced, and skin itching disappears[19, 20]. A study by Moldovan et al.[21] suggests that the average value of PTH decreased significantly after parathyroidectomy, and the symptoms of bone pain were significantly improved. However, muscle weakness and itching of the skin did not improve. Levy et al.[22] studied the associated symptoms of sHPT in patients receiving maintenance HD. Their results showed that an increase in PTH levels was associated with skin itching worsening over time, and the effect was more obvious with larger changes in PTH level. However, the combination of the disease, detection of medication, inclusion of up to 19 symptoms, the larger *P*-value threshold (0.1), and other promiscuous factors might have affected the reliability of that study.

Other studies suggested that UP is related to Ca, P, ALB, and uremic toxins[14, 23]. Our research showed that compared with patients without pruritus, the levels of Ca, P, ALB, and sCr in pruritus patients were not statistically abnormal. ALB, and sCr levels were not associated with UP.

Overall, our study suggested that the intensity of skin itching in patients with UP was associated with hs-CRP and that higher hs-CRP levels were associated with severe skin itching. The limitation of this study is that it was a single-center, cross-sectional study that did not show a causal relationship between severity of skin itching in UP and hs-CRP, so further validation is needed from multi-center, large-sample, prospective cohort studies.

Supporting information

S1 Fig. Comparison of patients with varying degrees of skin itching and varying hs-CRP and PTH levels. (A) Intergroup comparison of hs-CRP levels; **(B)** intergroup comparison of PTH levels; **(C)** intergroup comparison of the composition ratio of rural to urban residents. ****$P < 0.0001$, **$P < 0.01$. Error line represents minimum and maximum values.

S1 Table. Single-factor logistic-regression analysis of degree of skin itching in patients with uremic pruritus.

S2 Table. Multiple logistic-regression analysis of factors influencing skin itching in patients with uremic pruritus, using hs-CRP as a variable.

Acknowledgements

We thank LetPub (www.letpub.com) for its linguistic assistance during the preparation of this manuscript.

Author contributions

Conceptualization: Jian-Hui Zhao, Yi-Wen Li.
Data curation: Qing-Shuang Zhu, Li-Li Wang.
Investigation: Jian-Hui Zhao, Yi-Wen Li, Qing-Shuang Zhu.
Methodology: Jian-Hui Zhao, Yi-Wen Li.
Project administration: Jian-Hui Zhao.
Supervision: Yi-Wen Li.
Writing – original draft: Jian-Hui Zhao.
Writing – review and editing: Jian-Hui Zhao, Yi-Wen Li, Qing-Shuang Zhu.

Funding: The authors received no specific funding for this work.

References

1. Kfoury LW, Jurdi MA. Uremic pruritus. J Nephrol. 2012;25(5):644-52. Epub 2011/10/11. doi: 10.5301/jn.5000039. PubMed PMID: 21983988.

2. Combs SA, Teixeira JP, Germain MJ. Pruritus in Kidney Disease. Semin Nephrol. 2015;35(4):383-91. Epub 2015/09/12. doi: 10.1016/j.semnephrol.2015.06.009. PubMed PMID: 26355256; PubMed Central PMCID: PMCPMC5497472.

3. Rayner HC, Larkina M, Wang M, Graham-Brown M, van der Veer SN, Ecder T, et al. International Comparisons of Prevalence, Awareness, and Treatment of Pruritus in People on Hemodialysis. Clin J Am Soc Nephrol. 2017;12(12):2000-7. Epub 2017/09/20. doi: 10.2215/CJN.03280317. PubMed PMID: 28923831; PubMed Central PMCID: PMCPMC5718267.

4. Grochulska K, Ofenloch RF, Mettang T, Weisshaar E. Mortality of Haemodialysis Patients With and Without Chronic Itch: A Follow-up Study of the German Epidemiological Hemodialysis Itch Study (GEHIS). Acta Derm Venereol. 2019;99(4):423-8. Epub 2019/01/24. doi: 10.2340/00015555-3125. PubMed PMID: 30673111.

5. Wu HY, Peng YS, Chen HY, Tsai WC, Yang JY, Hsu SP, et al. A Comparison of Uremic Pruritus in Patients Receiving Peritoneal Dialysis and Hemodialysis. Medicine (Baltimore). 2016;95(9):e2935. Epub 2016/03/06. doi: 10.1097/md.0000000000002935. PubMed PMID:

26945400; PubMed Central PMCID: PMCPMC4782884.

6. Wieczorek A, Krajewski P, Kozioł-Gałczyńska M, Szepietowski JC. Opioid receptors expression in the skin of haemodialysis patients suffering from uraemic pruritus. J Eur Acad Dermatol Venereol. 2020. Epub 2020/03/15. doi: 10.1111/jdv.16360. PubMed PMID: 32170896.

7. Abdelsalam M, Tawfik M, Reda EM, Eldeeb AA, Abdelwahab A, Zaki ME, et al. Insulin Resistance and Hepatitis C Virus-Associated Subclinical Inflammation Are Hidden Causes of Pruritus in Egyptian Hemodialysis Patients: A Multicenter Prospective Observational Study. Nephron. 2019;143(2):120-7. Epub 2019/07/17. doi: 10.1159/000501409. PubMed PMID: 31311030.

8. Aliasgharpour M, Zabolypour S, Asadinoghabi A, Haghani H, Lesanpezeshki M. The Effect of Increasing Blood Flow Rate on Severity of Uremic Pruritus in Hemodialysis Patients: A Single Clinical Trial. J Natl Med Assoc. 2018;110(3):270-5. Epub 2018/05/21. doi: 10.1016/j.jnma.2017.04.008. PubMed PMID: 29778130.

9. Elman S, Hynan LS, Gabriel V, Mayo MJ. The 5-D itch scale: a new measure of pruritus. Br J Dermatol. 2010;162(3):587-93. Epub 2009/12/10. doi: 10.1111/j.1365-2133.2009.09586.x. PubMed PMID: 19995367; PubMed Central PMCID: PMCPMC2875190.

10. Keithi-Reddy SR, Patel TV, Armstrong AW, Singh AK. Uremic pruritus. Kidney Int. 2007;72(3):373-7. Epub 2007/04/13. doi: 10.1038/sj.ki.5002197. PubMed PMID: 17429345.

11. Jiang X, Ji F, Chen ZW, Huang QL. Comparison of high-flux hemodialysis with hemodialysis filtration in treatment of uraemic pruritus: a randomized controlled trial. Int Urol Nephrol. 2016;48(9):1533-41. Epub 2016/07/06. doi: 10.1007/s11255-016-1364-2. PubMed

PMID: 27379625.

12. Mahmudpour M, Roozbeh J, Raiss Jalali GA, Pakfetrat M, Ezzat Zadegan S, Sagheb MM. Therapeutic Effect of Montelukast for Treatment of Uremic Pruritus in Hemodialysis Patients. Iran J Kidney Dis. 2017;11(1):50-5. Epub 2017/02/09. PubMed PMID: 28174353.

13. Schricker S, Heider T, Schanz M, Dippon J, Alscher MD, Weiss H, et al. Strong Associations Between Inflammation, Pruritus and Mental Health in Dialysis Patients. Acta Derm Venereol. 2019;99(6):524-9. Epub 2019/01/24. doi: 10.2340/00015555-3128. PubMed PMID: 30673107.

14. Pakfetrat M, Malekmakan L, Hashemi N, Tadayon T. Sertraline can reduce uremic pruritus in hemodialysis patient: A double blind randomized clinical trial from Southern Iran. Hemodial Int. 2018;22(1):103-9. Epub 2017/03/07. doi: 10.1111/hdi.12540. PubMed PMID: 28263039.

15. Duo LJ. Electrical needle therapy of uremic pruritus. Nephron. 1987;47(3):179-83. Epub 1987/01/01. doi: 10.1159/000184487. PubMed PMID: 3500424.

16. Tseng CY, Wu TT, Lai CW, Lin HJ, Chou CY, Chang CT, et al. Vegetarian diet may ameliorate uremic pruritus in hemodialysis patients. Ren Fail. 2018;40(1):514-9. Epub 2018/11/07. doi: 10.1080/0886022X.2018.1512871. PubMed PMID: 30396306; PubMed Central PMCID: PMCPMC7012008.

17. Huang W-H, Lin J-H, Weng C-H, Hsu C-W, Yen T-H. Environmental NO 2 and CO Exposure: Ignored Factors Associated with Uremic Pruritus in Patients Undergoing Hemodialysis. Scientific reports. 2016;6(1):1-10.

18. Liu MH, Chan MJ, Hsu CW, Weng CH, Yen TH, Huang WH. Association of uremic

pruritus in hemodialysis patients with the number of days of high mean 24-hour particulate matter with a diameter of <2.5 mum. Ther Clin Risk Manag. 2017;13:255-62. Epub 2017/03/07. doi: 10.2147/TCRM.S129133. PubMed PMID: 28260912; PubMed Central PMCID: PMCPMC5328135.

19. Jing Y, Zhao H, Ge Y, Jia F, He Q, Wang S, et al. Application of total parathyroidectomy with auto-transplantation for uremia secondary hyperparathyroidism treatment. International journal of clinical and experimental medicine. 2015;8(7):11188.

20. Zhang Y, Lu Y, Feng S, Zhan Z, Shen H. Evaluation of laboratory parameters and symptoms after parathyroidectomy in dialysis patients with secondary hyperparathyroidism. Ren Fail. 2019;41(1):921-9. Epub 2019/10/02. doi: 10.1080/0886022X.2019.1666724. PubMed PMID: 31573378; PubMed Central PMCID: PMCPMC6781481.

21. Moldovan D, Racasan S, Kacso IM, Rusu C, Potra A, Bondor C, et al. Survival after parathyroidectomy in chronic hemodialysis patients with severe secondary hyperparathyroidism. Int Urol Nephrol. 2015;47(11):1871-7. Epub 2015/09/18. doi: 10.1007/s11255-015-1106-x. PubMed PMID: 26377492.

22. Levy AR, Xing S, Brunelli SM, Cooper K, Finkelstein FO, Germain MJ, et al. Symptoms of Secondary Hyperparathyroidism in Patients Receiving Maintenance Hemodialysis: A Prospective Cohort Study. American Journal of Kidney Diseases. 2020;75(3):373-83.

23. Momose A, Shiraiwa Y, Narita S, Kusumi T, Goto S, Sera K. Total Calcium and Albumin Are Decreased in the Deeper Epidermis of Patients with Chronic Kidney Disease-Associated Pruritus. Nephron. 2017;136(2):103-10. Epub 2017/02/10. doi: 10.1159/000458417. PubMed PMID: 28183080.

Figure

Click here to access/download;Figure;Fig1.tiff

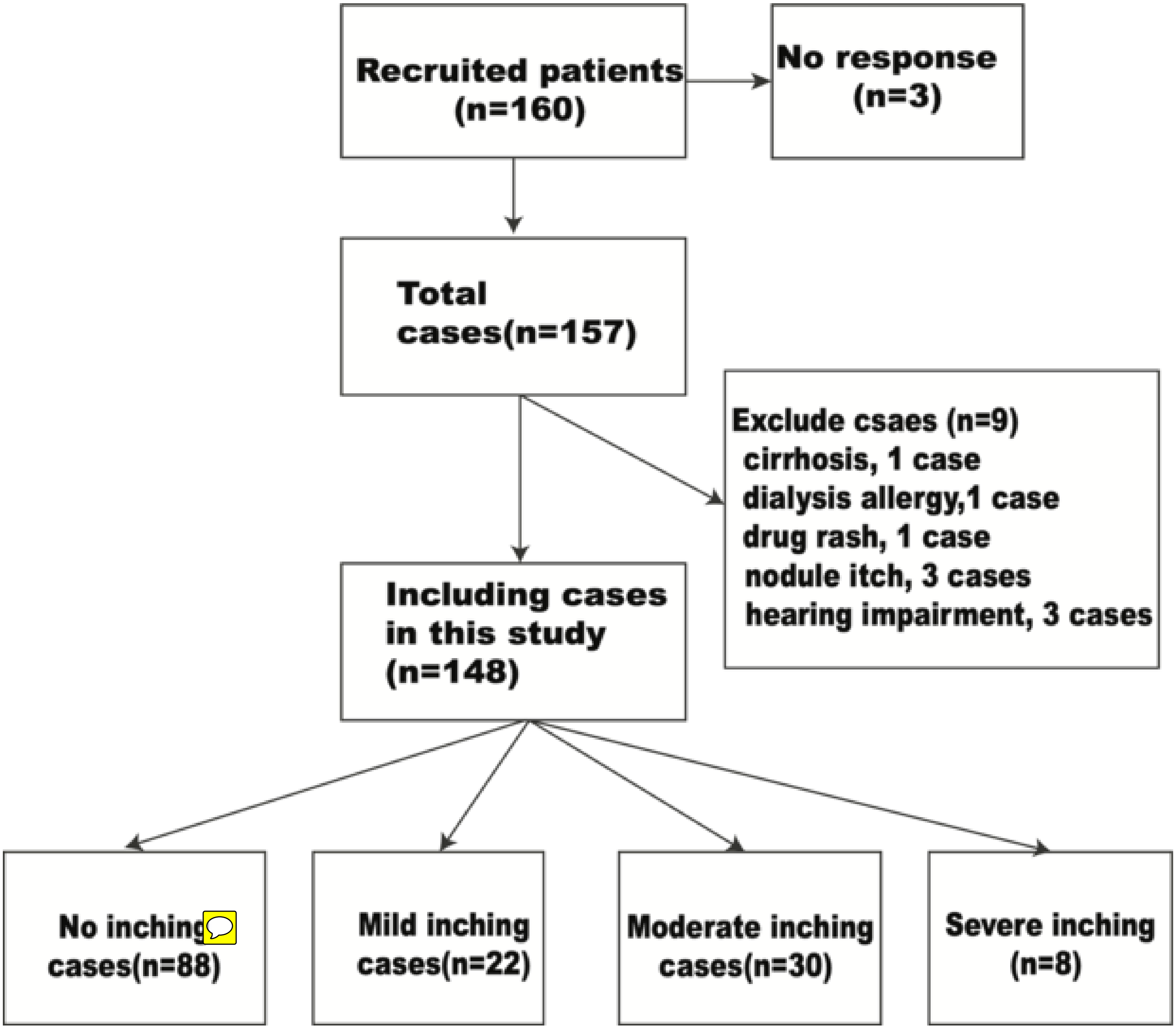

Figure

Click here to access/download;Figure;Fig 2.tiff

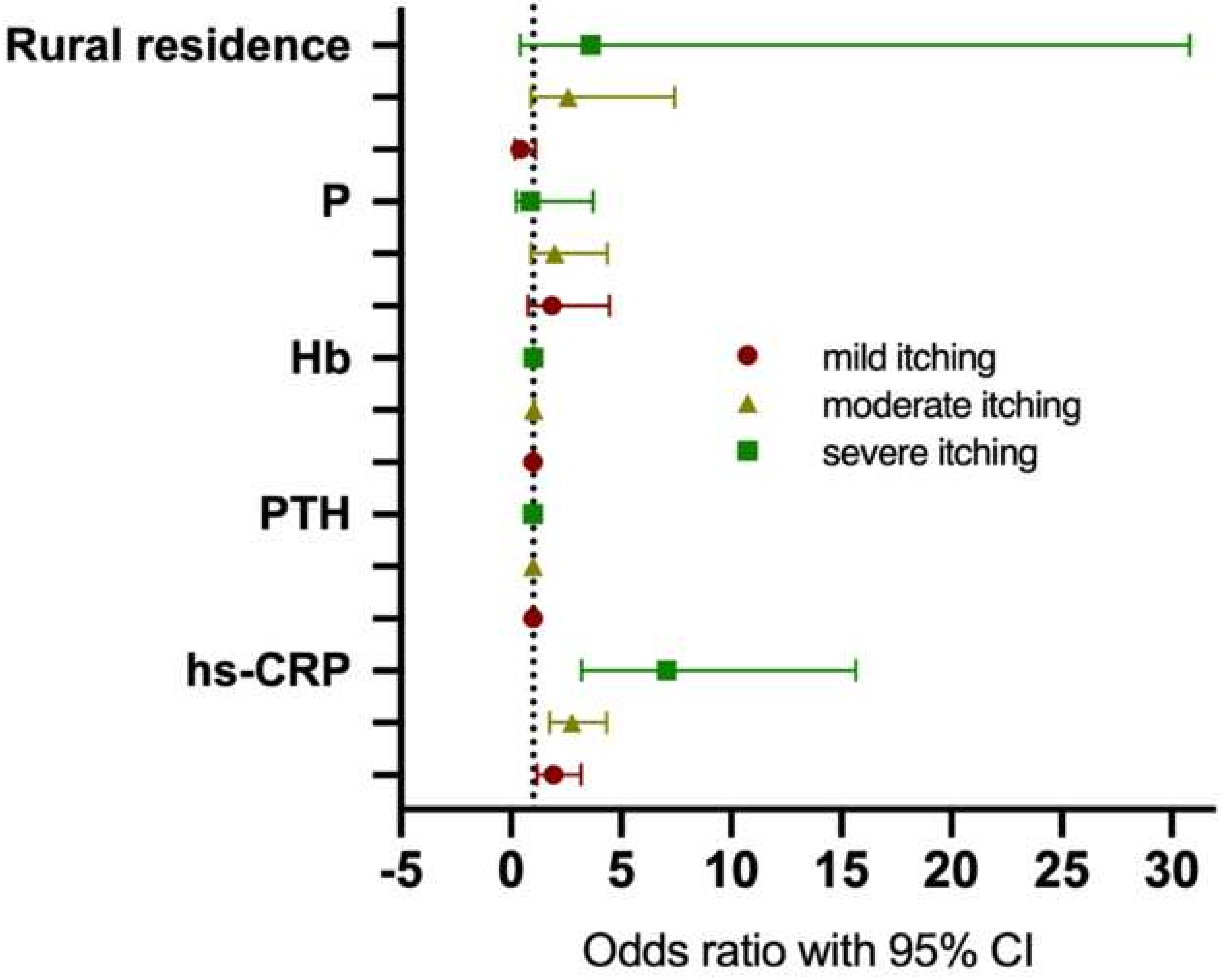

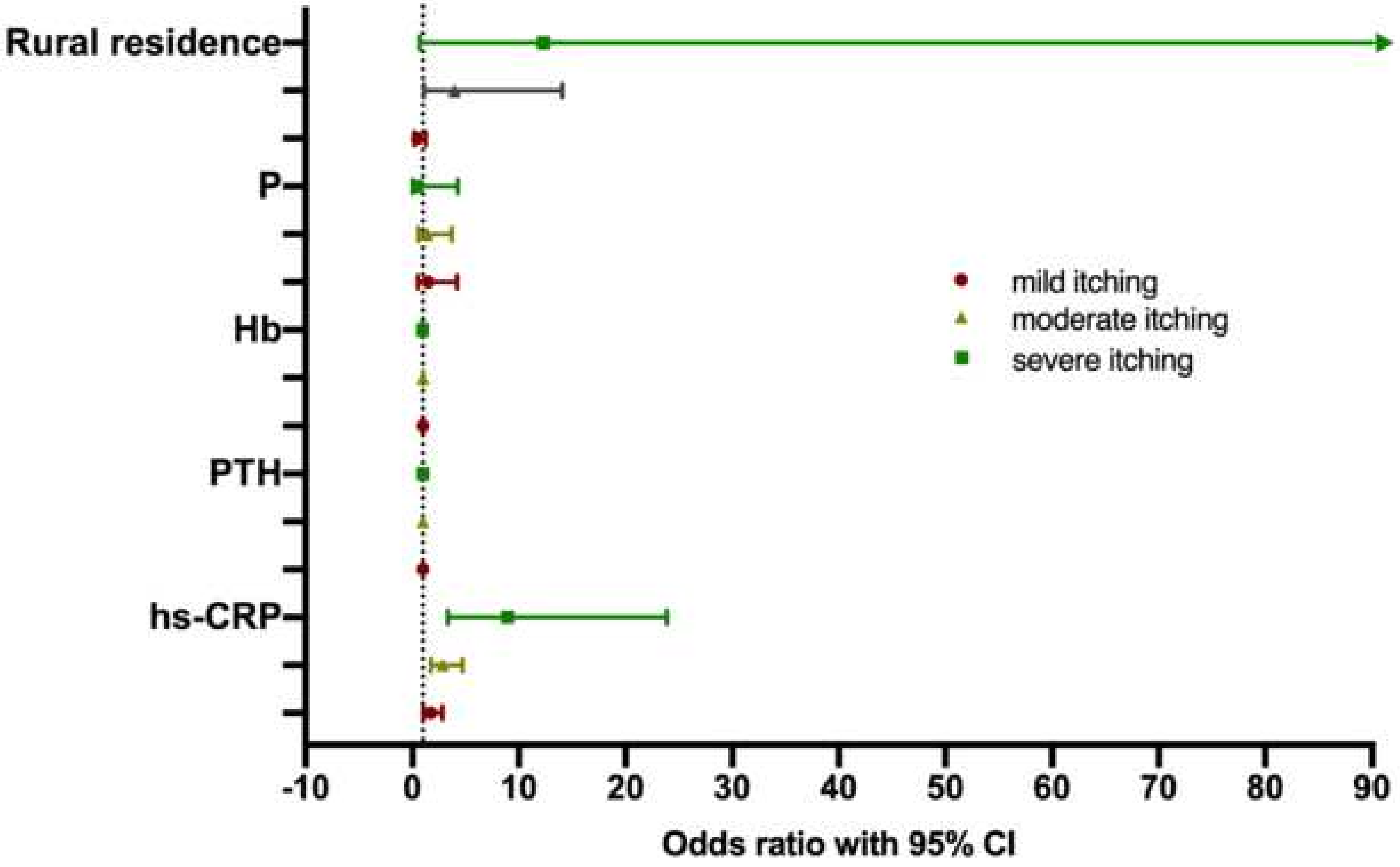
Rural residence
P
Hb
PTH
hs-CRP
-10
0
10
20
30
40
50
60
70
80
90
Odds ratio with 95% CI
mild itching
moderate itching
severe itching

Click here to access/download
Supporting Information - Compressed/ZIP File Archive
Compressed_ZIP File Archive.zip
