# Supplementary material for: Determinants of the intensity of uremic pruritus in patients receiving maintenance hemodialysis: A cross-sectional study
Source: PLoS One. 2021 Jan 20;16(1):e0245370. doi: 10.1371/journal.pone.0245370 (PMC7817000; doi:10.1371/journal.pone.0245370)
Supplement: Attachment — Submitted filename: PONE-D-20-18098_R1 minor observations.pdf [file pone.0245370.s005.pdf]

PLOS ONE

Determinants of the intensity of uremic pruritus in patients receiving maintenance hemodialysis: A cross-sectional study

--Manuscript Draft--

**Manuscript Number:**	PONE-D-20-18098R1
**Article Type:**	Research Article
**Full Title:**	Determinants of the intensity of uremic pruritus in patients receiving maintenance hemodialysis: A cross-sectional study
**Short Title:**	Determinants of the intensity of uremic pruritus in Hemodialysis patients
**Corresponding Author:**	Jian-Hui Zhao, M.M.<br>Xinching Country People's Hospital<br>CHINA
**Keywords:**	high sensitivity C-reactive protein; uremic pruritus; the intensity of skin itching; maintenance hemodialysis
**Abstract:**	Background : Uremic pruritus (UP) is a common and frustrating symptom in patients receiving hemodialysis (HD). This study aimed to investigate the determinants of the intensity of UP in patients receiving maintenance HD.<br>Methods : A cross-sectional study on uremic pruritus in male and female patients receiving HD was conducted in September 2019.This study included 148 eligible patients who received HD at the Blood Purification Center of Xinchang County People's Hospital, Zhejiang Province, China from March 2019 to June 2019. We collected general data such as age, sex, body mass index (BMI), place of residence, educational level, diabetes mellitus status and duration of HD; as well as clinical biochemical indicators, including serum calcium (Ca), serum phosphorus (P), serum albumin (ALB), hemoglobin (Hb), serum parathyroid hormone (PTH), pre-dialysis serum urea nitrogen (BUN), urea nitrogen clearance index (KT/V), ferritin (FER) and pre-dialysis serum creatinine (sCr). We also assayed the inflammatory cytokine serum high sensitivity C-reactive protein (hs-CRP). The Five-Dimensional Itching Scale (5DIS) was used to evaluate the degree of skin itching (none, mild, moderate, or severe). We used multiple logistic regression to analyze influencing factors on the degree of skin itching in patients with UP.<br>Results : (1) Of the 148 patients, 60 had uremic pruritus (incidence rate, 40.54%). These included 22 cases of mild skin itching (14.86%), 30 of moderate skin itching (20.27%), and 8 of severe skin itching (5.41%). (2) Compared with uremia patients without skin pruritus, patients with UP had higher levels of PTH, Hb, BUN, and hs-CRP. (3) The composition ratio showed significant differences between urban and rural patients with different degrees of skin itching (P = 0.017); moreover, the difference of PTH and hs-CRP levels was statistically significant (P = 0.009 and < 0.001, respectively). (4)Using no itching as a reference, multiple logistic-regression analysis showed that as hs-CRP level increased, the patient's risks of mild skin itching (odds ratio [OR] = 1.740; 95% confidence interval [CI], 1.061–2.854; P = 0.028), moderate skin itching (OR = 2.8838 95% CI, 1.744–4.718; P < 0.001), and severe skin itching (OR = 9.440; 95% CI, 3.547–25.124; P < 0.001) all increased as well. Compared with urban residents, rural residents have a higher risk of moderate itching (OR = 3.869; 95% CI, 1.099–13.622; P=0.035).<br>Conclusion : Levels of hs-CRP were associated with degree of skin itching in patients with UP. Higher hs-CRP levels were closely related to severe skin itching.
**Order of Authors:**	Jian-Hui Zhao, M.M.
	Qiu-Shuang Zhu
	Yi-Wen Li
	Li-Li Wang
**Response to Reviewers:**	Response to the PLOS ONE's editor<br><br>Thank you for the hard work of the academic editors of "PLOS ONE". Thank you for accompanying me and helping me grow up. As for the issues that need my attention, I

Powered by Editorial Manager® and ProduXion Manager® from Aries Systems Corporation

summarize as follows.

1. Please ensure that your manuscript meets PLOS ONE's style requirements, including those for file naming. The PLOS ONE style templates can be found at https://journals.plos.org/plosone/s/file?id=wjVg/PLOSOne_formatting_sample_main_body.pdf and https://journals.plos.org/plosone/s/file?id=ba62/PLOSOne_formatting_sample_title_authors_affiliations.pdf

Response：
We have revised the paper following your journal's formatting requirements for title, authorship, and text.

2.While revising your submission, please upload your figure files to the Preflight Analysis and Conversion Engine (PACE) digital diagnostic tool, https://pacev2.apexcovantage.com/. PACE helps ensure that figures meet PLOS requirements. To use PACE, you must first register as a user. Registration is free. Then, login and navigate to the UPLOAD tab, where you will find detailed instructions on how to use the tool. If you encounter any issues or have any questions when using PACE, please email PLOS at figures@plos.org. Please note that Supporting Information files do not need this step.
Response：
A total of four images are in the paper, and the image files have been uploaded to the PACE software, all with a resolution of 600ppi, 2000-4500 pixels wide and 1358-3925 pixels high, and are currently sealed in the PACE software.

Response to Reviewer

Thank the reviewers for these lovely comments concerning our manuscript entitled “Determinants of the intensity of uremic pruritus in patients receiving maintenance hemodialysis: A cross-sectional study”. These comments are all valuable and very helpful for revising and improving our paper, as well as the essential guiding significance to our researches. We have studied comments carefully and have made corrections which we hope to meet with approval. The response to the reviewer’s comments are as follows:

Reviewer #1
The authors performed a cross-sectional analysis of 148 hemodialysis patients to identify factors associated with uremic pruritus. They found that higher levels of PTH, hemoglobin, and hs-CRP, as well as the rural residence of patients were associated with worse pruritus. There are major concerns with the lack of dialysis and other pertinent laboratory data. Overall, the results do not add new data to the existing literature.

1. There is no data provided for dialysis adequacy, which has been shown to be associated with pruritus in prior studies. Likewise, BUN levels, nPCR, ferritin levels, and diabetic status should be included in the analysis.
Response:
The revision adds these factors, such as urea nitrogen, ferritin level, diabetes, and single pool KT/V(spKT/V), to the list of factors related to hemodialysis. Normalized protein catabolic rate (nPCR) is a nutritional index for hemodialysis patients. Its measurement requires multiple blood collections, which can be challenging to obtain, especially with the inclusion of serum albumin, which is a nutritional index. The spKT/V index reflects the adequacy of single hemodialysis.

This section is mainly shown on pages 6-11, tables 1-3.

Powered by Editorial Manager® and ProduXion Manager® from Aries Systems Corporation

2. The authors used the Five-Dimensional Itching Scale (5DIS) to assess the degree of pruritus in patients, noting that this scale would “produce more objective and comprehensive results” compared to the visual-analogue scoring scale. The authors should provide data to justify this statement.
Response:
In this study, we used a five-dimensional itching scale to evaluate skin itching across multiple dimensions in uremic patients undergoing maintenance HD. Although VAS is sufficient to assess the severity of symptoms, it does not take into account other aspects of pruritus, such as the relative impact of pruritus on quality of life, changes in pruritus over time, and the location of pruritus. The 5-D score was strongly correlated with the numerical rating scale（NRS）and VAS[5, 26], which is a questionnaire with acceptable validity, reliability and sensitivity to change to evaluate pruritus in Thai and Arabic patients[26, 27]. The 5-dimensional Pruritus Scale is recommended to assess the severity of pruritus because it is more accurate than VAS and more sensitive to the multidimensional nature of pruritus[28].

This section is detailed on page15, line 29-38.

3. A large proportion of the discussion was dedicated to the finding that higher hs-CRP levels were associated with pruritus. A prior study has previously reported this finding, along with levels of cytokines and T cells (PMID 16249205); this reference should be cited.
Response：
This section is detailed on Reference #8（page 13, line35）

4. The number of patients with severe pruritus is small (n=8) and therefore, meaningful associations with severe pruritus may not be appreciated.
Response：
In this study, there were a total of 148 patients with uremia and eight patients with severe skin pruritus, accounting for 5.4% of the total patients. 2012-2015 DPPSO study[3] surveyed 7,629 people, divided into five groups (not at all, somewhat, moderately, Very Much, Extremely) according to the degree of itchy skin. Extreme Itching accounted for 7%, which is slightly higher than 5.4% of patients with severe Itching in this study. The reason for the differences may lie in the different criteria used to assess the degree of pruritus in the patient's skin.

This section is detailed on page 14, lines 37-43

Reviewer #2
In this study, the authors studied uremic pruritus using the five-dimensional itch scale in 148 adult patients of end-stage renal disease receiving hemodialysis and report the prevalence of 40.5% and association with parathormone (PTH), hemoglobin and high-sensitive C reactive protein (hs-CRP) on bivariate analysis and hs-CRP alone on multiple logistic regression. The study seems to be designed logically, conducted scientifically and analyzed with due statistical rigour. However, the manuscript is lengthy, does not have a smooth flow of information and requires rewriting as per the STROBE checklist. Major observations are described below. Minor observations are inserted in the uploaded version of the manuscript as “sticky notes” at appropriate places.
Response:
1）We have rewritten the paper according to the STROBE checklist, details of which can be found in the attached STROBE checklist.
2）Concerning some minor observation, we have followed your comments with deep appreciation.

Major observation:

1. The introduction should briefly summarize the present state of knowledge on uremic pruritus, highlight the gap in understanding the study sought to address and state the study’s objective. Comparison with previous studies (measurement of severity of Itching) should be reserved for discussion.
Response:
The reviewer's suggestions were excellent, and we have corrected them.

Powered by Editorial Manager® and ProduXion Manager® from Aries Systems Corporation

This section has been moved to page 15, lines 23-29.

2. The stated aims of the study are to “1. explore the prevalence of UP in maintenance HD patients and the demographic and clinical characteristics of pruritus patients. Explore determinants of higher pruritus intensity based on participants’ clinical and biochemical parameters and their pruritus assessments by 5DIS at baseline”. The second aim is somewhat unclear. Were characteristics of patients with higher pruritus intensity to be compared with those of patients with of lower pruritus intensity or with those of patients without pruritus? Clear statement in the PICO format is suggested. What is the novelty of the research question? This needs to be justified.
Response:
objectives
(1). explore the prevalence of UP in maintenance HD patients and the demographic and clinical characteristics of pruritus patients.
(2). analyze the relationship between high-sensitivity C-reactive protein and different degrees of skin itching.
This section has been moved to page 2, lines 28-32.

3. The setting of the study may be described in “Methods”.
Response:

This section is detailed on Methods (study setting), page2, lines 38 & page3, lines 1-5.

4.How were biochemical parameters such as serum calcium, phosphorus, creatinine, etc assayed? The detailed methods need not be described but should be referenced.
Response:
We assayed biochemical indicators such as Ca, P, iPTH, Hb, ALB, sCr, Bun, FER, sp/KTV and hs-CRP. The hospital's central laboratory performed all of the laboratory tests, and auto-analyzers were used to determine biochemical data, iPTH was measured with Roche second-generation assay. The clinical biochemical indicators are based on the data within three months from the survey day. If there is no data within three months, the patient is required to re-test. If the patient is unwilling to cooperate with the test, it is a missing value. spKT/V (single pool KT/V) =-ln(R-0.008t)+(4-3.5R)*(ΔBW/BW). Note: K refers to Blood urea clearance rate of dialyzer (L/h) , t is the dialysis time (h), and V is the distribution volume of urea (V). R is the ratio of blood urea nitrogen after dialysis to blood urea nitrogen before dialysis; t is single dialysis time in h; ΔBW is the weight change value before and after dialysis, i.e. ultrafiltration, unit L; BW is weight in kg. Blood collection requirements: blood samples before dialysis from the artery end of the vascular path, after dialysis before blood sample collection to stop ultrafiltration, reduce blood flow of 50 ml/min, wait 15 seconds after blood collection from the artery as a blood sample after dialysis.

This section is detailed on page 3, lines 36-41 & page 4, lines 1-7.

5.The study reports no significant association of uremic pruritus with factors such as calcium, phosphorus, creatinine and albumin. However, the sample size calculation was made to estimate the prevalence of uremic pruritus. Could the study have been under powered for the study of these factors?
Response:
Some studies suggested that UP is related to Ca, P, ALB, and uremic toxins[11, 20]. Nevertheless, contradictory data on the impact of Ca and P have been reported[21]. In this study, no statistically significant differences were found in Ca, P, ALB, and creatinine when comparing groups of patients with different degrees of skin pruritus. There may indeed be a lack of test power (Since the data for these factors are non-normally distributed, the exact distribution is unknown. Based on statistical theory, it is not possible to calculate the true test power with certainty.) , which is considered to be related to the small sample size of this study and the unbalanced sample size among the groups. However, single-factor logistic regression analysis also did not find an association between these factors and the degree of skin pruritus. Therefore, these factors were ultimately not included in the multifactor logistic regression analysis. In fact, despite various substances have been discussed as potential pruritogens in chronic renal diseases, a causal relationship could never be established[22].

Powered by Editorial Manager® and ProduXion Manager® from Aries Systems Corporation

	This section is detailed on page 15, lines 14-22.<br><br>6.The following factors influencing uremic pruritus have not been studied: diet, insulin resistance, dialysis adequacy, interleukins 6 & 2, and κ opioid receptor distribution. These need to be acknowledged as limitations.<br>Response:<br>Firstly, this study was a single-center study with a limited number of people on hemodialysis, and therefore the number of people included in the study was limited. Many factors influence skin itching in uremic patients, and it is not practical to include all relevant aspects of the study. Secondly, there is the issue of patient compliance. For example, to study diet factor, we need to distinguish between vegetarian and non-vegan patients, which requires strict control of the patient's diet. For insulin resistance, if the HOMA model is used to assess the resistance of the body to insulin (HOMA-IR), fasting blood glucose and fasting insulin levels need to be measured based on the formula HOMA-IR=FPG×FINS/22.5, therefor, fasting blood needs to be collected before hemodialysis. Some patients are difficult to cooperate, the missing value of the study will be further increased. Thirdly, for dialysis adequacy, we included a urea clearance index (kt/v) to assess this. In conclusion, it is essential to acknowledge the limitations of this study.<br><br>This section is detailed on page 13, lines 17-19.
**Additional Information:**	
**Question**	**Response**
**Financial Disclosure**<br><br>Enter a financial disclosure statement that describes the sources of funding for the work included in this submission. Review the submission guidelines for detailed requirements. View published research articles from *PLOS ONE* for specific examples.<br><br>This statement is required for submission and **will appear in the published article** if the submission is accepted. Please make sure it is accurate.	The authors received no specific funding for this work.

**Unfunded studies**<br>Enter: *The author(s) received no specific funding for this work.*<br><br>**Funded studies**<br>Enter a statement with the following details:<br>• Initials of the authors who received each award<br>• Grant numbers awarded to each author<br>• The full name of each funder<br>• URL of each funder website<br>• Did the sponsors or funders play any role in the study design, data collection and analysis, decision to publish, or preparation of the manuscript?<br>• **NO** - Include this sentence at the end of your statement: *The funders had no role in study design, data collection and analysis, decision to publish, or preparation of the manuscript.*<br>• **YES** - Specify the role(s) played.<br><br>* typeset	
**Competing Interests**<br><br>Use the instructions below to enter a competing interest statement for this submission. On behalf of all authors, disclose any competing interests that could be perceived to bias this work—acknowledging all financial support and any other relevant financial or non-financial competing interests.<br><br>This statement **will appear in the published article** if the submission is accepted. Please make sure it is accurate. View published research articles from *PLOS ONE* for specific examples.	The authors have declared that no competing interests exist.

<table>
<tr><td>NO authors have competing interests

Enter: The authors have declared that no competing interests exist.

Authors with competing interests

Enter competing interest details beginning with this statement:

I have read the journal's policy and the authors of this manuscript have the following competing interests: [insert competing interests here]

* typeset</td><td></td></tr>
<tr><td>Ethics Statement

Enter an ethics statement for this submission. This statement is required if the study involved:

• Human participants
• Human specimens or tissue
• Vertebrate animals or cephalopods
• Vertebrate embryos or tissues
• Field research

Write "N/A" if the submission does not require an ethics statement.

General guidance is provided below. Consult the submission guidelines for detailed instructions. Make sure that all information entered here is included in the Methods section of the manuscript.</td><td>name of institutional committee: XinChang County People's Hospital Ethics Committee.
approval number: XCXRMYY2018-001.
Written consent.</td></tr>
</table>

Powered by Editorial Manager® and ProduXion Manager® from Aries Systems Corporation

**Format for specific study types**<br><br>**Human Subject Research (involving human participants and/or tissue)**<br>• Give the name of the institutional review board or ethics committee that approved the study<br>• Include the approval number and/or a statement indicating approval of this research<br>• Indicate the form of consent obtained (written/oral) or the reason that consent was not obtained (e.g. the data were analyzed anonymously)<br><br>**Animal Research (involving vertebrate animals, embryos or tissues)**<br>• Provide the name of the Institutional Animal Care and Use Committee (IACUC) or other relevant ethics board that reviewed the study protocol, and indicate whether they approved this research or granted a formal waiver of ethical approval<br>• Include an approval number if one was obtained<br>• If the study involved *non-human primates*, add *additional details* about animal welfare and steps taken to ameliorate suffering<br>• If anesthesia, euthanasia, or any kind of animal sacrifice is part of the study, include briefly which substances and/or methods were applied<br><br>**Field Research**<br><br>Include the following details if this study involves the collection of plant, animal, or other materials from a natural setting:<br>• Field permit number<br>• Name of the institution or relevant body that granted permission	
**Data Availability**<br><br>Authors are required to make all data underlying the findings described fully available, without restriction, and from the time of publication. PLOS allows rare exceptions to address legal and ethical concerns. See the PLOS Data Policy and FAQ for detailed information.	Yes - all data are fully available without restriction

Powered by Editorial Manager® and ProduXion Manager® from Aries Systems Corporation

A Data Availability Statement describing where the data can be found is required at submission. Your answers to this question constitute the Data Availability Statement and **will be published in the article**, if accepted.<br><br>**Important:** Stating 'data available on request from the author' is not sufficient. If your data are only available upon request, select 'No' for the first question and explain your exceptional situation in the text box.<br><br>Do the authors confirm that all data underlying the findings described in their manuscript are fully available without restriction?	
**Describe where the data may be found in full sentences. If you are copying our sample text, replace any instances of XXX with the appropriate details.**<br><br>• If the data are **held or will be held in a public repository**, include URLs, accession numbers or DOIs. If this information will only be available after acceptance, indicate this by ticking the box below. For example: *All XXX files are available from the XXX database (accession number(s) XXX, XXX.*).<br>• If the data are all contained **within the manuscript and/or Supporting Information files**, enter the following: *All relevant data are within the manuscript and its Supporting Information files.*<br>• If neither of these applies but you are able to provide **details of access elsewhere**, with or without limitations, please do so. For example:<br><br>*Data cannot be shared publicly because of [XXX]. Data are available from the XXX Institutional Data Access / Ethics Committee (contact via XXX) for researchers who meet the criteria for access to confidential data.*<br><br>*The data underlying the results presented in the study are available from (include the name of the third party*	All relevant data are within the manuscript and its Supporting Information files.

and contact information or URL).<br>• This text is appropriate if the data are owned by a third party and authors do not have permission to share the data.<br><br>* typeset	
Additional data availability information:	Tick here if the URLs/accession numbers/DOIs will be available only after acceptance of the manuscript for publication so that we can ensure their inclusion before publication.

Powered by Editorial Manager® and ProduXion Manager® from Aries Systems Corporation

Manuscript Click here to access/download;Manuscript;Revised Manuscript.docx

Determinants of the intensity of uremic pruritus in patients receiving maintenance hemodialysis: A cross-sectional study

Jian-Hui Zhao[1,2*] , Qiu-Shuang Zhu [1,2] , Yi-Wen Li[3] , Li-Li Wang[2]

1 Department of Nephrology, Xinchang County People's Hospital, Xinchang, Shaoxing, Zhejiang Province, China

2 Blood Purification Center, Xinchang County People's Hospital, Xinchang, Shaoxing, Zhejiang Province, China

3 Department of Nephrology, Zhejiang People's Hospital, Hangzhou, Zhejiang Province, China

* Corresponding author

E-mail: lyzhaojh@126.com (Zhao, J.H.)

Abstract

Background: Uremic pruritus (UP) is a common and frustrating symptom in patients receiving hemodialysis (HD). This study aimed to investigate the determinants of the intensity of UP in patients receiving maintenance HD.

Methods: A cross-sectional study on uremic pruritus in male and female patients receiving HD was conducted in September 2019.This study included 148 eligible patients who received HD at the Blood Purification Center of Xinchang County People's Hospital, Zhejiang Province, China from March 2019 to June 2019. We collected general data such as age, sex, body mass index (BMI), place of residence, educational level, diabetes mellitus status and duration of HD; as well as clinical biochemical indicators, including serum calcium (Ca), serum phosphorus (P), serum albumin (ALB), hemoglobin (Hb), serum parathyroid hormone (PTH), pre-dialysis serum urea nitrogen（BUN), urea nitrogen clearance index (KT/V), ferritin (FER) and pre-dialysis serum creatinine (sCr). We also assayed the inflammatory cytokine serum high sensitivity C-reactive protein (hs-CRP). The Five-Dimensional Itching Scale (5DIS) was used to evaluate the degree of skin itching (none, mild, moderate, or severe). We used multiple logistic regression to analyze influencing factors on the degree of skin itching in patients with UP.

Results: (1) Of the 148 patients, 60 had uremic pruritus (incidence rate, 40.54%). These included 22 cases of mild skin itching (14.86%), 30 of moderate skin itching (20.27%), and 8 of severe skin itching (5.41%). (2) Compared with uremia patients without skin pruritus, patients with UP had higher levels of PTH, Hb, BUN, and hs-CRP. (3) The composition ratio showed significant differences between urban and rural patients with different degrees of skin itching (*P* = 0.017); moreover, the difference of PTH and hs-CRP levels was statistically significant (*P* = 0.009 and < 0.001, respectively). (4)Using no itching as a reference, multiple logistic-regression analysis showed that as hs-CRP level increased, the patient's risks of mild skin itching (odds ratio [OR] = 1.740; 95% confidence interval [CI], 1.061–2.854;

P = 0.028), moderate skin itching (OR = 2.8838 95% CI, 1.744–4.718; P < 0.001), and severe skin itching (OR = 9.440; 95% CI, 3.547–25.124; P < 0.001) all increased as well. Compared with urban residents, rural residents have a higher risk of moderate itching (OR = 3.869; 95% CI, 1.099–13.622; P=0.035).

Conclusion: Levels of hs-CRP were associated with degree of skin itching in patients with UP. Higher hs-CRP levels were closely related to severe skin itching.

Introduction

Background

Itchy skin is a common and distressing symptom in patients with chronic kidney disease. Uremic pruritus mainly manifests as skin itching of varying degrees, either systemically or locally, of which the back, extremities, chest, and head are common. Itching appears as paroxysmal episodes of varying duration. Those with milder symptoms can have intermittent attacks, lasting several minutes each time, while those with more severe symptoms last longer, and the symptoms are usually most obvious at night [1]. Epidemiological data indicated that approximately 40% of patients with end-stage renal disease (ESRD) experience moderate to severe itching [2]. The Dialysis Outcomes Practice Patterns Study (DOPPS), conducted from 2012 to 2015, showed that 26%–48% of patients had at least moderate skin itching, whereas 13%–26% of patients had severe or extreme itching [3]. The higher the degree of skin itching, the higher the mortality rate [4]. Several causes of or factors contributing to uremic pruritus (UP) have been proposed, including increasing systemic inflammation; abnormal levels of serum parathyroid hormone (PTH), serum calcium (Ca), and serum phosphorus (P); opioid receptor imbalance; and neuropathological processes [2]. However, the factors affecting degree of skin itching in patients with uremia are still unclear. We used a five-dimensional itching scale [5] to evaluate skin itching across multiple dimensions in uremic patients undergoing maintenance HD. We also included general patient data such as age, sex, body mass index (BMI), place of residence, educational level, Diabetes mellitus status,and duration of HD; as well as clinical indicators such as Ca, P, serum albumin (ALB), hemoglobin (Hb), PTH, serum creatinine (sCr) ,serum urea nitrogen（BUN), urea nitrogen clearance index (KT/V), ferritin (FER) before dialysis, and hs-CRP to analyze the determinants of itching in these patients.

objectives

1. explore the prevalence of UP in maintenance HD patients and the demographic and clinical characteristics of pruritus patients.

2. analyze the relationship between high-sensitivity C-reactive protein and different degrees of skin itching.

Methods

Study design

This study is a cross-sectional study on UP in patients receiving HD. This study was approved by the

Ethics Committee of Xinchang County People’s Hospital, Zhejiang Province, China (Approval No. XCXRMYY2018-001).

Study setting

we included a total of 148 subjects who received HD at the Blood Purification Center of Xinchang County People's Hospital, Zhejiang Province, China from March 2019 to June 2019. each of them signed an informed consent form. Collection of data was by the authors or research personnel, and all personnel received training and guidance from the corresponding author. Data statistics and analysis were from September to December 2019.

Participants

Inclusion criteria were as follows: (1) ≥1 month of HD in dialysis units and (2) age 20–90 years. Exclusion criteria were as follows: (1) primary skin diseases (e.g., eczema, psoriasis, neurodermatitis, allergic dermatitis, and drug rash), (2) use of right-cyclic saccharin, analgesics, antibiotics, or lipid-lowering drugs, (3) peripheral neuropathy, thyroid disease, leukemia, lymphoma, bile siltation, liver lesions, or pregnancy, (4) patients with hearing/communication impairments who could not complete the study, and (5) patients with infections, malignant tumors, cardiovascular disease, or tissue damage. These criteria were applied to each patient.

Variables

The diagnostic criteria for uremic pruritus adopted internationally are as follows: (1) uremia patients exclude skin pruritus caused by other diseases; (2) itching occurs at least 3 days within 2 weeks, and itches several times a day, each time itching It lasts a few minutes and affects the patient's life; (3) Itching in a specific pattern lasts for more than 6 months[6]. We used the Five-Dimensional Itching Scale (5DIS) to assess itching based on the five dimensions of degree, duration, direction, disability, and distribution. Total score ranged from 5 (no itching) to 25 (most severe itching). Patients with scores <5 points were defined as having no itching; those with scores 6–25 points were considered itch patients. Score of 6–10 points indicated mild itching, 11–20 points indicated moderate itching, and 21–25 points indicated severe Itching. Diagnosis of uremia pruritus meets the following conditions: 1. The total score of pruritus according to 5DIS is between 6-25 points. 2. Meet the international diagnostic criteria for uremic pruritus (2) and (3). 3. Meet the exclusion criteria. ultimately, we included a total of 60 patients with UP and 88 patients without UP. BMI (kg/m^2) was calculated using the following formula: weight (kg) / height $(m)^2$. Lean patients were defined as those with BMI ≦18.4 kg/m^2, normal patients with BMI 18.5–23.9 kg/m^2, heavy patients with BMI 24.0–27.9 kg/m^2, and obese patients with BMI ≧28.0 kg/m^2. According to the World Health Organization (WHO) standards, elderly patients were defined as those age ≧65, and non-elderly patients as those age <65.

Data source/measurement

Patients data included Sex, Age, BMI, Place of residence (rural or urban), educational level (illiterate or semi-literate, primary, junior high school, high school, or university or above), diabetes mellitus (Yes or No) and duration of maintenance HD (Months), which were obtained by the investigator interviewing the patients. We assayed biochemical indicators such as Ca, P, iPTH, Hb, ALB, sCr, Bun, FER, sp/KTV and hs-CRP. The hospital's central laboratory performed all of the laboratory tests, and auto-analyzers were

used to determine biochemical data, iPTH was measured with Roche second-generation assay. The clinical biochemical indicators were based on the data within three months from the survey day. If there is no data within three months, the patient is required to re-test. If the patient is unwilling to cooperate with the test, it is a missing value. spKT/V(single pool KT/V) $= -\ln(R - 0.008t) + (4 - 3.5R) * (\Delta BW/BW)$. Note: K refers to Blood urea clearance rate of dialyzer（L/h）, T is the dialysis time (h), and V is the distribution volume of urea (V). R is the ratio of blood urea nitrogen after dialysis to blood urea nitrogen before dialysis; t is single dialysis time in h; ΔBW is the weight change value before and after dialysis, i.e. ultrafiltration, unit L; BW is weight in kg. Blood collection requirements: blood samples before dialysis from the artery end of the vascular path, after dialysis before blood sample collection to stop ultrafiltration, reduce blood flow of 50 ml/min, wait 15 seconds after blood collection from the artery as a blood sample after dialysis.

Bias

1.Non-response bias

This study recruited 160 patients, 3 of whom were non-responders, and these non-responders may have differed from responders in some critical clinical features or exposures; however, the proportion (1.87%) was small.

2.Subject bias and researcher bias

During participation in the questionnaire of this study, some patients may be limited by their cultural level or subjective perceptions, which may lead to discrimination in the individual skin itch scale scores, thus affecting the accuracy of the assessment of the itchiness of the patient's skin. For the study participants who took part in the questionnaire, we trained two research staff, and the final results were taken as the mean of the two-pruritus skin itch scale scores.

3. selective bias

Of the 60 patients with itchy skin, 22 had mild skin pruritus, 30 had moderate skin pruritus, and 8 had severe skin pruritus. The proportion of patients with severe skin pruritus was small (13.33%), resulting in an uneven balance of patients between the groups.

Study size

Epidemiological data indicated that approximately 40% of patients with end-stage renal disease (ESRD) experience UP [2]. The overall prevalence of uremia and pruritus is 0.4, the tolerance is set to 0.1, taking a=0.05, the total number of hemodialysis in our blood purification center is 180 people. According to PASS 15 Power Analysis and Sample Size Software (NCSS, LLC. Kaysville, Utah, USA, ncss.com/software/pass), the calculated sample size is 121 people, assuming that the dropout rate is 20%, and the total sample size is 152 people.

Quantitative variables

Quantitative data with a normal distribution is expressed as mean ± standard deviation; intergroup comparisons were made using one-way analysis of variance or t-test; quantitative data with a non-normal distribution are expressed as medians, and interquartile ranges, intergroup comparisons of continuous variables were made using a non-parametric test.

Statistical methods

We performed statistical processing using line data from SPSS software version 24.0 (IBM Corp., Armonk, New York, US). Intergroup comparison of classification variables was based on the rank-sum or chi-square test. A multiple logistic regression model analyzed the factors associated with degree of skin itching in patients with uremia. All variables were diagnosed by collinearity; after elimination of the collinearity problem, variables of P<0.05 in single-factor regression analysis and variables with statistical differences between patients with different degrees of skin itching were included in the regression model. Finally, we included four factors including Hb, PTH, hs-CRP, Place of residence in the multiple logistic-regression model. $P < 0.05$ was considered to be statistically significant. In this study, the variables iPTH, hs-CRP, BUN, KT/V have two missing values, respectively, a total of 8 cases had missing values. The analysis of missing values indicates that these variables were not MCAR (missing completely at random), so this study uses the EM (expectation-maximization) algorithm to fill in.

Results

Participants

we compiled 160 cases of maintenance HD patients who were treated from March 2019 to June 2019 according to sampling survey, 3 of whom did not respond. We excluded 1 case of cirrhosis, 1 of dialysis allergy, 1 of drug rash, 3 of nodule itch, and 3 of hearing impairment that rendered the patient unable to communicate; ultimately, we included a total of 148 eligible subjects, in which 89 males (60.1%) and 60 females (39.9%). The median duration of HD was 42 months. There were 60 cases of UP (prevalence rate, 40.54%), including 22 mild cases (14.86%), 30 moderate cases (20.27%), and eight severe cases (5.41%), As shown in Fig 1.

Fig 1. Patient screening and grouping.

Descriptive data

The primary clinical characteristics of the included patients are shown in Table 1.

Table 1. Demographic and clinical characteristics of patients enrolled at baseline.

Variable	Results
Sex	
Male	89 (60.1%)
Female	59 (39.9%)
Age, years	
Non-elderly (<65 years)	99 (66.9%)
Elderly (≥65 years)	49 (33.1%)
Educational level	
Illiterate or semi-literate	23 (15.5%)
Primary school	36 (24.3%)
Junior high school	54 (36.3%)
High school	19 (12.8%)
University and above	16 (10.8%)
Place of residence	
Rural	100 (67.6%)
Urban	48 (32.4%)
BMI, kg/m²	
≦18.4	25 (16.9%)
18.5–23.9	101 (68.3%)
24–27.9	15 (10.1%)
≧28	7 (4.7%)
Diabetes mellitus	
Yes	35(23.6)

No	113(76.4%)
Ca, mmol/l	2.23 (2.10–2.34)
P, mmol/l	1.74 (1.48–2.22)
iPTH, pg/mL	308.50 (153.50–581.50)
Hb, g/L	102.00 (95.25–110.00)
ALB, g/L	38.25 (35.20–40.00)
sCR, µmol/L	840.00 (666.00–910.00)
BUN, mmol/L	19.50(18.10-21.08)
KT/V	1.27(1.23-1.31)
F/ER, ng/ml	193.80(129.63-220.00)
hs-CRP, mg/L	1.11 (0.60–2.00)
Duration of HD, months	42.00 (18.50–77.25)

Notes: BMI, body mass index; Ca, serum calcium; P, serum phosphorus; iPTH, intact parathyroid hormone; Hb, hemoglobin; ALB, serum albumin; sCr, serum creatinine; BUN, urea nitrogen; KT/V, Urea nitrogen clearance index ; FER, ferritin; hs-CRP, serum hypersensitive C-reactive protein.

Outcome data

Compared with uremia patients without skin pruritus, patients with UP had higher levels of IPTH, Hb, BUN and hs-CRP. No statistically significant differences were found in demographic characteristics such as Sex, Age, educational level, BMI, Diabetes mellitus status or Place of residence; in levels of Ca, P, ALB, FER, KT/V, or sCr; or in HD duration (Table 2).

Table 2. Demographic and clinical characteristics of patients with and without uremic pruritus.

Variable	Pruritic group (n = 60)	Non-pruritic group (n = 88)	*P*
Sex			0.318
Male	39 (65%)	50 (56.8%)	
Female	21 (35%)	38 (43.2%)	
Age, years			0.083
<65 years	45 (75%)	54 (61.4%)	
≥65 years	15 (15%)	34 (38.6%)	
Educational level			0.623
Illiterate or semi-literate	7 (11.7%)	16 (18.2%)	
Primary school	13 (21.7%)	23 (26.2%)	
Junior high school	28 (46.7%)	26 (29.5%)	
High school	8 (13.3%)	11 (12.5%)	
University and above	4 (6.7%)	12 (13.6%)	
Place of residence			0.602
Rural	42 (70%)	58 (65.9%)	
Urban	18 (30%)	30 (34.1%)	
BMI, kg/m^2			0.079
≦18.4	13 (21.7%)	12 (13.6%)	
18.5–23.9	41 (68.3%)	60 (68.2%)	
24–27.9	5 (8.3%)	10(11.4%)	
≥28	1 (1.7%)	6 (6.8%)	
Diabetes mellitus			0.099
Yes	10 (16.7%)	25 (28.4%)	
No	50 (83.3%)	63 (71.6%)	
Ca, mmol/l	2.24 (2.13–2.36)	2.19 (2.09–2.34)	0.211
P, mmol/l	1.85 (1.50–2.34)	1.72 (1.44–2.15)	0.117
iPTH, pg/ml	368.00 (229.75–754.25)	220.50 (111.75–503.25)	0.001*
Hb, g/L	104.00 (98.25–111.75)	100.00 (91.00–109.00)	0.025*
ALB, g/L	38.10 (35.13–40.00)	38.45 (35.80–40.08)	0.972
sCr, µmol/L	853.00 (686.00–950.00)	824.00 (629.25–899.75)	0.420

BUN, mmol/L	19.95（18.28-21.65）	19.10（17.60-20.38）	0.028*
KT/V	1.27（1.24-1.31）	1.27（1.22-1.32）	0.567
FER, ng/ml	200.20（127.58-218.25）	190.50（129.63-221.50）	0.696
hs-CRP, mg/L	2.00 (1.00–3.00)	0.90 (0.50–1.50)	<0.001*
Duration of HD, months	45.50 (18.00–77.00)	42.00 (20.25–78.75)	0.936

Notes: The above numerical variables are all non-normal distribution data, articulated by median (range). Intergroup comparisons of variables were made using the Mann–Whitney *U* test. Categorical variables are expressed as counts (percentages). Unordered categorical variables were tested by chi-square; ordered categorical variables such as BMI and educational level were tested by Kruskal–Wallis *H*. BMI, body mass index; Ca, serum calcium; P, serum phosphorus; iPTH, intact parathyroid hormone; Hb, hemoglobin; ALB, serum albumin; sCr, serum creatinine; BUN, urea nitrogen；KT/V, Urea nitrogen clearance index; FER, ferritin; hs-CRP, serum hypersensitive C-reactive protein.*$P < 0.05$.

The composition ratio showed a difference between rural and urban patients with varying degrees of skin itching ($P = 0.017$). Differences in IPTH and hs-CRP levels of patients with different degrees of skin itching were statistically significant ($P = 0.009$ and <0.001, respectively), As shown in Table 3.

Table 3. Baseline characteristics of patients with varying degrees of skin itching.

**Variable**	**No itching (n = 88)**	**Mild itching (n = 22)**	**Moderate itching (n = 30)**	**Severe itching (n = 8)**	***P***
Sex					0.495
Male	50 (56.8%)	16 (72.7%)	19 (63.3)	4 (50%)	
Female	38 (43.2%)	6 (27.3%)	11 (36.7)	4 (50%)	
Age, years					0.262
<65	54 (61. 4%)	15 (68.2%)	24 (80.0%)	6 (75.0%)	
≥65	34 (38.6%)	7 (31.8%)	6 (20.0%)	2 (25.0%)	
Educational level					0.478
Illiterate/semi-literate	16 (18.2%)	2 (9.1%)	4 (13.3%)	1 (12.5%)	

Primary school	23 (26.1)	3 (13.6%)	8 (26.7%)	2 (25%)	
Junior high school	26 (29. 5%)	11 (50.0%)	12 (40.0%)	5 (62.5%)	
High school	11 (12.5%)	2 (9.1%)	6 (20.0%)	0 (0.0%)	
University and above	12 (13.6%)	4 (18.2%)	0 (0.0%)	0 (0.0%)	
Place of residence					0.017*
Rural	58 (65.9%)	10 (45.5%)	25 (83.3%)	7 (87.5%)	
Urban	30 (34.1%)	12 (54.5%)	5 (16.7%)	1 (12.5%)	
BMI, kg/m2)					0.300
≦18.4	12 (13.6%)	4 (18.2%)	6 (20.0%)	3 (37.5%)	
18.5–23.9	60 (68.2%)	16 (72.8%)	21 (70.0%)	4 (50.0%)	
24–27.9	10 (11.4%)	1 (4.5%)	3 (10.0%)	1 (12.5%)	
≧28	6 (6.8%)	1 (4.5%)	0 (0.0%)	0 (0.0%)	
Diabetes mellitus					0.326
Yes	25（28.4%）	4（18.2%）	4（13.3%）	2（25%）	
No	63（71.6%）	18（81.8%）	26（86.7%）	6（75%）	
Ca, mmol/L	2.19 (2.09–2.34)	2.25 (2.15–2.33)	2.24 (2.12–2.45)	2.17 (2.05–2.31)	0.485
P, mmol/L	1.72 (1.44–2.15)	1.90 (1.50, 2.47)	1.90 (1.62, 2.25)	1.49 (1.30–2.26)	0.227
IPTH, pg/mL	220.50 (111.75–503.25)	377.50 (280.00–719.00)	360.00 (207.00–793.34)	497.50 (229.50–801.25.)	0.009*
Hb, g/L	100.00 (91.00–109.00)	103.50 (98.75–109.25)	105.00 (98.75–109.25)	107.00 (93.25–118.75)	0.138
ALB, g/L	38.45 (35.80–40.08)	38.05 (36.68–40.85)	38.25 (34.73–39.63)	36.60 (31.73–39.83)	0.472
sCr, μmol/L	824.00 (629.25–899.75)	853.00 (710.00–974.75)	850.50 (709.50–955.00)	788.50 (422.75–905.00)	0.565

BUN, mmol/L	19.10（17.60-20.38）	19.90（18.18-22.43）	19.80（18.18-21.20）	20.35（19.43-21.73）	0.118
KT/V	1.27（1.22-1.32）	1.28（1.26-1.33）	1.27（1.23-1.31）	1.27（1.22-1.33）	0.708
FER, ng/ml	190.50（129.63-221.50）	203.00（116.50-231.00）	200.50（142.26-206.00）	153.75（109.25-203.85）	0.628
hs-CRP, mg/L	0.90 (0.50–1.50)	1.08 (0.50–2.63)	2.15 (1.18–2.93)	3.93 (2.83–5.18)	<0.001*
HD duration, months	42.00 (20.25–78.75)	38.50 (17.25–83.25)	37.00 (14.00–68.25)	67.00 (56.00–89.25)	0.119

Notes: P, Hb, ALB, Ca, PTH, sCr, hs-CRP, and HD duration are all non-normal data, articulated by median (range); the rest are conveyed by n (%). Kruskal–Wallis probability between the four groups, with subsequent multiple Mann–Whitney *U* test if significant. Categorical variables are expressed as counts (percentages), unordered categorical variables were tested by chi-square, and ordered categorical variables were tested by Kruskal–Wallis *H*. BMI, body mass index; Ca, serum calcium; P, serum phosphorus; iPTH, intact parathyroid hormone; Hb, hemoglobin; ALB, serum albumin; sCr, serum creatinine; BUN, urea nitrogen；KT/V, Urea nitrogen clearance index；FER, ferritin; Hb, Hemoglobin; ALB, serum albumin; hs-CRP, serum hypersensitive C-reactive protein; HD, hemodialysis.*$P < 0.05$.

Main results

Degree of skin itching in patients with uremic pruritus as analyzed by the single-factor logistic-regression model

Compared with patients without uremic pruritus, Mild itching (OR = 1.917; *P* = 0.010), moderate itching (OR = 2.696, P < 0.001), and severe itching (OR = 6.915; *P* < 0.001) were all related to hs-CRP level. The higher hs-CRP level was closely related to severe itching. iPTH was weakly associated with severe itching (OR = 1.001, *P* = 0.031). Hb was slightly associated with moderate itching (OR = 1.036, *P* = 0.026) and severe itching (OR=1.036, P=0.170), as shown in (Fig 2, S1 Table).

Fig 2. Single-factor logistic-regression analysis of degree of skin itching in patients with uremic pruritus. the error bars represent the lower and upper limits of the 95% confidence interval. the symbol is a circle for mild itching, a triangle for moderate itching, and a square for severe itching. The dashed line in the figure indicates that the odds ratio is equal to 1.

Degree of skin itching in patients with uremic pruritus as analyzed by multivariate logistic regression

We included four variables (hs-CRP, Hb, PTH, Place of residence) in multiple logistic-regression analysis. Using no itching as a reference, the results showed that risk of skin itching increased with level of hs-CRP (mild skin itching, OR = 1.740; 95% CI, 1.061–2.845; *P* = 0.028; moderate skin itching, OR = 2.838; 95% CI, 1.744–4.618; *P* < 0.001; severe skin itching, OR = 9.440; 95% CI, 3.547–25.124; *P* < 0.001) . Compared with urban residents, rural residents have a higher risk of moderate itching (OR = 3.869; 95% CI, 1.099–13.622; *P*=0.035). as shown in (Fig 3, S2 Table).

Fig 3. Multiple logistic-regression analysis of factors influencing skin itching in patients with uremic pruritus. the error bars represent the lower and upper limits of the 95% confidence interval. the symbol is a circle for mild itching, a triangle for moderate itching, and a square for severe itching. The dashed line in the figure indicates that the odds ratio is equal to 1.

Other analyses

Levels of hs-CRP in patients with moderate and severe skin itching were statistically significantly higher than in patients without Itching (all *P* <0.001); the level of iPTH in patients with mild Itching was higher than in those with no skin itching, to a statistically significant degree (*P* <0.05). There was no statistically significant difference in Place of residence in patients with mild, moderate and severe Itching compared with no skin itching (all *P* > 0.05), As shown in Fig 4.

Fig 4. Comparison of patients with varying degrees of skin itching and varying hs-CRP and IPTH levels. (A) Intergroup comparison of hs-CRP levels; **(B)** intergroup comparison of iPTH levels; **(C)** intergroup comparison of the composition ratio of rural to urban residents. *****P* < 0.0001, **P* < 0.05. Error line represents the minimum and maximum values.

Discussion

Key results

In this study, we found that among 148 eligible patients receiving maintenance HD, 60 had UP (prevalence rate, 40.54%); they included 22 mild cases of skin itching (14.86%), 30 moderate cases (20.27%), and 8 severe cases (5.41%). Compared with patients with no skin pruritus, patients with UP had higher levels of iPTH, Hb, BUN and hs-CRP. The composition ratio of rural to urban patients, levels of IPTH and hs-CRP were statistically significantly different in patients with varying degrees of skin pruritus. Single-factor logistic-regression analysis discovered a link between hs-CRP levels and all three degrees of itching. Moreover, Hemoglobin is associated with moderate and severe Itching, and iPTH is related to severe Itching. Multi-factor logistic regression analysis also indicated that patients with higher hs-CRP levels had higher risks of mild, moderate, and severe skin itching. Interestingly, the multivariate analysis also showed that rural residents have a higher risk of moderate skin itching than urban residents.

Limitations

Our study had some limitations. Firstly, it was a single-center, cross-sectional study that did not show a causal relationship between severity of skin itching in UP and hs-CRP, so further validation is needed from multicenter, large-sample, prospective cohort studies. Secondly, this study did not include some of the influences known in the literature such as normalized protein catabolic rate (nPCR), diet, insulin resistance, interleukins 6 and 2, and κ opioid receptor distribution. Thirdly, the number of patients with severe pruritus is small (n = 8), so readers may not understand a meaningful association with severe pruritus. Finally, the sample size of this study was small and uneven between groups.

Interpretation

This study showed that median hs-CRP level gradually increased along with degree of skin itching in patients with uremic pruritus (no itching, 0.90; mild skin itching, 1.08; moderate skin itching, 2.15; severe skin itching, 3.93). Furthermore, hs-CRP levels in patients with moderate and severe skin itching were significantly higher than in those without itching. These findings suggested that hs-CRP levels might be associated with severity of skin itching. Jiang et al. [7] compared the clinical effects of high-throughput hemodialysis (HTH) and hemodialysis filtration (HDF) treat patients with end-stage nephropathy and explored possible potential mechanisms. Their results showed that levels of C-reactive protein (CRP) and leukocyte interleukin-6 (IL-6) in patients receiving HTH significantly improved, compared with those in patients who received HDF. The study also found that HTH had an exact therapeutic effect in UP that was related to inhibition of microinflammation in dialysis patients. Another study also showed that HD patients with UP had significantly higher serum CRP and IL-6 levels compared to HD patients without UP, UP seems to be associated with an up-regulation of micro-inflammation in uremia [8]. Mahmudpour's team[9] indicated that the average reduction in VAS scores in the montelukast group was greater than that in the placebo group, and average levels of hs-CRP in this group showed a downward trend; however, average hs-CRP levels in the placebo group gradually increased, showing that the effect of montelukast treatment in UP might be related to a reduction in an inflammatory response. A factor analysis also shows a close correlation between UP and levels of the inflammatory factors CRP and leukocyte IL-6[10]. Based on this study and previous

studies, inflammatory responses appear to play an important role in UP.

We analyzed the factors affecting degree of skin itching in UP patients and corrected for patients' levels of Hb and PTH, Place of residence. Multi-factor logistic-regression analysis found that hs-CRP levels were associated with severity of skin itching and were a risk factor. Compared with patients without itching, the OR value for mild skin itching was 1.740, that for moderate skin itching was 2.838, and that for severe skin itching was 9.440, indicating that degree of skin itching increased along with hs-CRP levels. A previous multivariable logistic-regression analysis confirmed that older age and higher CRP levels are associated with severe itching [3]. Still, in our study, neither single-factor nor multi-factor logistic-regression analysis showed any differences in skin itching between elderly and non-elderly patients. In our study, the OR value for hs-CRP of patients with severe skin itching was significantly higher than that of patients without itching, suggesting that higher hs-CRP levels were closely associated with severe skin itching, which was consistent with the results of an earlier study. Pakfetrat et al. [11] assessed the effect of sertraline on UP. patients were randomly divided into two groups, the trial group receiving sertraline and the control group taking a placebo, and their severity of itching was measured by two scoring systems (visual-simulation score and DUO scoring system [12]). The researchers found that differences in skin itching intensity between the two scoring systems and between the two groups were directly related to CRP level, and they believed that sertraline reduced skin itching, perhaps due to its effect of reducing the number of inflammatory cytokines. Tseng et al.[13]studying a possible association between vegetarians and UP, found that levels of inflammatory factors such as leukocyte IL-2 and hs-CRP, visual-simulation score (Analog Score and VAS), and itching score were also lower in vegetarian patients than in non-vegetarian ones; this demonstrated that a vegetarian diet might be associated with amelioration of uremic-pruritus severity in hemodialysis patients.

In our study, the composition ratio showed a significant difference between rural and urban patients with different degrees of skin itching. Compared with patients without itching, the proportion of rural patients with moderate and severe skin itching was relatively high, however, no statistically significant difference was achieved. Although the single factor analysis did not indicate that the place of residence was related to the intensity of the patient's skin itching, the multi-factor analysis demonstrated that rural residents had a higher risk of moderate skin itching than urban residents. The possible reason is that on the one hand, compared with urban residents, rural residents have lower living habits and lower health awareness; on the other hand, with the development of urbanization, rural areas the air pollution in Xinchang's local rural areas is gradually increasing and the urban environment is slowly improving. UP in patients receiving maintenance HD is associated with air pollutants such as NO2 and CO [14]. A cross-sectional study suggested that UP in patients receiving maintenance HD might be associated with the number of days in which environmental particulate matter ≤2.5 μ in diameter (PM2.5) exceeds standard levels [15]. The above mentioned two studies suggest that itching of the skin might be associated with the patient's living environment. In this study, there were a total of 148 patients with uremia and eight patients with severe skin pruritus, accounting for 5.4% of the total patients.2012-2015 DPPSO study [3] surveyed 7,629 people, divided into five groups (not at all, somewhat, moderately, Very Much, Extremely) according to the degree of itchy skin. Extreme Itching accounted for 7%, which is slightly higher than 5.4% of patients with severe Itching in this study. The reason for the differences may lie in the different criteria used to assess the degree of pruritus in the patient's skin.

iPTH levels differed between patients with varying degrees of skin itching. Median iPTH level in

patients with severe skin itching was higher than that in patients without itching. Still, there was no statistically significant difference between iPTH levels across groups, and single-factor regression analysis suggested a weak association between higher iPTH levels and severe skin itching. However, after we corrected for hs-CRP and Hb levels, Place of residence, that association disappeared. After parathyroid excision, iPTH level and serum phosphate concentration are significantly reduced, and skin itching disappears [16,17]. A study by Moldovan et al. [18] suggests that the average value of PTH decreased significantly after parathyroidectomy, and the symptoms of bone pain were significantly improved. However, muscle weakness and itching of the skin did not improve. Levy et al. [19] studied the associated symptoms of sHPT in patients receiving maintenance HD. Their results showed that an increase in PTH levels was associated with skin itching worsening over time, and the effect was more obvious with larger changes in PTH level. However, the combination of the disease, detection of medication, inclusion of up to 19 symptoms, the larger *P*-value threshold (0.1), and other promiscuous factors might have affected the reliability of that study.

Some studies suggested that UP is related to Ca, P, ALB, and uremic toxins [11,20]. Nevertheless, contradictory data on the impact of Ca and P have been reported [21]. In this study, no statistically significant differences were found in Ca, P, ALB, and creatinine when comparing groups of patients with different degrees of skin pruritus. There may indeed be a lack of test power, which is considered to be related to the small sample size of this study and the unbalanced sample size among the groups. However, single-factor logistic regression analysis also did not find an association between these factors and the degree of skin pruritus. Therefore, these factors were ultimately not included in the multifactor logistic regression analysis. In fact, despite various substances have been discussed as potential pruritogens in chronic renal diseases, a causal relationship could never be established[22].

At present, only a few studies have explored the factors affecting skin itching intensity in these patients. Wieczorek et al. [23] documented Intensity of skin itching have a significant negative correlation with expression of κ opioid receptors in patients with HD. Also, hs-CRP levels, male sex, HD duration, insulin resistance, insufficient dialysis adequacy, and hyperphosphatemia are positively correlated with UP intensity [24], increasing the blood flow of the HD machine can reduce the intensity and frequency of skin itching [25]. However, the above preliminary research used visual-analogue scoring (VAS) to evaluate the degree of skin itching. In this study, we used a five-dimensional itching scale to evaluate skin itching across multiple dimensions in uremic patients undergoing maintenance HD. Although VAS is sufficient to assess the severity of symptoms, it does not take into account other aspects of pruritus, such as the relative impact of pruritus on quality of life, changes in pruritus over time, and the location of pruritus. The 5-D score was strongly correlated with the numerical rating scale（NRS）and VAS [5, 26], which is a questionnaire with acceptable validity, reliability and sensitivity to change to evaluate pruritus in Thai and Arabic patients [26, 27]. The 5-dimensional Pruritus Scale is recommended to assess the severity of pruritus because it is more accurate than VAS and more sensitive to the multidimensional nature of pruritus [28].

Generalizability

In conclusion, our study suggested that the intensity of skin itching in patients with UP was associated with hs-CRP and that higher hs-CRP levels were associated with severe skin itching. Thus, this study demonstrates that controlling inflammation can relieve itchy skin in patients with uremia, and is particularly clinically relevant in patients with severe itchy skin. Besides, the environment may be

related to the degree of itchy skin in uremia patients.

Supporting information

S1 Table. Single-factor logistic-regression analysis of degree of skin itching in patients with uremic pruritus.

S2 Table. Multiple logistic-regression analysis of factors influencing skin itching in patients with uremic pruritus.

Acknowledgements

We thank LetPub (www.letpub.com) for its linguistic assistance during the preparation of this manuscript.

Author contributions

Conceptualization: Jian-Hui Zhao, Yi-Wen Li.
Data curation: Qing-Shuang Zhu, Li-Li Wang.
Investigation: Jian-Hui Zhao, Yi-Wen Li, Qing-Shuang Zhu.
Methodology: Jian-Hui Zhao, Yi-Wen Li.
Project administration: Jian-Hui Zhao.
Supervision: Yi-Wen Li.
Writing – original draft: Jian-Hui Zhao.
Writing – review and editing: Jian-Hui Zhao, Yi-Wen Li, Qing-Shuang Zhu.

Funding

The authors received no specific funding for this work.

References

1. Kfoury LW, Jurdi MA. Uremic pruritus. J Nephrol. 2012;25(5):644-52. Epub 2011/10/11. doi: 10.5301/jn.5000039. PubMed PMID: 21983988.

2. Combs SA, Teixeira JP, Germain MJ. Pruritus in Kidney Disease. Semin Nephrol. 2015;35(4):383-91. Epub 2015/09/12. doi: 10.1016/j.semnephrol.2015.06.009. PubMed PMID: 26355256; PubMed Central PMCID: PMCPMC5497472.

3. Rayner HC, Larkina M, Wang M, Graham-Brown M, van der Veer SN, Ecder T, et al. International Comparisons of Prevalence, Awareness, and Treatment of Pruritus in People on Hemodialysis. Clin J Am Soc Nephrol. 2017;12(12):2000-7. Epub 2017/09/20. doi: 10.2215/CJN.03280317. PubMed PMID: 28923831; PubMed Central PMCID:

PMCPMC5718267.

4. Grochulska K, Ofenloch RF, Mettang T, Weisshaar E. Mortality of Haemodialysis Patients with and Without Chronic Itch: A Follow-up Study of the German Epidemiological Hemodialysis Itch Study (GEHIS). Acta Derm Venereol. 2019;99(4):423-8. Epub 2019/01/24. doi: 10.2340/00015555-3125. PubMed PMID: 30673111.

5. Elman S, Hynan LS, Gabriel V, Mayo MJ. The 5-D itch scale: a new measure of pruritus. Br J Dermatol. 2010; 162:587-93. https://doi.org/10.1111/j.1365-2133.2009.09586.x PMID: 19995367

6. Keithi-Reddy SR, Patel TV, Armstrong AW, Singh AK. Uremic pruritus. Kidney Int. 2007; 72:373-7. https://doi.org/10.1038/sj.ki.5002197 PMID: 17429345

7. Jiang X, Ji F, Chen ZW, Huang QL. Comparison of high-flux hemodialysis with hemodialysis filtration in treatment of uraemic pruritus: a randomized controlled trial. Int Urol Nephrol. 2016; 48:1533-41. https://doi.org/10.1007/s11255-016-1364-2 PMID: 27379625

8. Kimmel M, Alscher DM, Dunst R, Braun N, Machleidt C, Kiefer T, et al. The role of micro-inflammation in the pathogenesis of uraemic pruritus in haemodialysis patients. Nephrol Dial Transplant. 2006; 21:749-55. https://doi.org/10.1093/ndt/gfi204 PMID: 16249205

9. Mahmudpour M, Roozbeh J, Raiss Jalali GA, Pakfetrat M, Ezzat Zadegan S, Sagheb MM. Therapeutic Effect of Montelukast for Treatment of Uremic Pruritus in Hemodialysis Patients. Iran J Kidney Dis. 2017; 11:50-5. PMID: 28174353

10. Schricker S, Heider T, Schanz M, Dippon J, Alscher MD, Weiss H, et al. Strong Associations Between Inflammation, Pruritus and Mental Health in Dialysis Patients. Acta Derm Venereol. 2019; 99:524-9. https://doi.org/10.2340/00015555-3128 PMID: 30673107

11. Pakfetrat M, Malekmakan L, Hashemi N, Tadayon T. Sertraline can reduce uremic pruritus in hemodialysis patient: A double blind randomized clinical trial from Southern Iran. Hemodial Int. 2018; 22:103-9. https://doi.org/10.1111/hdi.12540 PMID: 28263039

12. Duo LJ. Electrical needle therapy of uremic pruritus. Nephron. 1987; 47:179-83. https://doi.org/10.1159/000184487 PMID: 3500424

13. Tseng CY, Wu TT, Lai CW, Lin HJ, Chou CY, Chang CT, et al. Vegetarian diet may ameliorate uremic pruritus in hemodialysis patients. Ren Fail. 2018; 40:514-9. https://doi.org/10.1080/0886022X.2018.1512871 PMID: 30396306

14. Huang W, Lin J, Weng C, Hsu C, Yen T. Environmental NO 2 and CO Exposure: Ignored Factors Associated with Uremic Pruritus in Patients Undergoing Hemodialysis. Sci Rep. 2016; 6:1-10.

15. Liu MH, Chan MJ, Hsu CW, Weng CH, Yen TH, Huang WH. Association of uremic pruritus in hemodialysis patients with the number of days of high mean 24-hour particulate matter with a diameter of <2.5 mum. Ther Clin Risk Manag. 2017; 13:255-62. https://doi.org/10.2147/TCRM.S129133 PMID: 28260912

16. Jing Y, Zhao H, Ge Y, Jia F, He Q, Wang S, et al. Application of total parathyroidectomy with auto-transplantation for uremia secondary hyperparathyroidism treatment. Int J Clin Exp Med. 2015; 8:11188.

17. Zhang Y, Lu Y, Feng S, Zhan Z, Shen H. Evaluation of laboratory parameters and symptoms after parathyroidectomy in dialysis patients with secondary hyperparathyroidism. Ren Fail. 2019; 41:921-9. https://doi.org/10.1080/0886022X.2019.1666724 PMID: 31573378

18. Moldovan D, Racasan S, Kacso IM, Rusu C, Potra A, Bondor C, et al. Survival after

parathyroidectomy in chronic hemodialysis patients with severe secondary hyperparathyroidism. Int Urol Nephrol. 2015; 47:1871-7. https://doi.org/10.1007/s11255-015-1106-x PMID: 26377492

19. Levy AR, Xing S, Brunelli SM, Cooper K, Finkelstein FO, Germain MJ, et al. Symptoms of Secondary Hyperparathyroidism in Patients Receiving Maintenance Hemodialysis: A Prospective Cohort Study. Am J Kidney Dis. 2020; 75:373-83.

20. Momose A, Shiraiwa Y, Narita S, Kusumi T, Goto S, Sera K. Total Calcium and Albumin Are Decreased in the Deeper Epidermis of Patients with Chronic Kidney Disease-Associated Pruritus. Nephron. 2017; 136:103-10. https://doi.org/10.1159/000458417 PMID: 28183080

21. Shirazian S, Kline M, Sakhiya V, Schanler M, Moledina D, Patel C, et al. Longitudinal predictors of uremic pruritus. J Ren Nutr. 2013; 23:428-31. https://doi.org/10.1053/j.jrn.2013.08.002 PMID: 24209894

22. Mettang T, Kremer AE. Uremic pruritus. Kidney Int. 2015; 87:685-91. https://doi.org/10.1038/ki.2013.454

23. Wieczorek A, Krajewski P, Kozioł-Gałczyńska M, Szepietowski JC. Opioid receptors expression in the skin of haemodialysis patients suffering from uraemic pruritus. J Eur Acad Dermatol Venereol. 2020. https://doi.org/10.1111/jdv.16360 PMID: 32170896

24. Abdelsalam M, Tawfik M, Reda EM, Eldeeb AA, Abdelwahab A, Zaki ME, et al. Insulin Resistance and Hepatitis C Virus-Associated Subclinical Inflammation Are Hidden Causes of Pruritus in Egyptian Hemodialysis Patients: A Multicenter Prospective Observational Study. Nephron. 2019; 143:120-7. https://doi.org/10.1159/000501409 PMID: 31311030

25. Aliasgharpour M, Zabolypour S, Asadinoghabi A, Haghani H, Lesanpezeshki M. The Effect of

Increasing Blood Flow Rate on Severity of Uremic Pruritus in Hemodialysis Patients: A Single Clinical Trial. J Natl Med Assoc. 2018; 110:270-5. https://doi.org/10.1016/j.jnma.2017.04.008 PMID: 29778130

26. Lai JW, Chen HC, Chou CY, Yen HR, Li TC, Sun MF, et al. Transformation of 5-D itch scale and numerical rating scale in chronic hemodialysis patients. BMC Nephrol. 2017; 18:56. https://doi.org/10.1186/s12882-017-0475-z PMID: 28178931

27. Khan TM, Al-Haider I, Syed Sulaiman SA, Hassali MA. Linguistic validation of the 5D itching scale to Arabic in patients with end-stage kidney disease. J Ren Care. 2013; 39:222-7. https://doi.org/10.1111/j.1755-6686.2013.12038.x PMID: 24152068

28. Nofal E, Farag F, Nofal A, Eldesouky F, Alkot R, Abdelkhalik Z. Gabapentin: A promising therapy for uremic pruritus in hemodialysis patients: A randomized-controlled trial and review of literature. J Dermatolog Treat. 2016; 27:515-9. https://doi.org/10.3109/09546634.2016.1161161 PMID: 27043168

Click here to access/download;Figure;Fig 1.tif

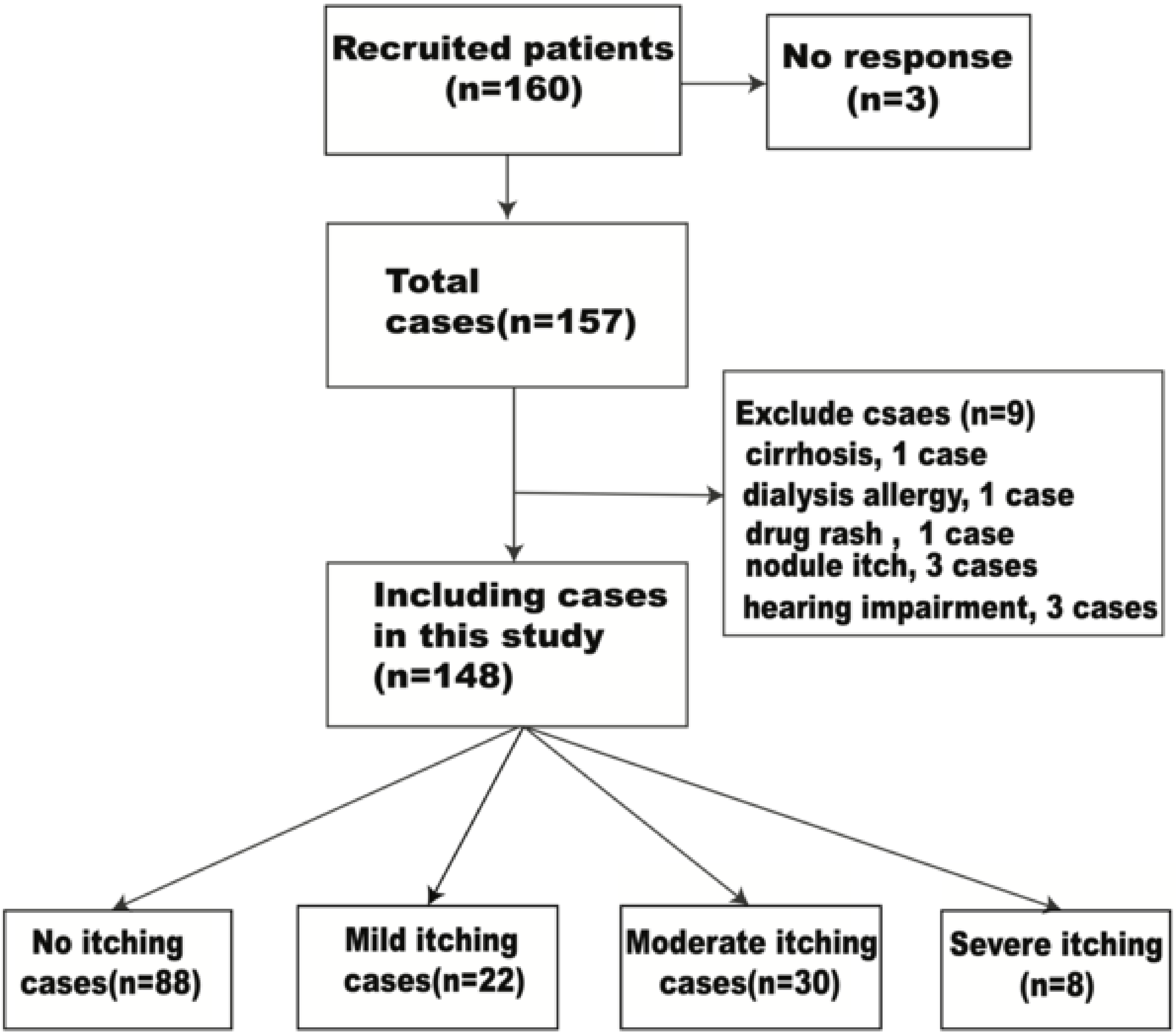

Click here to access/download;Figure;Fig 2.tif

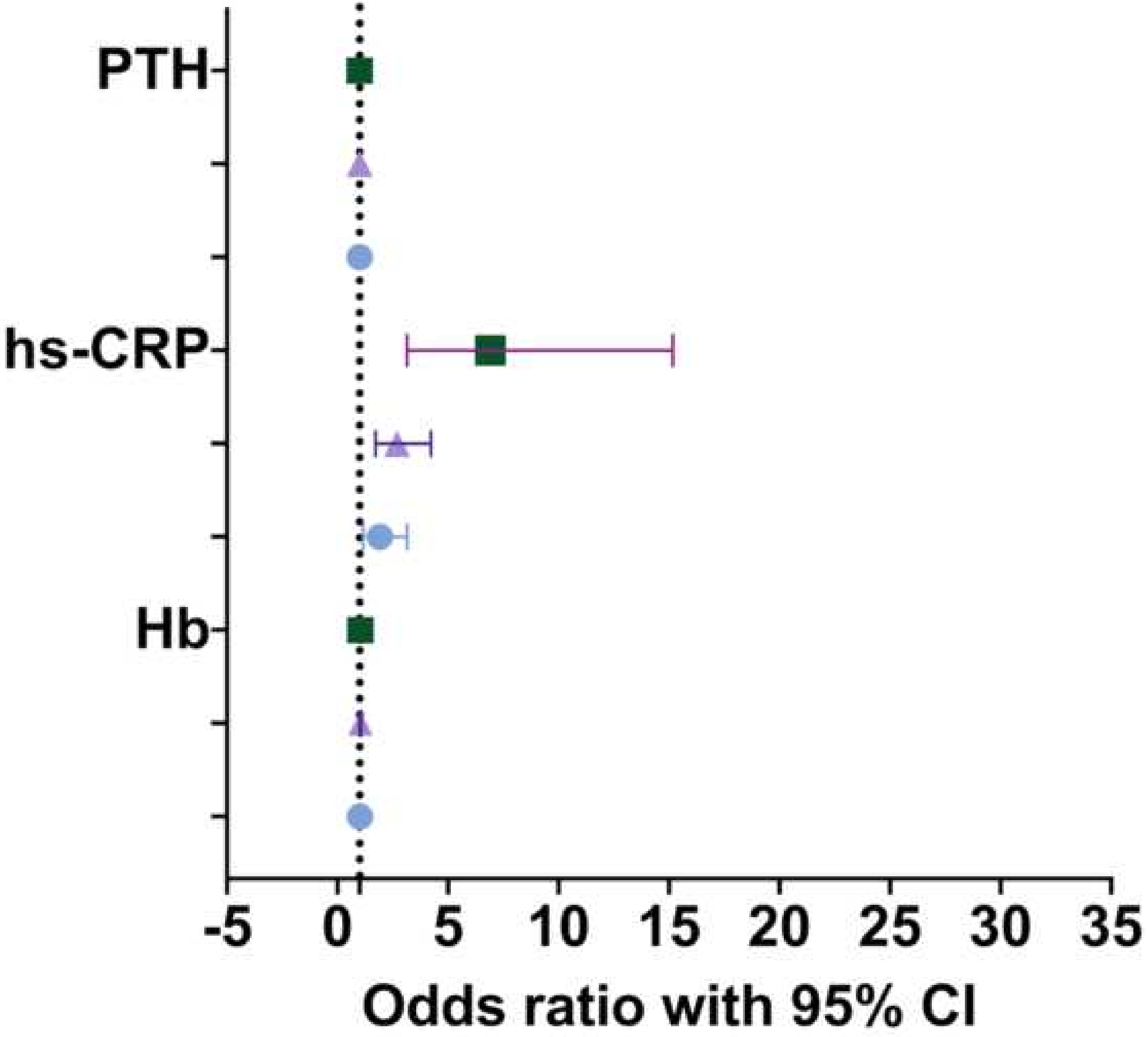

 Click here to access/download;Figure;Fig 3.tif

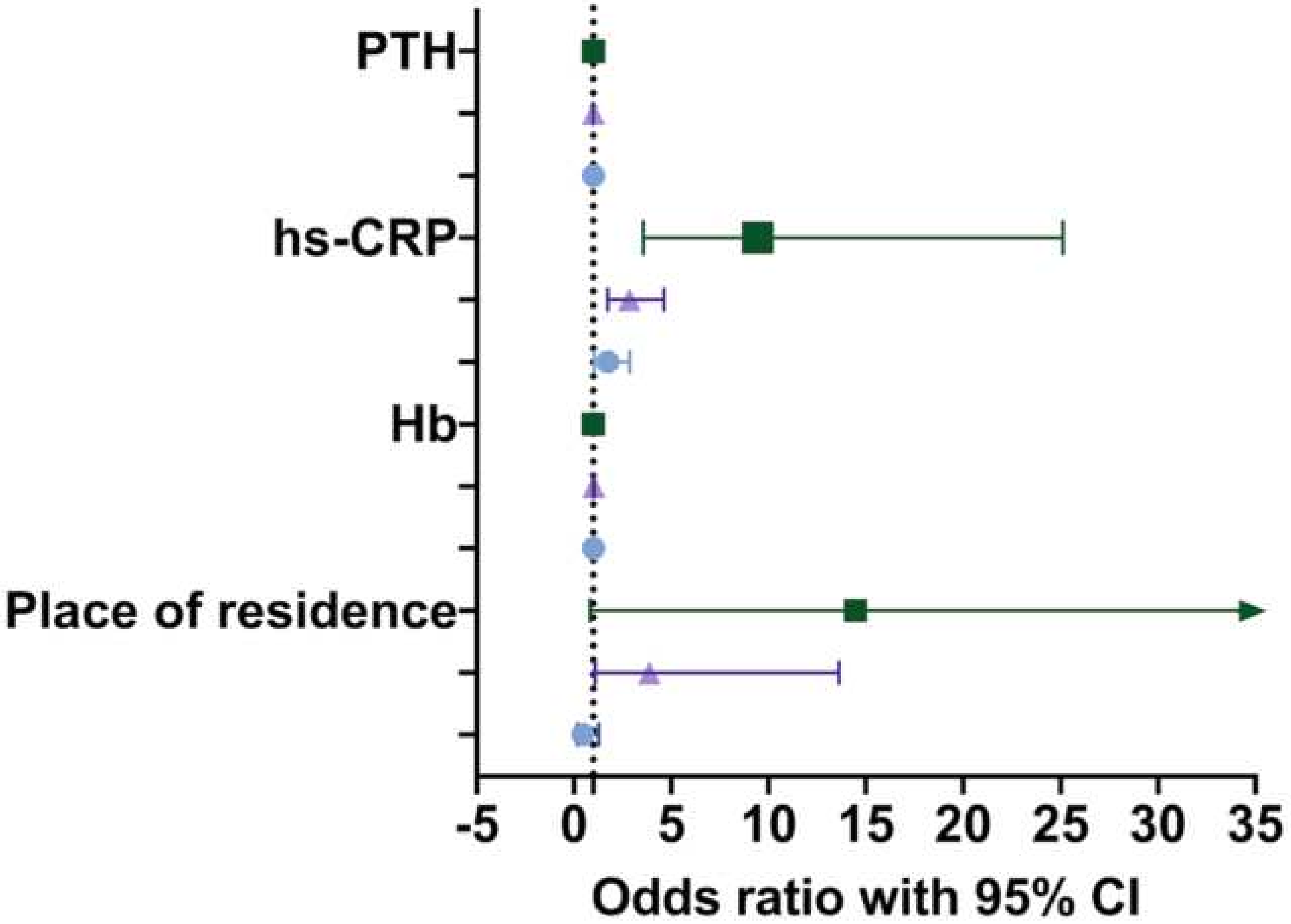

Click here to access/download;Figure;Fig 4.tif

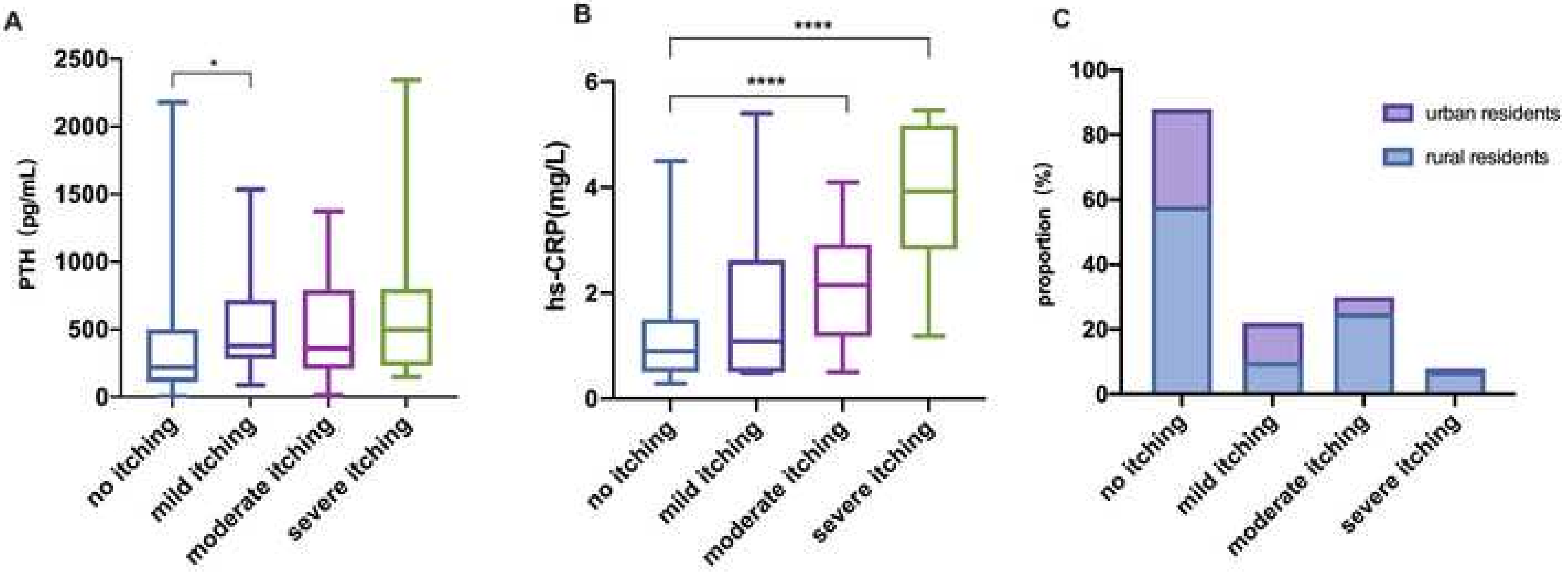

Click here to access/download
Supporting Information
S1 Table.docx

Click here to access/download
Supporting Information
S2 Table.docx

Determinants of the intensity of uremic pruritus in patients receiving maintenance hemodialysis: A cross-sectional study

Jian-Hui Zhao[1,2*] , Qiu-Shuang Zhu [1,2¶] , Yi-Wen Li[3&] , Li-Li Wang[2]

1 Department of Nephrology, Xinchang County People's Hospital, Xinchang, Shaoxing, Zhejiang Province, ChinaDepartment of Nephrology, Xinchang County People's Hospital, Zhejiang Province, Xinchang 312500,

2 Blood Purification Center, Xinchang County People's Hospital, Xinchang, Shaoxing, Zhejiang Province, China Blood Purification Center, Xinchang County People's Hospital, Zhejiang Province, Xinchang 312500,

3 DeparDepartment of Nephrology, Zhejiang People's Hospital, Hangzhou, Zhejiang Province, China tment of Nephrology, Zhejiang People's Hospital, Hangzhou, Zhejiang Province, 310000

* lyzhaojh@126.com

* Corresponding author

E-mail: lyzhaojh@126.com (Zhao, J.H.)

Abstract

Background: Uremic pruritus (UP) is a common and frustrating symptom in patients receiving hemodialysis (HD). This study aimed to investigate the determinants of the intensity of UP in patients receiving maintenance HD.

Methods: A cross-sectional study on uremic pruritus in male and female patients receiving HD was conducted in September 2019.This study included 148 eligible patients who received HD at the Blood Purification Center of Xinchang County People's Hospital, Zhejiang Province, China from March 2019 to June 2019. We collected general data such as age, sex, body mass index (BMI), place of residence, educational level,diabeteslevel, diabetes mellitus status and duration of HD; as well as clinical biochemical indicators, including serum calcium (Ca), serum phosphorus (P), serum albumin (ALB), hemoglobin (Hb), serum parathyroid hormone (PTH), pre-dialysis serum urea nitrogen (BUN), urea nitrogen clearance index (KT/V), ferritin (FER) and pre-dialysis serum creatinine (sCr). We also assayed the inflammatory cytokine serum high sensitivity C-reactive protein (hs-CRP). The Five-Dimensional Itching Scale (5DIS) was used to evaluate the degree of skin itching (none, mild, moderate, or severe). We used multiple logistic regression to analyze influencing factors on the degree of skin itching in patients with UP.

Results: (1) Of the 148 patients, 60 had uremic pruritus (incidence rate, 40.54%). These included 22 cases of mild skin itching (14.86%), 30 of moderate skin itching (20.27%), and 8 of severe skin itching (5.41%). (2) Compared with uremia patients without skin pruritus, patients with UP had higher levels of PTH, Hb, BUN,andBUN, and serum hypersensitive C-reactive protein (hs-CRP). (3) The

Formatted: Font: 18 pt

Formatted: Font: 16 pt

Formatted: Font: 16 pt

Formatted: Font: 16 pt

composition ratio showed significant differences between urban and rural patients with different degrees of skin itching (P = 0.017); moreover, the difference of PTH and hs-CRP levels was ~~differed to a~~ statistically significant ~~degree~~ (P = 0.0 09~~15~~ and < 0.001, respectively). (4)Using no itching as a ~~refenence~~reference, m~~M~~ultiple logistic-regression analysis showed that as hs-CRP level increased, the patient's risks of mild skin itching (odds ratio [OR] = 1.740~~20~~; 95% confidence interval [CI], 1.061~~044~~–2.854~~35~~; P = 0.028~~33~~), moderate skin itching (OR = 2.88384~~4;~~ 95% CI, 1.744~~64~~–4.718~~16~~; P < 0.001), and severe skin itching (OR = 9.440~~8.929~~; 95% CI, 3.547~~340~~–25~~3~~.124~~865~~; P < 0.001) all increased as well. Compared with urban residents, rural residents have a higher risk of moderate itching (OR = 3.869~~942~~; 95% CI, 1.099~~105~~–13~~4~~.622~~069~~; P=0.035).

Conclusion: Levels of hs-CRP were associated with degree of skin itching in patients with UP.

Higher hs-CRP levels were closely related to severe skin itching.

Formatted: Font: 16 pt

Introduction

Formatted: Font: 18 pt

Background

Formatted: Font: 18 pt

Itchy skin is a common and distressing symptom in patients with chronic kidney disease. Uremic pruritus mainly manifests as skin itching of varying degrees, either systemically or locally, of which the back, extremities, chest, and head are common. Itching appears as paroxysmal episodes of varying duration. Those with milder symptoms can have intermittent attacks, lasting several minutes each time, while those with more severe symptoms last longer, and the symptoms are usually most obvious at night [1]. Epidemiological data indicated that approximately 40% of patients with end-stage renal disease (ESRD) experience moderate to severe itching [2]. The Dialysis Outcomes Practice Patterns Study (DOPPS), conducted from 2012 to 2015, showed that 26%–48% of patients had at least moderate skin itching, whereas 13%–26% of patients had severe or extreme itching [3]. The higher the degree of skin itching, the higher the mortality rate [4]. Several causes of or factors contributing to uremic pruritus (UP) have been proposed, including increasing systemic inflammation; abnormal levels of serum parathyroid hormone (PTH), serum calcium (Ca), and serum phosphorus (P); opioid receptor imbalance; and neuropathological processes [2]. However, the factors affecting degree of skin itching in patients with uremia are still unclear. W~~w~~e used a five-dimensional itching scale [5~~9~~] to evaluate skin itching across multiple dimensions in uremic patients undergoing maintenance HD. We also included general patient data such as age, sex, body mass index (BMI), place of residence, educational level, Diabetes mellitus status,and duration of HD; as well as clinical indicators such as Ca, P, serum albumin (ALB), hemoglobin (Hb), PTH, serum creatinine (sCr) ,serum urea nitrogen（BUN), urea nitrogen clearance index (KT/V), ferritin (FER) before dialysis, and hs-CRP to analyze the determinants of itching in these patients. ~~The aims of this study are to:~~

objectives

Formatted: Indent: First line: 0 ch

1. explore the prevalence of UP in maintenance HD patients and the demographic and clinical characteristics of pruritus patients.

2. analyze the relationship between high-sensitivity C-reactive protein and different degrees of skin

itching.

explore determinants of higher pruritus intensity based on participants' clinical and biochemical parameters and their pruritus assessments by 5DIS at baseline.

Materials and methodsMethods

Formatted: Font: 18 pt

Formatted: Font: 18 pt

Study design

This study is a cross-sectional study on UP in patients receiving HD. This study was approved by the Ethics Committee of Xinchang County People's Hospital, Zhejiang Province, China (Approval No. XCXRMYY2018-001).

Formatted: Normal

Study setting

we included a total of 148 subjects who received HD at the Blood Purification Center of Xinchang County People's Hospital, Zhejiang Province, China from March 2019 to June 2019. each of them signed an informed consent form. Collection of data was by the authors or research personnel, and all personnel received training and guidance from the corresponding author. Data statistics and analysis were from September to December 2019.

Formatted: Normal

Formatted: Font: 10 pt, Not Bold

Pparticipants and data collection

Formatted: Font: 16 pt

Inclusion criteria were as follows: (1) ≥1 month of HD in dialysis units and (2) age 20–90 years. Exclusion criteria were as follows: (1) primary skin diseases (e.g., eczema, psoriasis, neurodermatitis, allergic dermatitis, and drug rash), (2) use of right-cyclic saccharin, analgesics, antibiotics, or lipid-lowering drugs, (3) peripheral neuropathy, thyroid disease, leukemia, lymphoma, bile siltation, liver lesions, or pregnancy, (4) patients with hearing/communication impairments who could not complete the study, and (5) patients with infections, malignant tumors, cardiovascular disease, or tissue damage.Thesedamage. These criteria were applied to each patient. Collection of data was by the authors or research personnel and all personnel received training and guidance from the first author. Patient data included sex, age, BMI, place of residence (rural or urban), educational level (illiterate or semi-literate, primary, junior high school, high school, or university or above), and duration of maintenance HD (months), which were obtained by the investigator interviewing the patients. We assayed biochemical indicators such as Ca, P, PTH, Hb, ALB, sCr, and hs-CRP. All of the laboratory tests were performed by the hospital's central laboratory, and auto-analyzers were used to determine biochemical data. The clinical biochemical indicators are based on the data within 3 months from the survey day. If there is no data within 3 months, the patient is required to re-test. If the patient is unwilling to cooperate with the test, it is a missing value. BMI (kg/m²) was calculated using the following formula: weight (kg) / height (m)². Lean patients were defined as those with BMI ≦18.4 kg/m², normal patients with BMI 18.5–23.9 kg/m², heavy patients with BMI 24.0–27.9 kg/m², and obese patients with BMI ≧8.0 kg/m². According to World Health Organization (WHO) standards, elderly patients were defined as those age ≧65, and non-elderly patients as those age <65.

Fig 1. Patient screening and grouping.

Variables

Determining UP

The diagnostic criteria for uremic pruritus adopted internationally are as follows: (1) uremia

Formatted: Normal, Indent: First line: 0 ch

patients exclude skin pruritus caused by other diseases; (2) itching occurs at least 3 days within 2 weeks, and itches several times a day, each time itching It lasts a few minutes and affects the patient's life; (3) Itching in a specific pattern lasts for more than 6 months[610]. We used the Five-Dimensional Itching Scale (5DIS) to assess itching based on the five dimensions of degree, duration, direction, disability, and distribution. Total score ranged from 5 (no itching) to 25 (most severe itching). Patients with scores <5 points were defined as having no itching; those with scores 6–25 points were considered itch patients. Score of 6–10 points indicated mild itching, 11–20 points indicated moderate itching, and 21–25 points indicated severe Itching. Diagnosis of uremia pruritus meets the following conditions: 1. The total score of pruritus according to 5DIS is between 6-25 points. 2. Meet the international diagnostic criteria for uremic pruritus (2) and (3). 3. Meet the exclusion criteria. ultimately, we included a total of 60 patients with UP and 88 patients without UP. BMI (kg/m^2) was calculated using the following formula: weight (kg) / height $(m)^2$. Lean patients were defined as those with BMI ≦18.4 kg/m^2, normal patients with BMI 18.5–23.9 kg/m^2, heavy patients with BMI 24.0–27.9 kg/m^2, and obese patients with BMI ≧28.0 kg/m^2. According to the World Health Organization (WHO) standards, elderly patients were defined as those age ≧65, and non-elderly patients as those age <65.

Data source/measurement

Patients data included Sex, Age, BMI, Place of residence (rural or urban), educational level (illiterate or semi-literate, primary, junior high school, high school, or university or above), diabetes mellitus (Yes or No) and duration of maintenance HD (Months), which were obtained by the investigator interviewing the patients. We assayed biochemical indicators such as Ca, P, iPTH, Hb, ALB, sCr, Bun, FER, sp/KTV and hs-CRP. The hospital's central laboratory performed all of the laboratory tests, and auto-analyzers were used to determine biochemical data, iPTH was measured with Roche second-generation assay. The clinical biochemical indicators were are based on the data within three months from the survey day. If there is no data within three months, the patient is required to re-test. If the patient is unwilling to cooperate with the test, it is a missing value. spKT/V (single pool KT/V) =-$\ln(R-0.008t)+(4-3.5R)*(\Delta BW/BW)$. Note: K refers to Blood urea clearance rate of dialyzer（L/h）, T is the dialysis time (h), and V is the distribution volume of urea (V). R is the ratio of blood urea nitrogen after dialysis to blood urea nitrogen before dialysis; t is single dialysis time in h; ΔBW is the weight change value before and after dialysis, i.e. ultrafiltration, unit L; BW is weight in kg. Blood collection requirements: blood samples before dialysis from the artery end of the vascular path, after dialysis before blood sample collection to stop ultrafiltration, reduce blood flow of 50 ml/min, wait 15 seconds after blood collection from the artery as a blood sample after dialysis.

Bias

1.Non-response bias

This study recruited 160 patients, 3 of whom were non-responders, and these non-responders may have differed from responders in some critical clinical features or exposures; however, the proportion (1.87%) was small.

2.Subject bias and researcher bias

During participation in the questionnaire of this study, some patients may be limited by their cultural level or subjective perceptions, which may lead to discrimination in the individual skin itch scale

scores, thus affecting the accuracy of the assessment of the itchiness of the patient's skin. For the study participants who took part in the questionnaire, we trained two research staff, and the final results were taken as the mean of the two-pruritus skin itch scale scores.

3. selective bias

Of the 60 patients with itchy skin, 22 had mild skin pruritus, 30 had moderate skin pruritus, and 8 had severe skin pruritus. The proportion of patients with severe skin pruritus was small (13.33%), resulting in an uneven balance of patients between the groups.

Formatted: Normal, Indent: First line: 0 ch

Study size

Formatted: Font: 16 pt

Epidemiological data indicated that approximately 40% of patients with end-stage renal disease (ESRD) experience UP [2]. The overall prevalence of uremia and pruritus is 0.4, the tolerance is set to 0.1, taking a=0.05, the total number of hemodialysis in our blood purification center is 180 people. According to PASS 15 Power Analysis and Sample Size Software (NCSS, LLC. Kaysville, Utah, USA, ncss.com/software/pass), the calculated sample size is 121 people, assuming that the dropout rate is 20%, and the total sample size is 152 people.

Quantitative variables

~~**Ethics**~~

Quantitative data with a normal distribution is expressed as mean ± standard deviation; intergroup comparisons were made using one-way analysis of variance or t-test; quantitative data with a non-normal distribution are expressed as medians, and interquartile ranges, intergroup comparisons of continuous variables were made using a non-parametric test.

~~This study was approved by the Ethics Committee of Xinchang County People's Hospital, Zhejiang Province, China (Approval No.XCXRMYY2018-001).~~

Statistical methods~~analysis~~

Formatted: Font: 16 pt

Formatted: Font: 16 pt

Formatted: Indent: First line: 0 ch

We performed statistical processing using line data from SPSS software version 24.0 (IBM Corp., Armonk, New York, US).~~We performed statistical processing using line data from SPSS software version 24.0 (IBM Corp., Armonk, New York, US). Quantitative data with a normal distribution are expressed as *x*±*s*, intergroup comparisons were made using One-way analysis of variance or t test; quantitative data with a non-normal distribution are expressed as medians and interquartile ranges, intergroup comparisons of continuous variables were made using a non-parametric test.~~ I~~i~~ntergroup comparison of classification variables was based on the rank-sum or chi-square test. A multiple logistic regression model analyzed the factors associated with degree of skin itching in patients with uremia. All variables were diagnosed by collinearity; after elimination of the collinearity problem, variables of *P*< ~~≤~~ 0.05~~10~~ in single-factor regression analysis and variables with statistical differences between patients with different degrees of skin itching were included in the regression model. Finally, we included four~~ive~~ factors including Hb, ~~P,~~ PTH, hs-CRP, Place of residence in the multiple logistic-regression model. $P < 0.05$ was considered to be statistically significant. In this study, the variables iPTH, hs-CRP, BUN, KT/V have two missing values, respectively, a total of 8 cases had missing values. The analysis of missing values indicates that these variables were not MCAR (missing completely at

random), so this study uses the EM (expectation-maximization) algorithm to fill in.

In this study, both the variables PTH and hs-CRP have 2 missing values, a total of 4 cases had missing values. Although the analysis of missing values indicates that the variables PTH and hs-CRP do not rule out MCAR (missing completely at random), this study still uses the EM (expectation maximization) algorithm to fill in cautiously.

Results

Participants

Baseline characteristics of included patients

we compiled 160 cases of maintenance HD patients who were treated from March 2019 to June 2019 according to sampling survey, 3 of whom did not respond. We excluded 1 case of cirrhosis, 1 of dialysis allergy, 1 of drug rash, 3 of nodule itch, and 3 of hearing impairment that rendered the patient unable to communicate; ultimately, we included a total of 148 eligible subjects, in which 89 males (60.1%) and 60 females (39.9%). The median duration of HD was 42 months. There were 60 cases of UP (prevalence rate, 40.54%), including 22 mild cases (14.86%), 30 moderate cases (20.27%), and eight severe cases (5.41%), As shown in Fig 1.

Fig 1. Patient screening and grouping. We recruited 160 hemodialysis patients, 3 of those did not respond, excluded 9 people, and finally included 148 patients in the study. 89 males (60.1%) and 60 females (39.9%). Median duration of HD was 42 months. There were 60 cases of UP (prevalence rate, 40.54%), including 22 mild cases (14.86%), 30 moderate cases (20.27%), and 8 severe cases (5.41%), As shown in Table 1.

Descriptive data

The primary clinical characteristics of the included patients are shown in Table 1.

Formatted: Indent: First line: 0 ch

Formatted: Font: 18 pt

Formatted: Indent: First line: 0 ch, Tab stops: 0.2", Left

Table 1. Demographic and clinical characteristics of patients enrolled at baseline.

Variable	Results
Sex	
Male	89 (60.1%)
Female	59 (39.9%)
Age, years	
Non-elderly (<65 years)	99 (66.9%)
Elderly (≥65 years)	49 (33.1%)
Educational level	
Illiterate or semi-literate	23 (15.5%)
Primary school	36 (24.3%)
Junior high school	54 (36.3%)
High school	19 (12.8%)
University and above	16 (10.8%)
Place of residence	
Rural	100 (67.6%)
Urban	48 (32.4%)
BMI, kg/m^2	
≦18.4	25 (16.9%)
18.5–23.9	101 (68.3%)
24–27.9	15 (10.1%)
≧28	7 (4.7%)
Diabetes mellitus	
Yes	35(23.6)

No	113(76.4%)
Ca, mmol/l	2.23 (2.10–2.34)
P, mmol/l	1.74 (1.48–2.22)
iPTH, pg/mL	308.50 (153.50–581.50)
Hb, g/L	102.00 (95.25–110.00)
ALB, g/L	38.25 (35.20–40.00)
sCR, μmol/L	840.00 (666.00–910.00)
BUN, mmol/L	19.50(18.10-21.08)
KT/V	1.27(1.23-1.31)
F/ER, ng/ml	193.80(129.63-220.00)
hs-CRP, mg/L	1.11 (0.60–2.00)
Duration of HD, months	42.00 (18.50–77.25)

Formatted Table

**Variable**	**Results**
Sex	
Male	89 (60.1%)
Female	59 (39.9%)
Age, years	
Non-elderly (<65 years)	99 (66.9%)
Elderly (≥65 years)	49 (33.1%)
Educational level	
Illiterate or semi-literate	23 (15.5%)
Primary school	36 (24.3%)
Junior high school	54 (36.3%)
High school	19 (12.8%)
University and above	16 (10.8%)
Place of residence	

Rural	100 (67.6%)
Urban	48 (32.4%)
BMI, kg/m²	
≦18.4	25 (16.9%)
18.5–23.9	10199 (68.36.9%)
24–27.9	157 (10.11.5%)
≧28	7 (4.7%)
Diabetes mellitus	
Yes	35(23.6)
No	113(76.4%)
Ca, mmol/l	2.23 (2.10–2.34)
P, mmol/l	1.74 (1.489–2.22)
iPTH, pg/mL	308.50299.50 (1531.50–581.50)
Hb, g/L	102.00 (95.25–110.00)
ALB, g/L	38.25 (35.20–40.00)
sCR, μmol/L	840.00 (666.00–910.00)
BUN,mmol/L	19.50(18.10-21.08)
KT/V	1.27(1.23-1.31)
F/ER, ng/ml	193.80(129.63-220.00)
hs-CRP, mg/L	1.11 (0.60–2.00)
Duration of HD, months	42.00 (18.50–77.25)
Degree of skin itching	60 (40.5%)
Mild	22 (14.86%)
Moderate	30 (20.27%)
Severe	8 (5.41%)

Notes: BMI, body mass index; Ca, serum calcium; P, serum phosphorus; iPTH, intactserum parathyroid hormone; Hb, hemoglobin; ALB, serum albumin; sCr, serum creatinine;BUNcreatinine; BUN, urea nitrogen; KT/V, Urea nitrogen clearance index ; FER, ferritin; hs-CRP, serum hypersensitive C-reactive protein.

Comparison of patients with and without skin pruritus

Compared with uremia patients without skin pruritus, patients with UP had higher levels of PTH, Hb, and hs-CRP. No statistically significant differences were found in demographic characteristics such as sex, age, educational level, BMI, or residence; in levels of Ca, P, ALB, or sCr; or in HD duration (Table 2).

Outcome data

Compared with uremia patients without skin pruritus, patients with UP had higher levels of IPTH, Hb, BUN and hs-CRP. No statistically significant differences were found in demographic characteristics such as Sex, Age, educational level, BMI, Diabetes mellitus status or Place of residence; in levels of Ca, P, ALB, FER, KT/V, or sCr; or in HD duration (Table 2).

Formatted: Tab stops: Not at 0.2"

Table 2. Demographic and clinical characteristics of patients with and without uremic pruritus.

Variable	Pruritic group (n = 60)	Non-pruritic group (n = 88)	*P*
Sex			0.318
Male	39 (65%)	50 (56.8%)	
Female	21 (35%)	38 (43.2%)	
Age, years			0.083
<65 years	45 (75%)	54 (61.4%)	
≥65 years	15 (15%)	34 (38.6%)	
Educational level			0.623
Illiterate or semi-literate	7 (11.7%)	16 (18.2%)	
Primary school	13 (21.7%)	23 (26.2%)	
Junior high school	28 (46.7%)	26 (29.5%)	
High school	8 (13.3%)	11 (12.5%)	
University and above	4 (6.7%)	12 (13.6%)	
Place of residence			0.602
Rural	42 (70%)	58 (65.9%)	
Urban	18 (30%)	30 (34.1%)	
BMI, kg/m^2			0.079~~51~~
≦18.4	13 (21.7%)	12 (13.6%)	
18.5–23.9	41 (68.3%)	60~~58~~ (68.2~~65.9~~%)	
24–27.9	5 (8.3%)	10~~12~~ (11.4~~3.6~~%)	
≥28	1 (1.7%)	6 (6.8%)	
Diabetes mellitus			0.099
Yes	10 (16.7%)	25 (28.4%)	
No	50 (83.3%)	63 (71.6%)	
Ca, mmol/l	2.24 (2.13–2.36~~5~~)	2.19 (2.09–2.34)	0.211
P, mmol/l	1.85 (1.50~~1~~–2.34~~2~~)	1.72 (1.44~~5~~–2.15~~4~~)	0.117
iPTH, pg/ml	368.00 (229~~0~~.75~~50~~–754.25~~746.50~~)	220.50 (111.75~~50~~–503~~2~~.25~~50~~)	0.001*
Hb, g/L	104.00 (98.25~~50~~–111.75~~50~~)	100.00 (91.00–109.00)	0.025*
ALB, g/L	38.10 (35.13~~5~~–40.00)	38.45 (35.80–40.08~~5~~)	0.972
sCr, µmol/L	853.00 (686.00–950.00)	824.00 (629~~34~~.25~~50~~–899.75~~50~~)	0.420

BUN, mmol/L	19.95（18.28-21.65）	19.10（17.60-20.38）	0.028*
KT/V	1.27（1.24-1.31）	1.27（1.22-1.32）	0.567
FER, ng/ml	200.20（127.58-218.25）	190.50（129.63-221.50）	0.696
hs-CRP, mg/L	2.00 (1.00~~5~~–3.00~~6~~)	0.90 (0.50–1.50)	<0.001*
Duration of HD, months	45.50 (18.00–77~~6~~.00)	42.00 (20.25~~50~~–78.75~~77.50~~)	0.936

Notes: The above numerical variables are all non-normal distribution data, articulated by median (range). Intergroup comparisons of variables were made using the Mann–Whitney *U* test. Categorical variables are expressed as counts (percentages). Unordered categorical variables were tested by chi-square; ordered categorical variables such as BMI and educational level were tested by Kruskal–Wallis *H*. BMI, body mass index; Ca, serum calcium; P, serum phosphorus; iPTH, intact~~serum~~ parathyroid hormone; Hb, hemoglobin; ALB, serum albumin; sCr, serum creatinine; BUN, urea nitrogen；KT/V, Urea nitrogen clearance index; FER, ferritin; hs-CRP, serum hypersensitive C-reactive protein.**P* < 0.05.

~~**Comparison of patients with different degrees of skin itching**~~

~~The composition ratio showed a difference between rural and urban patients with varying degrees of skin itching (*P* = 0.017). Differences in PTH and hs-CRP levels of patients with varying degrees of skin itching were statistically significant (*P* = 0.015 and <0.001, respectively), As shown in Table 3. Further comparison between the two groups found that compared with mild skin itching, the proportion of rural residents with moderate skin itching was significantly higher than that of urban residents (*P* = 0.004). Levels of hs-CRP in patients with moderate and severe skin itching were statistically significantly higher than in patients without itching (all *P* <0.001); the level of hs-CRP in patients with severe itching was higher than in those with mild skin itching, to a statistically significant degree (*P* = 0.007). There was no statistically significant difference in PTH levels in patients with severe itching (all *P* > 0.05), As shown in S1 Fig.~~

The composition ratio showed a difference between rural and urban patients with varying degrees of skin itching (*P* = 0.017). Differences in IPTH and hs-CRP levels of patients with different degrees of skin itching were statistically significant (*P* = 0.009 and <0.001, respectively), As shown in Table 3.

Formatted: Indent: First line: 0 ch

Table 3. Baseline characteristics of patients with varying degrees of skin itching.

Formatted: Comment Text

Variable	No itching (n = 88)	Mild itching (n = 22)	Moderate itching (n = 30)	Severe itching (n = 8)	*P*
Sex					0.495
Male	50 (56.8%)	16 (72.7%)	19 (63.3)	4 (50%)	~~0.495~~
Female	38 (43.2%)	6 (27.3%)	11 (36.7)	4 (50%)	

Formatted Table

Formatted: Justified, Indent: First line: 2 ch, Position: Horizontal: 0", Relative to: Column, Vertical: 0", Relative to: Paragraph, Horizontal: 0.13", Wrap Around

Age, years					0.262
<65	54 (61. 4%)	15 (68.2%)	24 (80.0%)	6 (75.0%)	
≥65	34 (38.6%)	7 (31.8%)	6 (20.0%)	2 (25.0%)	0.262
Educational level					0.478
Illiterate/semi-literate	16 (18.2%)	2 (9.1%)	4 (13.3%)	1 (12.5%)	
Primary school	23 (26.1)	3 (13.6%)	8 (26.7%)	2 (25%)	
Junior high school	26 (29. 5%)	11 (50.0%)	12 (40.0%)	5 (62.5%)	0.478
High school	11 (12.5%)	2 (9.1%)	6 (20.0%)	0 (0.0%)	
University and above	12 (13.6%)	4 (18.2%)	0 (0.0%)	0 (0.0%)	
Place of residence					0.017*
Rural	58 (65.9%)	10 (45.5%)	25 (83.3%)	7 (87.5%)	
Urban	30 (34.1%)	12 (54.5%)	5 (16.7%)	1 (12.5%)	0.017*
BMI, kg/m$^{2)}$					0.300
≤18.4	12 (13.6%)	4 (18.2%)	6 (20.0%)	3 (37.5%)	
18.5–23.9	6058 (68.265.9%)	16 (72.8%)	21 (70.0%)	4 (50.0%)	0.226
24–27.9	1012 (11.413.6%)	1 (4.5%)	3 (10.0%)	1 (12.5%)	
≥28	6 (6.86.9%)	1 (4.5%)	0 (0.0%)	0 (0.0%)	
Diabetes mellitus					0.326
Yes	25 (28.4%)	4 (18.2%)	4 (13.3%)	2 (25%)	
No	63 (71.6%)	18 (81.8%)	26 (86.7%)	6 (75%)	
Ca, mmol/L	2.19 (2.09–2.34)	2.25 (2.1516–2.3331)	2.24 (2.1213–2.45)	2.17 (2.0508–2.3128)	0.485

Formatted Table

Formatted Table

P, mmol/L	1.72 (1.44~~45~~–2.15~~14~~)	1.90~~89~~ (1.50~~56~~, 2.47)	1.90~~89~~ (1.62~~63~~, 2.25~~24~~)	1.49 (1.30~~33~~–2.26~~18~~)	0.227
iPTH, pg/mL	220.50 (111.75~~50~~–503.25~~502.50~~)	377~~69~~.50 (280~~68~~.00–719~~5~~.00)	360.00 (207~~9~~.00–793.34~~77.15~~)	497.50 (229.50~~249.00~~–801.25~~774.50~~)	0.009~~0.015~~*
Hb, g/L	100.00 (91.00–109.00)	103.50 (98~~9~~.75~~00~~–109.25~~00~~)	105.00 (98~~9~~.75~~00~~–109~~16~~.25~~00~~)	107.00 (93.25~~94.50~~–118.75~~50~~)	0.138
ALB, g/L	38.45 (35.80–40.08~~5~~)	38.05 (36.68~~80~~–40.85~~0~~)	38.25 (34.73~~90~~–39.63~~0~~)	36.60 (31.73~~85~~–39.83~~65~~)	0.472
sCr, µmol/L	824.00 (629.25~~634.50~~–899.75~~50~~)	853.00 (710~~23~~.00–974.75~~3.00~~)	850.50 (709~~23~~.50~~00~~–955~~953~~.00)	788.50 (422.75~~458.50~~–905~~0~~.00)	0.565
BUN, mmol/L	19.10（17.60-20.38）	19.90（18.18-22.43）	19.80（18.18-21.20）	20.35（19.43-21.73）	0.118
KT/V	1.27（1.22-1.32）	1.28（1.26-1.33）	1.27（1.23-1.31）	1.27（1.22-1.33）	0.708
FER, ng/ml	190.50（129.63-221.50）	203.00（116.50-231.00）	200.50（142.26-206.00）	153.75（109.25-203.85）	0.628
hs-CRP, mg/L	0.90 (0.50–1.50)	1.08 (0.50–2.63~~50~~)	2.15 (1.18~~20~~–2.93~~0~~)	3.93 (2.83~~3.50~~–5.18~~05~~)	<0.001*
HD duration, months	42.00 (20.25~~50~~–78~~7~~.75~~50~~)	38.50 (17.25~~18.00~~–83.25~~00~~)	37.00 (14~~15~~.00–68.25~~67.00~~)	67.00 (56~~9~~.00–89.25~~88. 50~~)	0.119

Notes: P, Hb, ALB, Ca, PTH, sCr, hs-CRP, and HD duration are all non-normal data, articulated by median (range); the rest are conveyed by n (%). Kruskal–Wallis probability between the four groups, with subsequent multiple Mann–Whitney *U* test if significant. Categorical variables are expressed as counts (percentages), unordered categorical variables were tested by chi-square, and ordered categorical variables were tested by Kruskal–Wallis *H*. BMI, body mass index; Ca, serum calcium; P, serum phosphorus; iPTH, intact~~serum~~ parathyroid hormone; Hb, hemoglobin; ALB, serum albumin; sCr, serum creatinine; BUN, urea nitrogen；KT/V, Urea nitrogen clearance index；FER, ferritin; Hb, Hemoglobin; ALB, serum albumin; hs-CRP, serum hypersensitive C-reactive protein; HD, hemodialysis.*$P < 0.05$.

Formatted Table

Formatted Table

Main results

Degree of skin itching in patients with uremic pruritus as analyzed by the single-factor logistic-regression model

Compared with patients without uremic pruritus, Mild itching (OR = 1.917~~36~~; *P* = 0.010~~09~~), moderate itching (OR = 2.696~~2.760~~, P < 0.001), and severe itching (OR = 6.915~~7.086~~; *P* < 0.001) were all related to hs-CRP level. The h~~H~~igher hs-CRP level was closely related to severe itching. iPTH was weakly associated with severe itching (OR = 1.001, *P* = 0.031~~3~~). Hb was slightly associated with moderate itching (OR = 1.036, *P* = 0.026) and severe itching(OR=1.036,P=0.170)~~. There was no evidence that P and Place of residence was associated with severity of skin itching~~, as shown in (Fig 2, S1 Table).

Fig 2. Single-factor logistic-regression analysis of degree of skin itching in patients with uremic pruritus. the error bars represent the lower and upper limits of the 95% confidence interval. the symbol is a circle for mild itching, a triangle for moderate itching, and a square for severe itching. The dashed line in the figure indicates that the odds ratio is equal to 1.

Degree of skin itching in patients with uremic pruritus as analyzed by multivariate logistic regression

We included four variables (hs-CRP, Hb, PTH, ~~P,~~Place of residence) in multiple logistic-regression analysis. Using no itching as a ~~reference,the~~reference, the results showed that risk of skin itching increased with level of hs-CRP (mild skin itching, OR = 1.740~~20~~; 95% CI, 1.061~~44~~–2.84~~3~~5; *P* = 0.028~~33~~; moderate skin itching, OR = 2.838~~84~~; 95% CI, 1.744~~764~~–4.618~~716~~; *P* < 0.001; severe skin itching, OR = 9.440~~8.929~~; 95% CI, 3.547~~340~~–25.124~~23.865~~; *P* < 0.001) . Compared with urban residents, rural residents have a higher risk of moderate itching (OR = 3.869~~942~~; 95% CI, 1.099~~105~~–13.622~~14.069~~; *P*=0.035). as shown in (Fig 3, S2 Table).

Fig 3. Multiple logistic-regression analysis of factors influencing skin itching in patients with uremic pruritus. ~~PTH, Hb, hs-CRP were used as covariates, and Age and pPlace of residence were independent variables for regression analysis.~~ the error bars represent the lower and upper limits of the 95% confidence interval. the symbol is a circle for mild itching, a triangle for moderate itching, and a square for severe itching. The dashed line in the figure indicates that the odds ratio is equal to 1.

Other analyses

Levels of hs-CRP in patients with moderate and severe skin itching were statistically significantly higher than in patients without Itching (all *P* <0.001); the level of iPTH in patients with mild Itching was higher than in those with no skin itching, to a statistically significant degree (*P* <0.05). There was no statistically significant difference in Place of residence in patients with mild, moderate and severe Itching compared with no skin itching (all *P* > 0.05), As shown in Fig 4.

Fig 4. Comparison of patients with varying degrees of skin itching and varying hs-CRP and IPTH levels. (A) Intergroup comparison of hs-CRP levels; **(B)** intergroup comparison of iPTH levels; **(C)** intergroup comparison of the composition ratio of rural to urban residents. *****P* < 0.0001, **P* < 0.05. Error line represents the minimum and maximum values.

Formatted: Font: 14 pt

Formatted: Font: 14 pt

Formatted: Font: 14 pt

Discussion

Key results

In this study, we found that among 148 eligible patients receiving maintenance HD, 60 had UP (prevalence rate, 40.54%); they included 22 mild cases of skin itching (14.86%), 30 moderate cases (20.27%), and 8 severe cases (5.41%). Compared with patients with no skin pruritus, patients with UP had higher levels of iPTH, Hb, BUN and hs-CRP. The composition ratio of rural to urban patients, levels of IPTH and hs-CRP were statistically significantly different in patients with varying degrees of skin pruritus. ~~The proportion of rural residents with severe skin itching was significantly higher than that of urban residents, and there were statistically significant differences in levels of PTH and hs-CRP in patients with different degrees of skin itching.~~ Single-factor logistic-regression analysis discovered a link between hs-CRP levels and all three degrees of itching. Moreover, Hemoglobin is associated with moderate and severe Itching, and iPTH is related to severe Itching. Multi-factor logistic regression analysis also indicated that patients with higher hs-CRP levels had higher risks of mild, moderate, and severe skin itching. Interestingly, the multivariate analysis also showed that rural residents have a higher risk of moderate skin itching than urban residents. ~~Therefore, hs-CRP levels were associated with the intensity of skin itching in patients receiving maintenance HD, and higher hs-CRP levels were associated with severe itching.~~

Limitations

Our study had some limitations. Firstly, it was a single-centre, cross-sectional study that did not show a causal relationship between severity of skin itching in UP and hs-CRP, so further validation is needed from multicentre, large-sample, prospective cohort studies. Secondly, this study did not include some of the influences known in the literature such as normalized protein catabolic rate (nPCR), diet, insulin resistance, interleukins 6 and 2, and κ opioid receptor distribution. Thirdly, the number of patients with severe pruritus is small (n = 8), so readers may not understand a meaningful association with severe pruritus. Finally, the sample size of this study was small and uneven between groups.

Interpretation

This study showed that median hs-CRP level gradually increased along with degree of skin itching in patients with uremic pruritus (no itching, 0.90; mild skin itching, 1.08; moderate skin itching, 2.15; severe skin itching, 3.93). Furthermore, hs-CRP levels in patients with moderate and severe skin itching were significantly higher than in those without itching. These findings suggested that hs-CRP levels might be associated with severity of skin itching. Jiang et al. [7~~11~~] compared the clinical effects of high-throughput hemodialysis (HTH) and hemodialysis filtration (HDF) treat patients with end-stage nephropathy and explored possible potential mechanisms. Their results showed that levels of C-reactive protein (CRP) and leukocyte interleukin-6 (IL-6) in patients receiving HTH significantly improved, compared with those in patients who received HDF. The study also found that HTH had an exact therapeutic effect in UP that was related to inhibition of microinflammation in dialysis patients. Another study also showed that HD patients with UP had significantly higher serum CRP and IL-6 levels compared to HD patients without UP, UP seems to be associated with an up-regulation of micro-

Formatted: Font: 18 pt

Formatted: Space Before: 0 pt

Formatted: Font: 18 pt

Formatted: Indent: First line: 0 ch

inflammation in uremia [8]. Mahmudpour's team[912] indicated that the average reduction in VAS scores in the montelukast group was greater than that in the placebo group, and average levels of hs-CRP in this group showed a downward trend; however, average hs-CRP levels in the placebo group gradually increased, showing that the effect of montelukast treatment in UP might be related to a reduction in an inflammatory response. A factor analysis also shows a close correlation between UP and levels of the inflammatory factors CRP and leukocyte IL-6[1013]. Based on this study and previous studies, inflammatory responses appear to play an important role in UP.

We analyzed the factors affecting degree of skin itching in UP patients and then corrected for patients' levels of Hb and PTH, Place of residence, P. Multi-factor logistic-regression analysis found that hs-CRP levels were associated with severity of skin itching and were a risk factor. Compared with patients without itching, the OR value for mild skin itching was 1.74020, that for moderate skin itching was 2.83884, and that for severe skin itching was 9.4408.929, indicating that degree of skin itching increased along with hs-CRP levels. A previous multivariable logistic-regression analysis confirmed that older age and higher CRP levels are associated with severe itching [3]., Still,but in our study, neither single-factor nor multi-factor logistic-regression analysis showed any differences in skin itching between elderly and non-elderly patients. In our study, the OR value for hs-CRP of patients with severe skin itching was significantly higher than that of patients without itching, suggesting that higher hs-CRP levels were closely associated with severe skin itching, which was consistent with the results of an earlier study. Pakfetrat et al. [1114] assessed the effect of sertraline on UP. patients were randomly divided into two groups, the trial group receiving sertraline and the control group taking a placebo, and their severity of itching was measured by two scoring systems (visual-simulation score and DUO scoring system [1215]). The researchers found that differences in skin itching intensity between the two scoring systems and between the two groups were directly related to CRP level, and they believed that sertraline reduced skin itching, perhaps due to its effect of reducing the number of inflammatory cytokines. Tseng et al.[1316]studying a possible association between vegetarians and UP, found that levels of inflammatory factors such as leukocyte IL-2 and hs-CRP, visual-simulation score (Analog Score and VAS), and itching score were also lower in vegetarian patients than in non-vegetarian ones; this demonstrated that a vegetarian diet might be associated with amelioration of uremic-pruritus severity in hemodialysis patients.

In our study, the composition ratio showed a significant difference between rural and urban patients with different degrees of skin itching. Compared with patients without itching, the proportion of rural patients with moderate and severe skin itching was relatively high, however, no statistically significant difference was achieved. Although the single factor analysis did not indicate that the place of residence was related to the intensity of the patient's skin itching, the multi-factor analysis demonstrated that rural residents had a higher risk of moderate skin itching than urban residents. The possible reason is that on the one hand, compared with urban residents, rural residents have lower living habits and lower health awareness; on the other hand, with the development of urbanization, rural areastheareas the air pollution in Xinchang's local rural areas is gradually increasing and the urban environment is slowlygradually improving. UP in patients receiving maintenance HD is associated with air pollutants such as NO2 and CO [1417]. , and aA cross-sectional study suggested that UP in patients receiving maintenance HD might be associated with the number of days in which environmental particulate matter ≤2.5 μ in diameter (PM2.5) exceeds standard levels [1518]. The above mentioned two studies suggest that itching of the skin might be associated with the patient's living environment. In this study, there were a total of 148 patients with uremia and eight patients

Formatted: Font: 10 pt

Formatted: Not Superscript/ Subscript

Formatted: Font: 10 pt

Formatted: Not Superscript/ Subscript

Formatted: Font: (Default) Calibri, 10 pt

with severe skin pruritus, accounting for 5.4% of the total patients.2012-2015 DPPSO study [3] surveyed 7,629 people, divided into five groups (not at all, somewhat, moderately, Very Much, Extremely) according to the degree of itchy skin. , Eextreme Itching accounted for 7%, which is slightly higher than 5.4% of patients with severe Itching in this study. The reason for the differences may lie in the different criteria used to assess the degree of pruritus in the patient's skin.equivalent to 5.4% of patients with severe Itching in this study. Multicenter, extensive sample studies may increase the proportion of patients with severe Itching. therefore, further studies are needed to expand the sample and detect the link between the degree of skin itch and air pollutant levels in patients of urban and rural areas.

In this study, the ratio of rural residents to urban residents in patients with severe itching was higher than that in patients with moderate itching, but the results of multivariate analysis did not show that rural residents had a higher risk of severe itching than urban residents. The possible reason lies in the patients with severe itching in this study were less relevant, only 8 cases, and 30 patients with moderate itching. therefore, further studies are needed to expand the sample and detect air pollutant levels in urban and rural areas.

iPTH levels differed between patients with varying degrees of skin itching. Median iPTH level in patients with severe skin itching was significantly higherwas higher than that in patients without itching. Still,, but there was no statistically significant difference between iPTH levels across groups, and single-factor regression analysis suggested a weak association between higher iPTH levels and severe skin itching. However, after we corrected for hs-CRP and Hb levels, P, andPlace of residence, that association disappeared. After parathyroid excision, iPTH level and serum phosphate concentration are significantly reduced, and skin itching disappears [16,1719, 20]. A study by Moldovan et al. [1821] suggests that the average value of PTH decreased significantly after parathyroidectomy, and the symptoms of bone pain were significantly improved. However, muscle weakness and itching of the skin did not improve. Levy et al. [1922] studied the associated symptoms of sHPT in patients receiving maintenance HD. Their results showed that an increase in PTH levels was associated with skin itching worsening over time, and the effect was more obvious with larger changes in PTH level. However, the combination of the disease, detection of medication, inclusion of up to 19 symptoms, the larger *P*-value threshold (0.1), and other promiscuous factors might have affected the reliability of that study.

SomeOther studies suggested that UP is related to Ca, P, ALB, and uremic toxins [11,2014, 23]. Nevertheless, contradictory data on the impact of Ca and P have been reported [21]. In this study, no statistically significant differences were found in Ca, P, ALB, and creatinine when comparing groups of patients with different degrees of skin pruritus. There may indeed be a lack of test power, which is considered to be related to the small sample size of this study and the unbalanced sample size among the groups. However, single-factor logistic regression analysis also did not find an association between these factors and the degree of skin pruritus. Therefore, these factors were ultimately not included in the multifactor logistic regression analysis. In fact, despite various substances have been discussed as potential pruritogens in chronic renal diseases, a causal relationship could never be established[22].We did not find an association between sCR, BUN, ALB and uremic pruritus because there were no statistical differences in comparisons between groups of patients with different degrees of skin pruritus. Besides, a single factor analysis did not reveal an association between these factors and the degree of pruritus. In fact, despite various substances have been discussed as potential pruritogens in chronic renal diseases, a causal relationship could never be established [22]. Our

Formatted: Font: (Default) Calibri

Formatted: Font: (Default) Calibri, 10 pt

Formatted: Normal (Web), Indent: First line: 1.42 ch

Formatted: Font: (Default) Calibri

Formatted: Font: (Default) Calibri, 10 pt, Font color: Auto

Formatted: Font: (Default) Calibri

~~research showed that compared with patients without pruritus, the levels of Ca, P, ALB, and sCr in pruritus patients were not statistically abnormal. ALB, and sCr levels were not associated with UP.~~

At present, only a few studies have explored the factors affecting skin itching intensity in these patients. Wieczorek et al. [23] documented Intensity of skin itching have a significant negative correlation with expression of κ opioid receptors in patients with HD. Also, hs-CRP levels, male sex, HD duration, insulin resistance, insufficient dialysis adequacy, and hyperphosphatemia are positively correlated with UP intensity [24], increasing the blood flow of the HD machine can reduce the intensity and frequency of skin itching [25]. However, the above preliminary research used visual-analogue scoring (VAS) to evaluate the degree of skin itching. In this study, we used a five-dimensional itching scale to evaluate skin itching across multiple dimensions in uremic patients undergoing maintenance HD. Although VAS is sufficient to assess the severity of symptoms, it does not take into account other aspects of pruritus, such as the relative impact of pruritus on quality of life, changes in pruritus over time, and the location of pruritus. The 5-D score was strongly correlated with the numerical rating scale（NRS）and VAS [5, 26], which is a questionnaire with acceptable validity, reliability and sensitivity to change to evaluate pruritus in Thai and Arabic patients [26, 27]. The 5-dimensional Pruritus Scale is recommended to assess the severity of pruritus because it is more accurate than VAS and more sensitive to the multidimensional nature of pruritus [28].

Formatted: Indent: First line: 1.35 ch, Widow/Orphan control, Adjust space between Latin and Asian text, Adjust space between Asian text and numbers

Generalizability

In conclusion, our study suggested that the intensity of skin itching in patients with UP was associated with hs-CRP and that higher hs-CRP levels were associated with severe skin itching. Thus, this study demonstrates that controlling inflammation can relieve itchy skin in patients with uremia, and is particularly clinically relevant in patients with severe itchy skin. Besides, the environment may be related to the degree of itchy skin in uremia patients. ~~Overall, our study suggested that the intensity of skin itching in patients with UP was associated with hs-CRP and that higher hs-CRP levels were associated with severe skin itching. The limitation of this study is that it was a single-center, cross-sectional study that did not show a causal relationship between severity of skin itching in UP and hs-CRP, so further validation is needed from multi-center, large-sample, prospective cohort studies.~~

Formatted: Indent: First line: 0 ch

Supporting information

Formatted: Font: 18 pt

~~**S1 Fig. Comparison of patients with varying degrees of skin itching and varying hs-CRP and PTH levels. (A)** Intergroup comparison of hs-CRP levels; **(B)** intergroup comparison of PTH levels; **(C)** intergroup comparison of the composition ratio of rural to urban residents. ****P < 0.0001, **P < 0.01. Error line represents minimum and maximum values.~~

S1 Table. Single-factor logistic-regression analysis of degree of skin itching in patients with uremic pruritus.

S2 Table. Multiple logistic-regression analysis of factors influencing skin itching in patients with uremic pruritus~~, using hs-CRP as a variable~~.

Acknowledgements

Formatted: Font: 18 pt

We thank LetPub (www.letpub.com) for its linguistic assistance during the preparation of this

manuscript.

Author contributions

Conceptualization: Jian-Hui Zhao, Yi-Wen Li.
Data curation: Qing-Shuang Zhu, Li-Li Wang.
Investigation: Jian-Hui Zhao, Yi-Wen Li, Qing-Shuang Zhu.
Methodology: Jian-Hui Zhao, Yi-Wen Li.
Project administration: Jian-Hui Zhao.
Supervision: Yi-Wen Li.
Writing – original draft: Jian-Hui Zhao.
Writing – review and editing: Jian-Hui Zhao, Yi-Wen Li, Qing-Shuang Zhu.

Funding~~:~~

The authors received no specific funding for this work.

Formatted: Font: 18 pt

Formatted: Font: 18 pt

Formatted: Font: 18 pt

References

1. Kfoury LW, Jurdi MA. Uremic pruritus. J Nephrol. 2012;25(5):644-52. Epub 2011/10/11. doi: 10.5301/jn.5000039. PubMed PMID: 21983988.

2. Combs SA, Teixeira JP, Germain MJ. Pruritus in Kidney Disease. Semin Nephrol. 2015;35(4):383-91. Epub 2015/09/12. doi: 10.1016/j.semnephrol.2015.06.009. PubMed PMID: 26355256; PubMed Central PMCID: PMCPMC5497472.

3. Rayner HC, Larkina M, Wang M, Graham-Brown M, van der Veer SN, Ecder T, et al. International Comparisons of Prevalence, Awareness, and Treatment of Pruritus in People on Hemodialysis. Clin J Am Soc Nephrol. 2017;12(12):2000-7. Epub 2017/09/20. doi: 10.2215/CJN.03280317. PubMed PMID: 28923831; PubMed Central PMCID: PMCPMC5718267.

4. Grochulska K, Ofenloch RF, Mettang T, Weisshaar E. Mortality of Haemodialysis Patients ~~With~~with and Without Chronic Itch: A Follow-up Study of the German Epidemiological Hemodialysis Itch Study (GEHIS). Acta Derm Venereol. 2019;99(4):423-8. Epub 2019/01/24. doi: 10.2340/00015555-3125. PubMed PMID: 30673111.

5. Elman S, Hynan LS, Gabriel V, Mayo MJ. The 5-D itch scale: a new measure of pruritus. Br J Dermatol. 2010; 162:587-93. https://doi.org/10.1111/j.1365-2133.2009.09586.x PMID: 19995367

~~Wu HY, Peng YS, Chen HY, Tsai WC, Yang JY, Hsu SP, et al. A Comparison of Uremic Pruritus in Patients Receiving Peritoneal Dialysis and Hemodialysis. Medicine (Baltimore). 2016;95(9):e2935. Epub 2016/03/06. doi: 10.1097/md.0000000000002935. PubMed PMID: 26945400; PubMed Central PMCID: PMCPMC4782884.~~

6. Keithi-Reddy SR, Patel TV, Armstrong AW, Singh AK. Uremic pruritus. Kidney Int. 2007; 72:373-7. https://doi.org/10.1038/sj.ki.5002197 PMID: 17429345

~~Wieczorek A, Krajewski P, Kozioł-Gałczyńska M, Szepietowski JC. Opioid receptors expression in the skin of haemodialysis patients suffering from uraemic pruritus. J Eur Acad Dermatol Venereol. 2020. Epub 2020/03/15. doi: 10.1111/jdv.16360. PubMed PMID: 32170896.~~

7. Jiang X, Ji F, Chen ZW, Huang QL. Comparison of high-flux hemodialysis with hemodialysis filtration in treatment of uraemic pruritus: a randomized controlled trial. Int Urol Nephrol. 2016; 48:1533-41. https://doi.org/10.1007/s11255-016-1364-2 PMID: 27379625

~~Abdelsalam M, Tawfik M, Reda EM, Eldeeb AA, Abdelwahab A, Zaki ME, et al. Insulin Resistance and Hepatitis C Virus-Associated Subclinical Inflammation Are Hidden Causes of Pruritus in Egyptian Hemodialysis Patients: A Multicenter Prospective Observational Study. Nephron. 2019;143(2):120-7. Epub 2019/07/17. doi: 10.1159/000501409. PubMed PMID: 31311030.~~

8. Kimmel M, Alscher DM, Dunst R, Braun N, Machleidt C, Kiefer T, et al. The role of micro-

Formatted: Indent: Left: 0", First line: 0 ch

inflammation in the pathogenesis of uraemic pruritus in haemodialysis patients. Nephrol Dial Transplant. 2006; 21:749-55. https://doi.org/10.1093/ndt/gfi204 PMID: 16249205

Aliasgharpour M, Zabolypour S, Asadinoghabi A, Haghani H, Lesanpezeshki M. The Effect of Increasing Blood Flow Rate on Severity of Uremic Pruritus in Hemodialysis Patients: A Single Clinical Trial. J Natl Med Assoc. 2018;110(3):270-5. Epub 2018/05/21. doi: 10.1016/j.jnma.2017.04.008. PubMed PMID: 29778130.

9. Mahmudpour M, Roozbeh J, Raiss Jalali GA, Pakfetrat M, Ezzat Zadegan S, Sagheb MM. Therapeutic Effect of Montelukast for Treatment of Uremic Pruritus in Hemodialysis Patients. Iran J Kidney Dis. 2017; 11:50-5. PMID: 28174353

Elman S, Hynan LS, Gabriel V, Mayo MJ. The 5-D itch scale: a new measure of pruritus. Br J Dermatol. 2010;162(3):587-93. Epub 2009/12/10. doi: 10.1111/j.1365-2133.2009.09586.x. PubMed PMID: 19995367; PubMed Central PMCID: PMCPMC2875190.

10. Schricker S, Heider T, Schanz M, Dippon J, Alscher MD, Weiss H, et al. Strong Associations Between Inflammation, Pruritus and Mental Health in Dialysis Patients. Acta Derm Venereol. 2019; 99:524-9. https://doi.org/10.2340/00015555-3128 PMID: 30673107

Keithi-Reddy SR, Patel TV, Armstrong AW, Singh AK. Uremic pruritus. Kidney Int. 2007;72(3):373-7. Epub 2007/04/13. doi: 10.1038/sj.ki.5002197. PubMed PMID: 17429345.

11. Pakfetrat M, Malekmakan L, Hashemi N, Tadayon T. Sertraline can reduce uremic pruritus in hemodialysis patient: A double blind randomized clinical trial from Southern Iran. Hemodial Int. 2018; 22:103-9. https://doi.org/10.1111/hdi.12540 PMID: 28263039

Jiang X, Ji F, Chen ZW, Huang QL. Comparison of high-flux hemodialysis with hemodialysis filtration in treatment of uraemic pruritus: a randomized controlled trial. Int Urol Nephrol.

2016;48(9):1533-41. Epub 2016/07/06. doi: 10.1007/s11255-016-1364-2. PubMed PMID: 27379625.

12. Duo LJ. Electrical needle therapy of uremic pruritus. Nephron. 1987; 47:179-83. https://doi.org/10.1159/000184487 PMID: 3500424

13. Tseng CY, Wu TT, Lai CW, Lin HJ, Chou CY, Chang CT, et al. Vegetarian diet may ameliorate uremic pruritus in hemodialysis patients. Ren Fail. 2018; 40:514-9. https://doi.org/10.1080/0886022X.2018.1512871 PMID: 30396306

14. Huang W, Lin J, Weng C, Hsu C, Yen T. Environmental NO 2 and CO Exposure: Ignored Factors Associated with Uremic Pruritus in Patients Undergoing Hemodialysis. Sci Rep. 2016; 6:1-10.

15. Liu MH, Chan MJ, Hsu CW, Weng CH, Yen TH, Huang WH. Association of uremic pruritus in hemodialysis patients with the number of days of high mean 24-hour particulate matter with a diameter of <2.5 mum. Ther Clin Risk Manag. 2017; 13:255-62. https://doi.org/10.2147/TCRM.S129133 PMID: 28260912

16. Jing Y, Zhao H, Ge Y, Jia F, He Q, Wang S, et al. Application of total parathyroidectomy with auto-transplantation for uremia secondary hyperparathyroidism treatment. Int J Clin Exp Med. 2015; 8:11188.

17. Zhang Y, Lu Y, Feng S, Zhan Z, Shen H. Evaluation of laboratory parameters and symptoms after parathyroidectomy in dialysis patients with secondary hyperparathyroidism. Ren Fail. 2019; 41:921-9. https://doi.org/10.1080/0886022X.2019.1666724 PMID: 31573378

18. Moldovan D, Racasan S, Kacso IM, Rusu C, Potra A, Bondor C, et al. Survival after parathyroidectomy in chronic hemodialysis patients with severe secondary

hyperparathyroidism. Int Urol Nephrol. 2015; 47:1871-7. https://doi.org/10.1007/s11255-015-1106-x PMID: 26377492

19. Levy AR, Xing S, Brunelli SM, Cooper K, Finkelstein FO, Germain MJ, et al. Symptoms of Secondary Hyperparathyroidism in Patients Receiving Maintenance Hemodialysis: A Prospective Cohort Study. Am J Kidney Dis. 2020; 75:373-83.

20. Momose A, Shiraiwa Y, Narita S, Kusumi T, Goto S, Sera K. Total Calcium and Albumin Are Decreased in the Deeper Epidermis of Patients with Chronic Kidney Disease-Associated Pruritus. Nephron. 2017; 136:103-10. https://doi.org/10.1159/000458417 PMID: 28183080

21. Shirazian S, Kline M, Sakhiya V, Schanler M, Moledina D, Patel C, et al. Longitudinal predictors of uremic pruritus. J Ren Nutr. 2013; 23:428-31. https://doi.org/10.1053/j.jrn.2013.08.002 PMID: 24209894

22. Mettang T, Kremer AE. Uremic pruritus. Kidney Int. 2015; 87:685-91. https://doi.org/10.1038/ki.2013.454

23. Wieczorek A, Krajewski P, Kozioł-Gałczyńska M, Szepietowski JC. Opioid receptors expression in the skin of haemodialysis patients suffering from uraemic pruritus. J Eur Acad Dermatol Venereol. ~~2020 .~~2020. https://doi.org/10.1111/jdv.16360 PMID: 32170896

24. Abdelsalam M, Tawfik M, Reda EM, Eldeeb AA, Abdelwahab A, Zaki ME, et al. Insulin Resistance and Hepatitis C Virus-Associated Subclinical Inflammation Are Hidden Causes of Pruritus in Egyptian Hemodialysis Patients: A Multicenter Prospective Observational Study. Nephron. 2019; 143:120-7. https://doi.org/10.1159/000501409 PMID: 31311030

25. Aliasgharpour M, Zabolypour S, Asadinoghabi A, Haghani H, Lesanpezeshki M. The Effect of

Formatted: EndNote Bibliography

Formatted: English (United Kingdom)

Formatted: EndNote Bibliography, Indent: Left: 0", First line: 0 ch

Formatted: No underline

Formatted: No underline

Formatted: English (United Kingdom)

Increasing Blood Flow Rate on Severity of Uremic Pruritus in Hemodialysis Patients: A Single Clinical Trial. J Natl Med Assoc. 2018; 110:270-5. https://doi.org/10.1016/j.jnma.2017.04.008 PMID: 29778130

26. Lai JW, Chen HC, Chou CY, Yen HR, Li TC, Sun MF, et al. Transformation of 5-D itch scale and numerical rating scale in chronic hemodialysis patients. BMC Nephrol. 2017; 18:56. https://doi.org/10.1186/s12882-017-0475-z PMID: 28178931

27. Khan TM, Al-Haider I, Syed Sulaiman SA, Hassali MA. Linguistic validation of the 5D itching scale to Arabic in patients with end-stage kidney disease. J Ren Care. 2013; 39:222-7. https://doi.org/10.1111/j.1755-6686.2013.12038.x PMID: 24152068

28. Nofal E, Farag F, Nofal A, Eldesouky F, Alkot R, Abdelkhalik Z. Gabapentin: A promising therapy for uremic pruritus in hemodialysis patients: A randomized-controlled trial and review of literature. J Dermatolog Treat. 2016; 27:515-9. https://doi.org/10.3109/09546634.2016.1161161 PMID: 27043168

Formatted: No underline

Formatted: No underline

Formatted: English (United Kingdom)

Formatted: No underline

Formatted: No underline

Formatted: English (United Kingdom)

Formatted: No underline

Formatted: No underline

Formatted: English (United Kingdom)

Formatted: No underline

Formatted: No underline

Formatted: No underline

Formatted: EndNote Bibliography, Indent: Left: 0", Hanging: 3 ch

Response to the PLOS ONE's editor

Thank you for the hard work of the academic editors of "PLOS ONE". Thank you for accompanying me and helping me grow up. As for the issues that need my attention, I summarize as follows.

1. Please ensure that your manuscript meets PLOS ONE's style requirements, including those for file naming. The PLOS ONE style templates can be found at https://journals.plos.org/plosone/s/file?id=wjVg/PLOSOne_formatting_sample_main_body.pdf and https://journals.plos.org/plosone/s/file?id=ba62/PLOSOne_formatting_sample_title_authors_affiliations.pdf

Response:

We have revised the paper following your journal's formatting requirements for title, authorship, and text.

2.While revising your submission, please upload your figure files to the Preflight Analysis and Conversion Engine (PACE) digital diagnostic tool, https://pacev2.apexcovantage.com/. PACE helps ensure that figures meet PLOS requirements. To use PACE, you must first register as a user. Registration is free. Then, login and navigate to the UPLOAD tab, where you will find detailed instructions on how to use the tool. If you encounter any issues or have any questions when using PACE, please email PLOS at figures@plos.org. Please note that Supporting Information files do not need this step.

Response:

A total of four images are in the paper, and the image files have been uploaded to the PACE software, all with a resolution of 600ppi, 2000-4500 pixels wide and 1358-3925 pixels high, and are currently sealed in the PACE software.

Response to Reviewer

Thank the reviewers for these lovely comments concerning our manuscript entitled "Determinants of the intensity of uremic pruritus in patients receiving maintenance hemodialysis: A cross-sectional study". These comments are all valuable and very helpful for revising and improving our paper, as well as the essential guiding significance to our researches. We have studied comments carefully and have made corrections which we hope to meet with approval. The response to the reviewer's comments are as follows:

Reviewer #1

The authors performed a cross-sectional analysis of 148 hemodialysis patients to identify factors associated with uremic pruritus. They found that higher levels of PTH, hemoglobin, and hs-CRP, as well as the rural residence of patients were associated with worse pruritus. There are major concerns with the lack of dialysis and other pertinent laboratory data. Overall, the results do not add new data to the existing literature.

1. There is no data provided for dialysis adequacy, which has been shown to be associated with pruritus in prior studies. Likewise, BUN levels, nPCR, ferritin levels, and diabetic status should be included in the analysis.

Response:

The revision adds these factors, such as urea nitrogen, ferritin level, diabetes, and single pool KT/V(spKT/V), to the list of factors related to hemodialysis. Normalized protein catabolic rate (nPCR) is a nutritional index for hemodialysis patients. Its measurement requires multiple blood collections, which can be challenging to obtain, especially with the inclusion of serum albumin, which is a nutritional index. The spKT/V index reflects the adequacy of single hemodialysis.

This section is mainly shown on pages 6-11, tables 1-3.

2. The authors used the Five-Dimensional Itching Scale (5DIS) to assess the degree of pruritus in patients, noting that this scale would "produce more objective and comprehensive results" compared to the visual-analogue scoring scale. The authors should provide data to justify this statement.

Response:

In this study, we used a five-dimensional itching scale to evaluate skin itching across multiple dimensions in uremic patients undergoing maintenance HD. Although VAS is sufficient to assess the severity of symptoms, it does not take into account other aspects of pruritus, such as the relative impact of pruritus on quality of life, changes in pruritus over time, and the location of pruritus. The 5-D score was strongly correlated with the numerical rating scale（NRS）and VAS[5, 26], which is a questionnaire with acceptable validity, reliability and sensitivity to change

to evaluate pruritus in Thai and Arabic patients[26, 27]. The 5-dimensional Pruritus Scale is recommended to assess the severity of pruritus because it is more accurate than VAS and more sensitive to the multidimensional nature of pruritus[28].
This section is detailed on page15, line 29-38.

3. A large proportion of the discussion was dedicated to the finding that higher hs-CRP levels were associated with pruritus. A prior study has previously reported this finding, along with levels of cytokines and T cells (PMID 16249205); this reference should be cited.

Response:

This section is detailed on Reference #8（page 13, line35）

4. The number of patients with severe pruritus is small (n=8) and therefore, meaningful associations with severe pruritus may not be appreciated.

Response:

In this study, there were a total of 148 patients with uremia and eight patients with severe skin pruritus, accounting for 5.4% of the total patients. 2012-2015 DPPSO study[3] surveyed 7,629 people, divided into five groups (not at all, somewhat, moderately, Very Much, Extremely) according to the degree of itchy skin. Extreme Itching accounted for 7%, which is slightly higher than 5.4% of patients with severe Itching in this study. The reason for the differences may lie in the different criteria used to assess the degree of pruritus in the patient's skin.

This section is detailed on page 14, lines 37-43

Reviewer #2

In this study, the authors studied uremic pruritus using the five-dimensional itch scale in 148 adult patients of end-stage renal disease receiving hemodialysis and report the prevalence of 40.5% and association with parathormone (PTH), hemoglobin and high-sensitive C reactive protein (hs-CRP) on bivariate analysis and hs-CRP alone on multiple logistic regression. The study seems to be designed logically, conducted scientifically and analyzed with due statistical rigour. However, the manuscript is lengthy, does not have a smooth flow of information and requires rewriting as per the STROBE checklist. Major observations are described below. Minor observations are inserted in the uploaded version of the manuscript as “sticky notes” at appropriate places.

Response:

(1) We have rewritten the paper according to the STROBE checklist, details of which can be found in the attached STROBE checklist.

(2) Concerning some minor observation, we have followed your comments with deep appreciation.

Major observation:

1. The introduction should briefly summarize the present state of knowledge on uremic pruritus, highlight the gap in understanding the study sought to address and state the study's objective. Comparison with previous studies (measurement of severity of Itching) should be reserved for discussion.

Response:

The reviewer's suggestions were excellent, and we have corrected them.

This section has been moved to page 15, lines 23-29.

2. The stated aims of the study are to "1. explore the prevalence of UP in maintenance HD patients and the demographic and clinical characteristics of pruritus patients. Explore determinants of higher pruritus intensity based on participants' clinical and biochemical parameters and their pruritus assessments by 5DIS at baseline". The second aim is somewhat unclear. Were characteristics of patients with higher pruritus intensity to be compared with those of patients with of lower pruritus intensity or with those of patients without pruritus? Clear statement in the PICO format is suggested. What is the novelty of the research question? This needs to be justified.

Response:

objectives

(1). explore the prevalence of UP in maintenance HD patients and the demographic and clinical characteristics of pruritus patients.

(2). analyze the relationship between high-sensitivity C-reactive protein and different degrees of skin itching.

This section has been moved to page 2, lines 28-32.

3. The setting of the study may be described in "Methods".

Response:

This section is detailed on ***Methods (study setting)***, page2, lines 38 & page3, lines 1-5.

4.How were biochemical parameters such as serum calcium, phosphorus, creatinine, etc assayed? The detailed methods need not be described but should be referenced.

Response:

We assayed biochemical indicators such as Ca, P, iPTH, Hb, ALB, sCr, Bun, FER, sp/KTV and hs-CRP. The hospital's central laboratory performed all of the laboratory tests, and auto-analyzers were used to determine biochemical data, iPTH was measured with Roche second-generation assay. The clinical biochemical indicators are based on the data within three months from the survey day. If there is no data within three months, the patient is required to re-test. If the patient is unwilling to cooperate with the test, it is a missing value. spKT/V(single pool KT/V)=$-\ln(R-0.008t)+(4-3.5R)*(\Delta BW/BW)$. Note: K refers to Blood urea clearance rate of dialyzer（L/h）, t is the dialysis time (h), and V is the distribution volume of urea (V). R is the ratio of blood urea nitrogen after dialysis to blood urea nitrogen before dialysis; t is single dialysis time in h; ΔBW is the weight change value before and after dialysis, i.e. ultrafiltration, unit L; BW is weight in kg. Blood collection requirements: blood samples before dialysis from the artery end of the vascular path, after dialysis before blood sample collection to stop ultrafiltration, reduce blood flow of 50 ml/min, wait 15 seconds after blood collection from the artery as a blood sample after dialysis.

This section is detailed on page 3, lines 36-41 & page 4, lines 1-7.

5.The study reports no significant association of uremic pruritus with factors such as calcium, phosphorus, creatinine and albumin. However, the sample size calculation was made to estimate the prevalence of uremic pruritus. Could the study have been under powered for the study of these factors?

Response:

Some studies suggested that UP is related to Ca, P, ALB, and uremic toxins[11, 20]. Nevertheless, contradictory data on the impact of Ca and P have been reported[21]. In this study, no statistically significant differences were found in Ca, P, ALB, and creatinine when comparing groups of patients with different degrees of skin pruritus. There may indeed be a lack of test power（Since the data for these factors are non-normally distributed, the exact distribution is unknown. Based on statistical theory, it is not possible to calculate the true test power with certainty.）, which is considered to be related to the small sample size of this study and the unbalanced sample size among the groups. However, single-factor logistic regression analysis also did not find an association between these factors and the degree of skin pruritus. Therefore, these factors were ultimately not included in the multifactor logistic regression analysis. In fact, despite various substances have been discussed as potential pruritogens in chronic renal diseases, a causal relationship could never be established[22].

This section is detailed on page 15, lines 14-22.

6.The following factors influencing uremic pruritus have not been studied: diet, insulin resistance, dialysis adequacy, interleukins 6 & 2, and κ opioid receptor distribution. These need to be acknowledged as limitations.

Response:

Firstly, this study was a single-center study with a limited number of people on hemodialysis, and therefore the number of people included in the study was limited. Many factors influence skin itching in uremic patients, and it is not practical to include all relevant aspects of the study. Secondly, there is the issue of patient compliance. For example, to study diet factor, we need to distinguish between vegetarian and non-vegan patients, which requires strict control of the patient's diet. For insulin resistance, if the HOMA model is used to assess the resistance of the body to insulin (HOMA-IR), fasting blood glucose and fasting insulin levels need to be measured based on the formula HOMA-IR=FPG×FINS/22.5, therefor, fasting blood needs to be collected before hemodialysis. Some patients are difficult to cooperate, the missing value of the study will be further increased. Thirdly, for dialysis adequacy, we included a urea clearance index (kt/v) to assess this. In conclusion, it is essential to acknowledge the limitations of this study.

This section is detailed on page 13, lines 17-19.
