# Supplementary material for: Determinants of the intensity of uremic pruritus in patients receiving maintenance hemodialysis: A cross-sectional study
Source: PLoS One. 2021 Jan 20;16(1):e0245370. doi: 10.1371/journal.pone.0245370 (PMC7817000; doi:10.1371/journal.pone.0245370)
Supplement: Attachment — Submitted filename: Response to Reviewers.docx [file pone.0245370.s004.docx]

**Response to the PLOS ONE’s editor**

Thank you for the hard work of the academic editors of “PLOS ONE”. Thank you for accompanying me and helping me grow up. As for the issues that need my attention, I summarize as follows.

1. Please ensure that your manuscript meets PLOS ONE's style requirements, including those for file naming. The PLOS ONE style templates can be found at

https://journals.plos.org/plosone/s/file?id=wjVg/PLOSOne_formatting_sample_main_body.pdf and

<https://journals.plos.org/plosone/s/file?id=ba62/PLOSOne_formatting_sample_title_authors_affiliations.pdf>

**Response：**

We have revised the paper following your journal's formatting requirements for title, authorship, and text.

2.While revising your submission, please upload your figure files to the Preflight Analysis and Conversion Engine (PACE) digital diagnostic tool, https://pacev2.apexcovantage.com/. PACE helps ensure that figures meet PLOS requirements. To use PACE, you must first register as a user. Registration is free. Then, login and navigate to the UPLOAD tab, where you will find detailed instructions on how to use the tool. If you encounter any issues or have any questions when using PACE, please email PLOS at figures@plos.org. Please note that Supporting Information files do not need this step.

**Response：**

A total of four images are in the paper, and the image files have been uploaded to the PACE software, all with a resolution of 600ppi, 2000-4500 pixels wide and 1358-3925 pixels high, and are currently sealed in the PACE software.

**Response to Reviewer**

Thank the reviewers for these lovely comments concerning our manuscript entitled “Determinants of the intensity of uremic pruritus in patients receiving maintenance hemodialysis: A cross-sectional study”. These comments are all valuable and very helpful for revising and improving our paper, as well as the essential guiding significance to our researches. We have studied comments carefully and have made corrections which we hope to meet with approval. The response to the reviewer’s comments are as follows:

**Reviewer #1**

The authors performed a cross-sectional analysis of 148 hemodialysis patients to identify factors associated with uremic pruritus. They found that higher levels of PTH, hemoglobin, and hs-CRP, as well as the rural residence of patients were associated with worse pruritus. There are major concerns with the lack of dialysis and other pertinent laboratory data. Overall, the results do not add new data to the existing literature.

1. There is no data provided for dialysis adequacy, which has been shown to be associated with pruritus in prior studies. Likewise, BUN levels, nPCR, ferritin levels, and diabetic status should be included in the analysis.

**Response:**

The revision adds these factors, such as urea nitrogen, ferritin level, diabetes, and single pool KT/V(spKT/V), to the list of factors related to hemodialysis. Normalized protein catabolic rate (nPCR) is a nutritional index for hemodialysis patients. Its measurement requires multiple blood collections, which can be challenging to obtain, especially with the inclusion of serum albumin, which is a nutritional index. The spKT/V index reflects the adequacy of single hemodialysis.

This section is mainly shown on pages 6-11, tables 1-3.

2. The authors used the Five-Dimensional Itching Scale (5DIS) to assess the degree of pruritus in patients, noting that this scale would “produce more objective and comprehensive results” compared to the visual-analogue scoring scale. The authors should provide data to justify this statement.

**Response:**

In this study, we used a five-dimensional itching scale to evaluate skin itching across multiple dimensions in uremic patients undergoing maintenance HD. Although VAS is sufficient to assess the severity of symptoms, it does not take into account other aspects of pruritus, such as the relative impact of pruritus on quality of life, changes in pruritus over time, and the location of pruritus. The 5-D score was strongly correlated with the numerical rating scale（NRS）and VAS[5, 26], which is a questionnaire with acceptable validity, reliability and sensitivity to change to evaluate pruritus in Thai and Arabic patients[26, 27]. The 5-dimensional Pruritus Scale is recommended to assess the severity of pruritus because it is more accurate than VAS and more sensitive to the multidimensional nature of pruritus[28].

This section is detailed on page15, line 29-38.

3. A large proportion of the discussion was dedicated to the finding that higher hs-CRP levels were associated with pruritus. A prior study has previously reported this finding, along with levels of cytokines and T cells (PMID 16249205); this reference should be cited.

**Response：**

This section is detailed on Reference #8（page 13, line35）

4. The number of patients with severe pruritus is small (n=8) and therefore, meaningful associations with severe pruritus may not be appreciated.

**Response：**

In this study, there were a total of 148 patients with uremia and eight patients with severe skin pruritus, accounting for 5.4% of the total patients. 2012-2015 DPPSO study[3] surveyed 7,629 people, divided into five groups (not at all, somewhat, moderately, Very Much, Extremely) according to the degree of itchy skin. Extreme Itching accounted for 7%, which is slightly higher than 5.4% of patients with severe Itching in this study. The reason for the differences may lie in the different criteria used to assess the degree of pruritus in the patient's skin.

This section is detailed on page 14, lines 37-43

**Reviewer #2**

In this study, the authors studied uremic pruritus using the five-dimensional itch scale in 148 adult patients of end-stage renal disease receiving hemodialysis and report the prevalence of 40.5% and association with parathormone (PTH), hemoglobin and high-sensitive C reactive protein (hs-CRP) on bivariate analysis and hs-CRP alone on multiple logistic regression. The study seems to be designed logically, conducted scientifically and analyzed with due statistical rigour. However, the manuscript is lengthy, does not have a smooth flow of information and requires rewriting as per the STROBE checklist. Major observations are described below. Minor observations are inserted in the uploaded version of the manuscript as “sticky notes” at appropriate places.

**Response:**

(1) We have rewritten the paper according to the STROBE checklist, details of which can be found in the attached STROBE checklist.
(2) Concerning some minor observation, we have followed your comments with deep appreciation.

**Major observation:**

1. The introduction should briefly summarize the present state of knowledge on uremic pruritus, highlight the gap in understanding the study sought to address and state the study’s objective. Comparison with previous studies (measurement of severity of Itching) should be reserved for discussion.

**Response:**

The reviewer's suggestions were excellent, and we have corrected them.

This section has been moved to page 15, lines 23-29.

2. The stated aims of the study are to “1. explore the prevalence of UP in maintenance HD patients and the demographic and clinical characteristics of pruritus patients. Explore determinants of higher pruritus intensity based on participants’ clinical and biochemical parameters and their pruritus assessments by 5DIS at baseline”. The second aim is somewhat unclear. Were characteristics of patients with higher pruritus intensity to be compared with those of patients with of lower pruritus intensity or with those of patients without pruritus? Clear statement in the PICO format is suggested. What is the novelty of the research question? This needs to be justified.

**Response:**

**objectives**

(1). explore the prevalence of UP in maintenance HD patients and the demographic and clinical characteristics of pruritus patients.

(2). analyze the relationship between high-sensitivity C-reactive protein and different degrees of skin itching.

This section has been moved to page 2, lines 28-32.

3. The setting of the study may be described in “Methods”.

**Response:**

This section is detailed on ***Methods (study setting)***, page2, lines 38 & page3, lines 1-5.

4.How were biochemical parameters such as serum calcium, phosphorus, creatinine, etc assayed? The detailed methods need not be described but should be referenced.

**Response:**

We assayed biochemical indicators such as Ca, P, iPTH, Hb, ALB, sCr, Bun, FER, sp/KTV and hs-CRP. The hospital's central laboratory performed all of the laboratory tests, and auto-analyzers were used to determine biochemical data, iPTH was measured with Roche second-generation assay. The clinical biochemical indicators are based on the data within three months from the survey day. If there is no data within three months, the patient is required to re-test. If the patient is unwilling to cooperate with the test, it is a missing value. spKT/V（single pool KT/V）=-$\ln\left( R-0.008t \right)+\left( 4-3.5R \right)*\left( \Delta BW/BW \right)$. Note: K refers to Blood urea clearance rate of dialyzer（L/h）, t is the dialysis time (h), and V is the distribution volume of urea (V). R is the ratio of blood urea nitrogen after dialysis to blood urea nitrogen before dialysis; t is single dialysis time in h; ∆BW is the weight change value before and after dialysis, i.e. ultrafiltration, unit L; BW is weight in kg. Blood collection requirements: blood samples before dialysis from the artery end of the vascular path, after dialysis before blood sample collection to stop ultrafiltration, reduce blood flow of 50 ml/min, wait 15 seconds after blood collection from the artery as a blood sample after dialysis.

This section is detailed on page 3, lines 36-41 & page 4, lines 1-7.

5.The study reports no significant association of uremic pruritus with factors such as calcium, phosphorus, creatinine and albumin. However, the sample size calculation was made to estimate the prevalence of uremic pruritus. Could the study have been under powered for the study of these factors?

**Response:**

Some studies suggested that UP is related to Ca, P, ALB, and uremic toxins[11, 20]. Nevertheless, contradictory data on the impact of Ca and P have been reported[21]. In this study, no statistically significant differences were found in Ca, P, ALB, and creatinine when comparing groups of patients with different degrees of skin pruritus. There may indeed be a lack of test power（Since the data for these factors are non-normally distributed, the exact distribution is unknown. Based on statistical theory, it is not possible to calculate the true test power with certainty.）, which is considered to be related to the small sample size of this study and the unbalanced sample size among the groups. However, single-factor logistic regression analysis also did not find an association between these factors and the degree of skin pruritus. Therefore, these factors were ultimately not included in the multifactor logistic regression analysis. In fact, despite various substances have been discussed as potential pruritogens in chronic renal diseases, a causal relationship could never be established[22].

This section is detailed on page 15, lines 14-22.

6.The following factors influencing uremic pruritus have not been studied: diet, insulin resistance, dialysis adequacy, interleukins 6 & 2, and κ opioid receptor distribution. These need to be acknowledged as limitations.

**Response:**

Firstly, this study was a single-center study with a limited number of people on hemodialysis, and therefore the number of people included in the study was limited. Many factors influence skin itching in uremic patients, and it is not practical to include all relevant aspects of the study. Secondly, there is the issue of patient compliance. For example, to study diet factor, we need to distinguish between vegetarian and non-vegan patients, which requires strict control of the patient's diet. For insulin resistance, if the HOMA model is used to assess the resistance of the body to insulin (HOMA-IR), fasting blood glucose and fasting insulin levels need to be measured based on the formula HOMA-IR=FPG×FINS/22.5, therefor, fasting blood needs to be collected before hemodialysis. Some patients are difficult to cooperate, the missing value of the study will be further increased. Thirdly, for dialysis adequacy, we included a urea clearance index (kt/v) to assess this. In conclusion, it is essential to acknowledge the limitations of this study.

This section is detailed on page 13, lines 17-19.
