# Supplementary material for: Determinants of the intensity of uremic pruritus in patients receiving maintenance hemodialysis: A cross-sectional study
Source: PLoS One. 2021 Jan 20;16(1):e0245370. doi: 10.1371/journal.pone.0245370 (PMC7817000; doi:10.1371/journal.pone.0245370)
Supplement: Attachment — Submitted filename: Response to Reviewers.docx [file pone.0245370.s006.docx]

Dear Dr Girish Chandra Bhatt, MD, FASN and Reviewers

Thank you for giving us the opportunity to submit a revised draft of the manuscript “Determinants of the intensity of uremic pruritus in patients receiving maintenance hemodialysis: A cross-sectional study” for publication in the Journal of PLOS ONE.

We have incorporated all of the suggestions made by the reviewers. Those changes are highlighted in the revised manuscript with tracked changes. Please see a point-by-point response to the reviewers’ comments and concerns. Page numbers and line numbers in the original manuscript are shown in black and bold; page numbers and line numbers in revised manuscript with tracked changes are shown in normal type.

We hope that the revised manuscript is acceptable manuscript for publication in your Journal.

Sincerely yours,

Jian-Hui Zhao

**Response to academic editor**

1. **If you would like to make changes to your financial disclosure, please include your updated statement in your cover letter. Guidelines for resubmitting your figure files are available below the reviewer comments at the end of this letter.**

Response: we would not like to make changes to our financial disclosure.

2. **If applicable, we recommend that you deposit your laboratory protocols in protocols.io to enhance the reproducibility of your results. Protocols.io assigns your protocol its own identifier (DOI) so that it can be cited independently in the future. For instructions see:** [**http://journals.plos.org/plosone/s/submission-guidelines#loc-laboratory-protocols**](http://journals.plos.org/plosone/s/submission-guidelines#loc-laboratory-protocols)

Response: Our study is a clinical cross-sectional survey study and does not involve complex laboratory procedures and clinical research methods. However, we have found a simple formula for calculating normalized protein catabolic rate (nPCR). In this formula, as long as there is urea nitrogen before hemodialysis and the KT/V result of single hemodialysis, nPCR can be calculated accurately. We deposit this method on the protocols.io website to get the doi number (<https://dx.doi.org/10.17504/protocols.io.bp94mr8w>) [PROTOCOL DOI].

3. **While revising your submission, please upload your figure files to the Preflight Analysis and Conversion Engine (PACE) digital diagnostic tool,** [**https://pacev2.apexcovantage.com/**](https://pacev2.apexcovantage.com/)**. PACE helps ensure that figures meet PLOS requirements. To use PACE, you must first register as a user. Registration is free. Then, login and navigate to the UPLOAD tab, where you will find detailed instructions on how to use the tool. If you encounter any issues or have any questions when using PACE, please email PLOS at** [**figures@plos.org**](mailto:figures@plos.org)**. Please note that Supporting Information files do not need this step.**

Response: We have updated Fig 1, but Fig 2-4 do not require any changes. PACE can ensure that these figures are PLOS compliant.

**Response to Reviewer**

**Reviewer #1**

**The authors have revised the manuscript in response to the initial comments. Improvements include addition of other variables that have been associated with pruritus into their analysis along with data to support their proposed use of the 5D score to assess pruritus. Overall, the manuscript adds to existing reports of hsCRP’s association with uremic pruritus. One minor comment to consider is that the nPCR can be obtained from the kinetic modeling used to obtain spKt/V. But since the BUN levels were not different across pruritus severity, it is unlikely that the nPCR will be different.**

**Response:**

Thank you for your suggestions on the data integrity and statistical analysis aspects of your paper, we see that you have given us a kind reminder in your comments. Here are the answers to your concerns.

Following your suggestion, we have added a variable (nPCR) in the revised manuscript, based on pre-dialysis blood urea nitrogen levels in combination with spKT/V, to introduce the formula: $nPCR((g/kg)/d)=C_{0}/[a+b\times KT/V+c\div(KT/V)] +0.168$,where C_0_ indicates pre-dialysis blood urea nitrogen in mg/dl (1mmol/L urea nitrogen is equal to 2.802mg/dl). a, b, c has different coefficients depending on the time of the dialysis schedule. In our blood purification center, patients are dialyzed three times a week, either on Mondays, Wednesdays, Fridays or either Tuesdays, Thursdays, Saturdays, so that the time between the first dialysis sessions at the beginning of the week is longer. The following formula is used: beginning-of-week: $nPCR(g/kg/d)=C_{0}/[36.3+5.48\times KT/V+53.5\div(KT/V)] +0.168$.

Our statistical analysis showed that patients with pruritus have a higher nPCR level compared to patients without pruritus (p=0.028), however, there is no statistically significant difference between patients with different levels of pruritus (p=0.136). Furthermore, the single factor analysis also did not show a statistically significant correlation, thus the results of the multifactorial analysis did not change and the conclusions of the paper were unchanged.

This section was shown in lines 7-13, page 4 in revised manuscript with tracked changes and the detailed data are shown in Tables 1-3.

**Reviewer #2**

**The authors have rewritten the manuscript and it reads much more clearly. Small changes are suggested. These have been indicated as "sticky notes" at the appropriate places in the uploaded version of the manuscript.**

**Response:**

We appreciate your suggestions regarding the language structure of our paper, which is very conducive to improving the quality of the paper, and the following is an itemized response to your small comments.

**1. Line 25, page 2: diabetes mellitus (avoid unnecessary capitalization)**
This phrase was modified according to the comment. (Line 24, page 2).

**2. Line 29, page 2: Objectives (headings should be capitalized)**

Thank you for pointing this out. As you suggested, we have corrected the “objectives” to “Objectives”. (Line 28, page 2).

**3. Line 30 page 3: we feel sorry for our carelessness in our resubmitted manuscript, the error is revised.**

We have corrected “BMI” to “Body Mass Index (BMI)”. (Line 27, page 3).

**4. Line 31 page 3:**

We have corrected “normal patients with BMI” to “patients with normal BMI”. (Line 28, page 3).

**5. line 36 page 3:**

Thank you for pointing this out. As you suggested, we have corrected the “Place of residence “to “place of residence”. (Line 33, page 3).

**6. line 39 page 3: Avoid using the term "such as".**

“such as “is replaced with “comprised of”. (line 36 page 3)

**7. line 4 page 4:**

Thank your suggestions, we have corrected it. (line 41 page 3)

**8. line 12 page 4: The section on bias may be included into the discussion as part of limitations of the study.**

The section on bias was included into the discussion as part of limitations of the study. (lines 19-27, page13).

9. **line 27 page 4**

This phrase was modified according to the comment. (Line 14, page 4).

**10. line 30 page 4**

This phrase was modified according to the comment. (Line 17, page 4).

**11. line 34 page 4: The section on "quantitative variables" may be included in "statistical methods". This may be rewritten with a more logical flow.**

The section on "quantitative variables" have been included in "statistical methods" according to your suggestions and we also rewritten this section. (lines 22-37 page 4)

**12. line 9 page 5: Is it 4 missing values in two patients and therefore a total of 8 missing values?**

Respond: yes.

**13. line 14 page 5: There were a total of 180 patients. Why were 20 patients excluded. This should be clarified.**

Our blood Purification Center has a total of 180 patients. 20 patients were excluded, and 10 of them were unwilling to participate in the survey. 5 were peritoneal dialysis combined with hemodialysis, but hemodialysis was only treated once a week, and the remaining 5 were scheduled for 2 dialysis a week. Therefore, a total of 160 patients who regularly underwent hemodialysis three times a week were included in this study from March 2019 to June 2019. (line40 page 4 & lines 1-5 page 5). Besides, we updated Fig 1.

**14. Duplication should be avoided. For instance, if the number of males is shown, the number of females need not be shown.**

We have modified these phrase, See Table 1 for details.

**15. The values are shown as n (%) or median (IQR). This should be specified in the table.**

Continuous variables with skewed data are summarized as the median (interquartile range); Dichotomous or categorical data are summarized as counts (percentages).

As shown in the section on notes in Table 1 for details.

**16. Line 2 page 13:** **This section of key result in discussion may be rewritten with brevity.**

The section has been rewritten and streamlined. (lines3-11 page 13).

**17. Line 22 page 13: This section may be rewritten with brevity.**

The section has been rewritten and streamlined. (lines 29-40 page 13 & lines1-44 page 14 & lines 1-35 page 15).
